# Genetic screens of imaging-derived kidney volumes identify genes linked to kidney function

**DOI:** 10.1016/j.kint.2025.08.038

**Published:** 2025-10-10

**Authors:** Sara Monteiro-Martins, Yong Li, Oleg Borisov, Atlas Khan, Wilfried Reichardt, Stefan Haug, Elias Kellner, Martin Buechert, Elisabeth Ott, Maximilian F. Russe, Fabian Bamberg, Krzysztof Kiryluk, Peggy Sekula, Marco Reisert, Anna Köttgen

**Affiliations:** 1 Institute of Genetic Epidemiology, Faculty of Medicine and Medical Center–University of Freiburg, Freiburg, Germany; 2 Division of Nephrology, Department of Medicine, Vagelos College of Physicians & Surgeons, Columbia University, New York, New York, USA; 3 Division of Medical Physics, Department of Diagnostic and Interventional Radiology, University Medical Center Freiburg, Faculty of Medicine, University of Freiburg, Freiburg, Germany; 4 Core Facility Magnetic Resonance Development and Applications Center (MRDAC), Department of Diagnostic and Interventional Radiology, University Medical Center Freiburg, Faculty of Medicine, University of Freiburg, Freiburg, Germany; 5 Department of Medicine IV, Faculty of Medicine, Medical Center–University of Freiburg, Freiburg, Germany; 6 Department of Diagnostic and Interventional Radiology, University Medical Center Freiburg, Faculty of Medicine, University of Freiburg, Freiburg, Germany; 7 Department of Stereotactic and Functional Neurosurgery, Medical Center - University of Freiburg, Faculty of Medicine, University of Freiburg, Freiburg, Germany; 8 Department of Epidemiology, Johns Hopkins Bloomberg School of Public Health, Johns Hopkins University, Baltimore, Maryland, USA

**Keywords:** abdominal MRI, GWAS, imaging, kidney disease, kidney function, kidney volume

## Abstract

**Introduction:**

Chronic kidney disease (CKD) is defined as sustained abnormalities in kidney function or structure. Genetic studies of CKD have largely focused on kidney function markers such as estimated glomerular filtration rate (eGFR). We hypothesized that genome-wide association studies (GWAS) of magnetic resonance imaging (MRI)-based kidney sub-volumes could provide insights into CKD risk genes complementary to the study of eGFR.

**Methods:**

Total kidney volume (TKV) and sub-volumes for cortex, medulla, and sinus were derived from abdominal MRIs of 38,816 United Kingdom Biobank participants of European ancestry using a trained convolutional neural network. GWAS was performed for body surface area-normalized kidney volumes and eGFR for comparison. Potentially causal genes at each locus were prioritized using a developed annotation pipeline. We assessed locus overlap between volumes, biomarker-based kidney function, and clinical traits using colocalization analyses. Annotated genes were further characterized through enrichment analyses, molecular and clinical annotations, including a screen for rare, putative loss-of-function variants.

**Results:**

GWAS for 9,803,932 common genetic variants identified 34 significant loci for TKV, 24 for medulla, 26 for cortex, and 71 for sinus, compared to 32 for eGFR. Prioritized genes for cortex and medulla volumes showed corresponding tissue-specific expression and were enriched for kidney development- and hypoxia-related pathways. Genetic effect sizes of significant index single nucleotide polymorphisms for TKV, cortex, and medulla volumes correlated positively with those for eGFR. Some loci such as *PKHD1* and *BICC1* were strongly associated with kidney volumes but not eGFR. Integration with disease information revealed that rare, putative loss-of-function variants in *BICC1,* and common variants with regulatory potential, are associated with increased risk for CKD and dialysis, which was not identified in a previous GWAS of eGFR

**Conclusions:**

Our investigation shows that genetic findings of kidney structure can complement kidney function studies and reveal previously unrecognized CKD risk genes in the population.

## Lay Summary

We performed a genetic analysis of kidney subvolumes, derived from whole body images of 38,816 participants of European ancestry in the UK Biobank. We aimed to uncover chronic kidney disease (CKD) risk genes that may be missed by traditional measures of kidney function, such as estimated glomerular filtration rate. This approach is a complementary approach to investigating the genetics of kidney disease and allowed us to detect genetic variants associated with kidney morphology in adults. For example, we identified genes related to kidney development and *BICC1* as a CKD risk gene in the population. Future research integrating insights from imaging-derived measures of kidney structure may improve CKD risk stratification and enable research into molecular mechanisms relevant to kidney growth and repair.

Chronic kidney disease (CKD) affects ≈10% of the global adult population.^1,2^ Despite its high prevalence, CKD is underrecognized, and its underlying molecular programs remain incompletely characterized. CKD is defined as persistent abnormalities of kidney structure or function.^3^ Although kidney function is commonly quantified as the estimated glomerular filtration rate (eGFR) using blood biomarkers, such as creatinine or cystatin C, population-scale information on kidney structure is rarely available. Therefore, genetic screens aimed at elucidating molecular mechanisms related to CKD have concentrated on kidney function measures, such as eGFR.^4-7^ Such screens may reveal not only genes related to filtration but also to biomarker metabolism, while missing genes related to abnormalities in kidney structure. Moreover, because serum creatinine or cystatin C levels only increase after ≈50% of filtration function has been lost,^8^ genes related to early CKD may be missed. Thus, genetic screens of measures of kidney structure in large populations are an important research avenue to reveal additional genes potentially related to CKD.

Recently emerging, large population samples with whole-body imaging include the UK Biobank (UKB), a biomedical research project that collects and stores health information, biological samples, and genetic information from >500,000 participants in the United Kingdom.^9^ Participants are offered a protocol that contains whole-body magnetic resonance imaging (MRI).^10^ Another resource is the German National Cohort (NAKO) Health study,^11^ where whole-body MRI was performed for 31,578 participants.^12,13^ In both studies, the MR images contain visualization of the kidneys and can be used to segment markers of kidney structure, such as kidney volumes.^14,15^

Only 1 large-scale, hypothesis-generating genome-wide association study (GWAS) of MRI-derived total kidney volume (TKV) has been published to date.^14^ Using data from the UKB, the investigators found that lower TKV was associated with lower eGFR and increased odds of CKD, which we independently confirmed using data from the NAKO study.^15^ The GWAS of TKV performed by Liu *et al.* identified 12 associated loci, including 5 that contained genes previously linked to eGFR.^4^ The study did not, however, investigate different kidney compartments, although different compartments of the kidney, such as the cortex and medulla, have different physiological roles. Thus, GWAS of kidney subvolumes reflecting these compartments offers the opportunity to discover genes missed in the analysis of TKV and to evaluate whether identified genes can be linked to compartment-specific physiological functions. Last, the integration of molecular readouts of gene function, such as transcriptomics or proteomics, offers the opportunity to establish hypotheses about mechanisms linking genetic variation to kidney volumes.

Here, we aimed to explore the genetic architecture of MRI-derived kidney subvolumes from 38,816 UKB participants of European ancestry followed by integration with molecular and clinical information to uncover and characterize genes and mechanisms related to kidney structure in adults, and to gain complementary insights into those obtained from eGFR.

## METHODS

### Study population

The UKB is a large, population-based study consisting of >500,000 participants, aged 40 to 69 years at recruitment, starting in 2006 with Research Ethics Committee approval. This rich dataset includes genetic, clinical, and lifestyle information, along with extensive imaging data.^9,10^ DICOM files corresponding to abdominal MRIs acquired using the Dixon protocol were captured from a volunteer subset of the UKB without contraindications using a Siemens Aera 1.5-T MRI scanner, as described previously.^10^ At the time of analysis, the obtained dataset comprised a total of 53,035 MRIs from 48,135 participants, which we processed for analysis.

### Image processing and kidney volume assessment

We used a previously trained and validated convolutional neural network^16,17^ developed and transferred from the NAKO Health Study^15^ to segment the kidney compartments cortex, medulla, and sinus. After model application to the dataset, results were visually inspected, followed by model retraining to optimize accuracy, as described in detail in the Supplementary Methods. Analyzed kidney volumes were computed on the basis of the refined segmentations.

### Genome-wide association studies

UKB genotypes used in this project were generated using the UK Biobank Axiom Array and the Affymetrix UK BiLEVE Axiom Array, and imputation was performed using the Haplotype Reference Consortium, the UK10K, and the 1000 Genomes phase 3 reference panels.^9^ GWAS of autosomal variants was performed using linear regression implemented in Regenie (version 3.2.9).^18^ Step 1 included 509,385 directly genotyped variants with minor allele frequency >1%, minor allele count >100, missingness <10%, and Hardy-Weinberg equilibrium *P* > 1e – 15. Cross-validated ridge regression was performed in blocks of 1000 single-nucleotide polymorphisms (SNPs). Step 2 used imputed variants derived from the UKB imputed genotype version 3.^9^ Analysis was performed using a block size of 400, a minimum imputation information (INFO) score of 0.3 to ensure variant imputation of acceptable quality, a minor allele count of 300, and a maximum of 99 categorical levels. After filtering for minor allele frequency >1%, 9,803,932 variants were included. Analyses were adjusted for age, age^2^, genetic sex, assessment center, and the first 10 genetic principal components to account for potential population stratification, as estimated from the UKB Pan-ancestry (PAN) project.^19^

The summary statistics were first lifted to the hg38 reference genome using the liftover R package.^20^ Significant loci were identified by iteratively selecting the most significant variant in the summary statistics with a *P*-value cutoff of 5e – 8 and defining a region as a 1 Mb window centered around its position. Nearby regions were merged if their boundaries were within 1 Mb of the newly identified region, adjusting the start or stop positions as needed. The variant with the lowest *P* value per region was designated the index SNP.

### GWAS annotation

To prioritize the most probable causal genes for each independent GWAS association signal, we developed an annotation pipeline that integrates multiple lines of evidence, building on and enhancing the ProGeM framework.^21^ Our approach incorporates gene proximity, functional variant annotations using Ensembl 101 Variant Effect Predictor,^22^ genetic colocalization with expression and protein quantitative trait loci, and linkage disequilibrium–based gene overlap, to generate a comprehensive scoring system for gene prioritization. The individual sources of evidence and gene scoring are detailed in the Supplementary Methods, with source code available at https://github.com/genepi-freiburg/GWASannotation.

Additional methods related to phenotype definitions, statistical analyses, enrichment analyses, and the characterization of genetic associations with molecular and clinical traits can be found in the Supplementary Methods.

## RESULTS

### Study population and distribution of volumes

**Figure 1 ∣:**
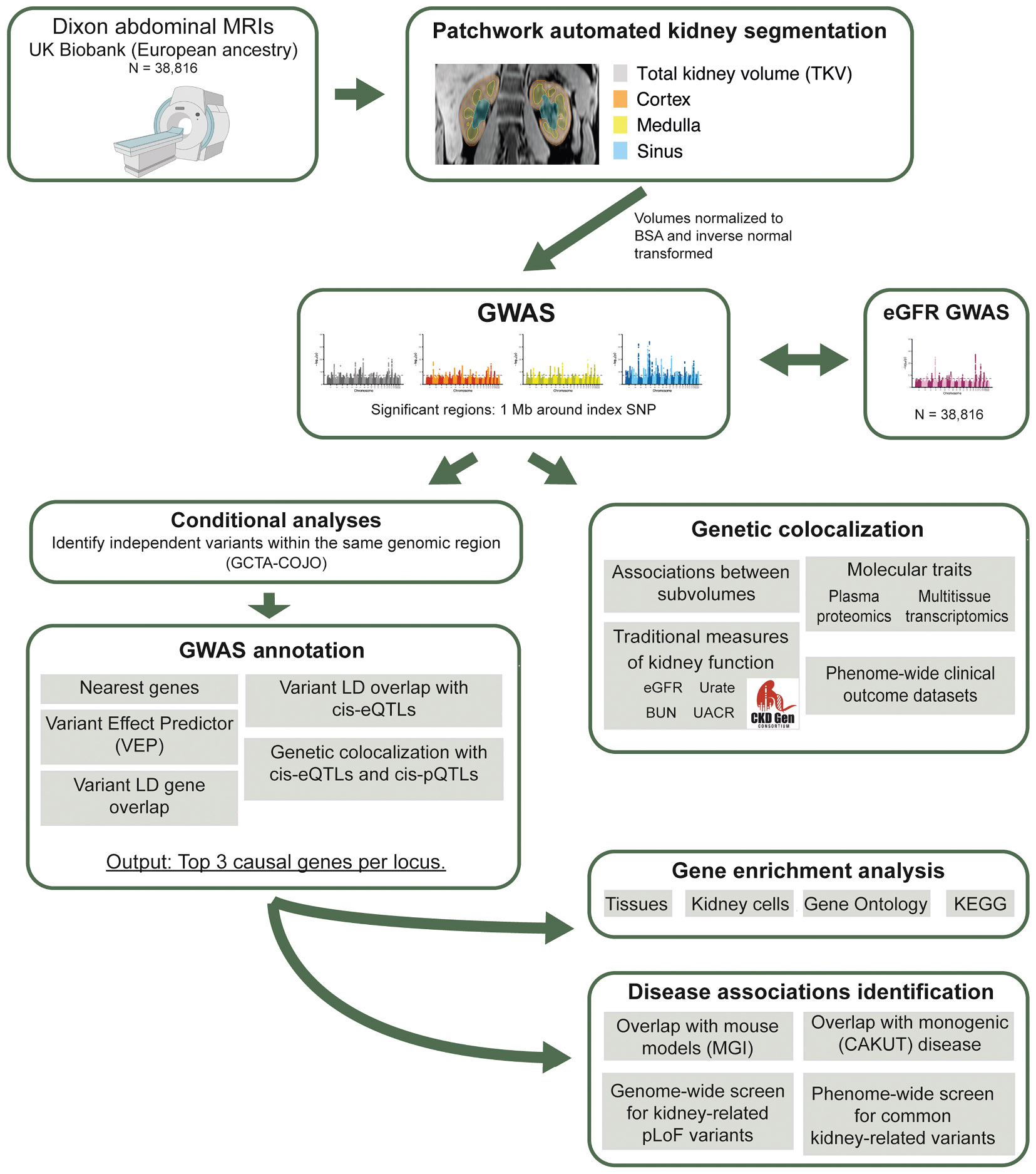
Overview of the study design. Schematic representation of the genome-wide association study (GWAS) of imaging-derived kidney volumes from 38,816 individuals of European ancestry from the UK Biobank and downstream analysis. BSA, body surface area; BUN, blood urea nitrogen; CAKUT, congenital abnormalities of the kidney or urinary tract; eGFR, estimated glomerular filtration rate; eQTL, expression quantitative trait loci; KEGG, Kyoto Encyclopedia of Genes and Genomes; LD, linkage disequilibrium; MRI, magnetic resonance imaging; pLoF, putative loss of function; pQTL, protein quantitative trait loci; SNP, single-nucleotide polymorphism; UACR, urine albumin-creatinine ratio.

This study (Figure 1) included 38,816 UKB participants of European ancestry with an average age of 64.6 years, 51.4% of whom were women. The mean TKV was 310.5 ml (SD, 65.4 ml; Supplementary Table S1). Mean volumes for renal cortex were 233.7 ml (SD, 52.9 ml); for medulla, 76.8 ml (SD, 21.2 ml); and for sinus, 38.6 ml (SD, 15.5 ml). When normalized to body surface area, the primary outcomes for this study, mean TKV was 164.1 ml/m^2^ (SD, 25.3 ml/m^2^); cortex, 123.1 ml/m^2^ (SD, 18.9 ml/m^2^); medulla, 41 ml/m^2^ (SD, 11.1 ml/m^2^); and sinus, 20.2 ml/m^2^ (SD, 7.1 ml/m^2^). The mean eGFR was 91.5 ml/min per 1.73 m^2^ (SD, 12.2 ml/min per 1.73 m^2^). Distributions of all volumes and covariates are provided in Supplementary Table S1 or depicted in Supplementary Figure S1.

### Kidney volumes are heritable and reveal significantly associated genetic variants

The genetic contribution to kidney structure was assessed by estimating the variance in kidney subvolumes explained by genotyped SNPs (Methods). The proportion of variance attributable to additive genetic effects was 33% for TKV, 32% for cortex, 29% for medulla, and 47% for sinus (Supplementary Table S2), compared with 26% for eGFR. These results highlight a substantial genetic contribution to kidney subvolumes and motivate subsequent GWAS. Phenotypically, TKV, cortex, and medulla exhibited moderate to strong pairwise correlations (*r* = 0.38–0.91), whereas their correlation with sinus was lower (*r* = −0.14 to 0.49). The eGFR displayed weaker correlations with kidney sub-volumes, ranging from *r* = 0.01 (sinus) to *r* = 0.39 (medulla). Genetic correlation estimates closely mirrored these patterns, with high genetic correlations among TKV, cortex, and medulla (*r_g_* = 0.65–0.95), lower correlations with sinus (*r_g_* = −0.06 to 0.41), and moderate genetic correlations between eGFR and kidney subvolumes (*r_g_* = 0.21–0.64; Supplementary Figure S2).

**Figure 2 ∣:**
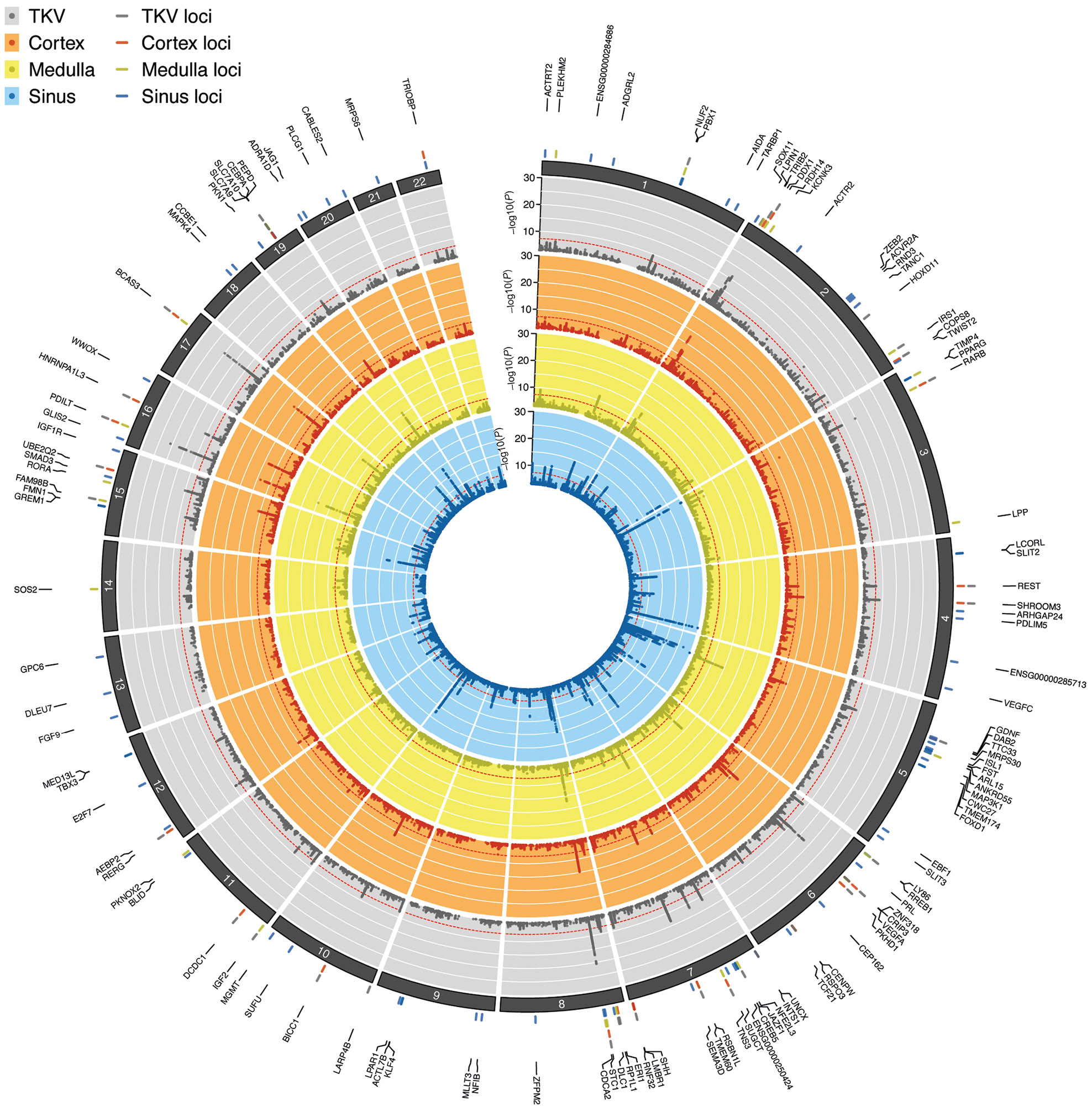
Genome-wide association study (GWAS) identifies significant loci for kidney subvolumes. The circos plot shows genome-wide association results for total kidney volume (TKV), cortex, medulla, and sinus volumes (N = 38,816). Each band represents −log_10_(*P*) ordered by chromosomal position: gray for TKV, orange for cortex, yellow for medulla, and blue for sinus. The red dashed line indicates genome-wide significance (*P* = 5 e – 8). The outer band highlights significant loci for each trait, with gene names representing the prioritized gene selected using the GWAS annotation pipeline (Methods).

GWAS of TKV identified 34 loci that contained at least 1 significant variant (*P* < 5e – 8), whereas the analyses of cortex, medulla, and sinus volumes identified 24, 26, and 71 significant loci, respectively (Figure 2; volume-specific Manhattan plots in Supplementary Figure S3A; and regional association plots in Supplementary Figures S4-S7). Quantile-quantile (QQ) plots and linkage disequilibrium score regression intercepts indicated no evidence of unmodeled stratification (Supplementary Figure S3B).

Compared with results from the previously published GWAS of TKV,^14^ 10 of 12 index variants reported by Liu *et al.* also showed genome-wide significant, direction-concordant associations in our study with at least 1 sub-volume in a sensitivity analysis without correction for body surface area, the published study’s main phenotype (Supplementary Table S3). Some differences in the remaining results were likely attributable to differences in the segmentation model and study sample.

Subsequent colocalization analyses were performed to identify genetic regions in which the associations with the 4 different kidney subvolumes were likely caused by the same underlying variant (colocalization defined as posterior probability of hypothesis 4 [PPH4] > 0.8; Supplementary Table S4). Although most TKV- and cortex-associated loci colocalized with at least 1 other kidney subvolume, medulla (9 of 26 loci) and especially sinus volume (67 of 71 loci) contained a substantial number of volume-specific significant associations (Supplementary Figure S8).

Conditional analyses to refine association signals and investigate the presence of multiple independent variants within the same locus (Methods) identified 5 of 34 TKV loci, 4 of 24 cortex loci, 2 of 26 medulla loci, and 12 of 71 sinus loci with >1 independent SNP (Supplementary Table S5 and Supplementary Figures S4-S7).

The most probable causal gene for each independent association signal was prioritized using an annotation pipeline, as described in detail in the Supplementary Methods. There were 36 prioritized genes for TKV, 26 for cortex, 28 for medulla, and 81 for sinus (Figure 2 and Supplementary Table S5); some independent SNPs shared the same prioritized gene. The proportion of prioritized genes that were also the closest to the index variant ranged from 85% for TKV to 93% for medulla.

### Comparison of kidney volume associations with eGFR reveals overlapping and distinct genetic architecture

**Figure 3 ∣:**
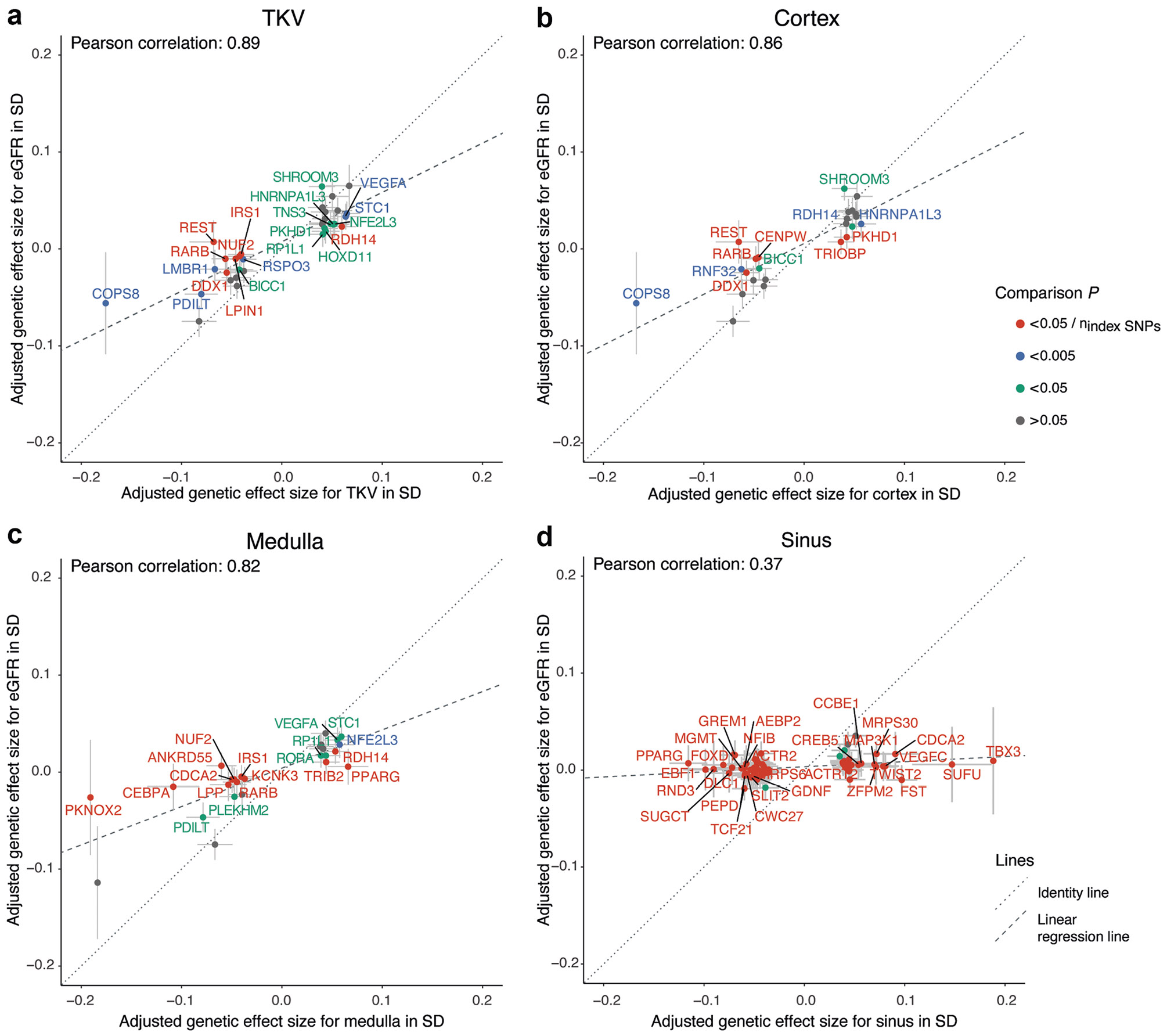
Genetic effect size comparisons reveal loci with distinct associations with kidney volumes and estimated glomerular filtration rate (eGFR). Comparison of genetic effect sizes between eGFR and body surface area–standardized (**a**) total kidney volume (TKV), (**b**) cortex, (**c**) medulla, and (**d**) sinus volumes. Genes are labeled if the comparison *P* was < 0.05. (**d**) For sinus volume, labeled genes are additionally filtered for an absolute genetic effect size for association with sinus volume ≥ 0.05. Each point represents an index single-nucleotide polymorphism (SNP), with colors indicating the level of statistical significance of the difference in effect sizes between the 2 genome-wide association study analyses (Methods). Genetic effect size comparison was performed as outlined in the Supplementary Methods. Units correspond to the SD of the respective inverse normal transformed traits. Panels display (**a**) 34 SNPs for TKV, (**b**) 24 for cortex, (**c**) 26 for medulla, and (**d**) 71 for sinus.

GWAS of eGFR using data from the same 38,816 UKB participants identified 32 significant loci (Supplementary Figure S9). The effect sizes of subvolume-associated index SNPs were strongly correlated with their estimates from eGFR GWAS (Pearson correlations of 0.89 for TKV, 0.86 for cortex, and 0.82 for medulla), with the exception of sinus volume (*r* = 0.37; Figure 3). Among 34 TKV-, 24 cortex-, and 26 medulla-associated index SNPs, 10, 8 and 6, respectively, showed genome-wide significant (*P* < 5e – 8) associations with eGFR. Interestingly, genetic effect sizes for 21 TKV, 12 cortex, 19 medulla, and 69 sinus index variants were significantly different (comparison *P* < 0.05) from their associations with eGFR, mostly with larger absolute effects for kidney subvolumes (Figure 3 and Supplementary Table S6). Prioritized genes for which the index variant did not show association with eGFR of at least suggestive significance (*P* < 1e – 5) included TKV- and cortex-associated *PKHD1*^23^ and *BICC1*,^24^ previously linked to monogenic cystic kidney diseases in humans or model organisms (see below). Most genes with index variants that showed little or no association with eGFR, however, have not yet been linked to human cystic diseases, although *RARB* and *REST* have been linked to kidney development^25^ and kidney cancer,^26^ phenotypes related to kidney size and growth. A range of additional sensitivity analyses indicated that associations between identified index variants and kidney volumes could not be attributed to their relation with eGFR or anthropometric measures (Supplementary Results, Supplementary Figures S10 and S11, and Supplementary Table S7).

### Enrichment analyses

**Figure 4 ∣:**
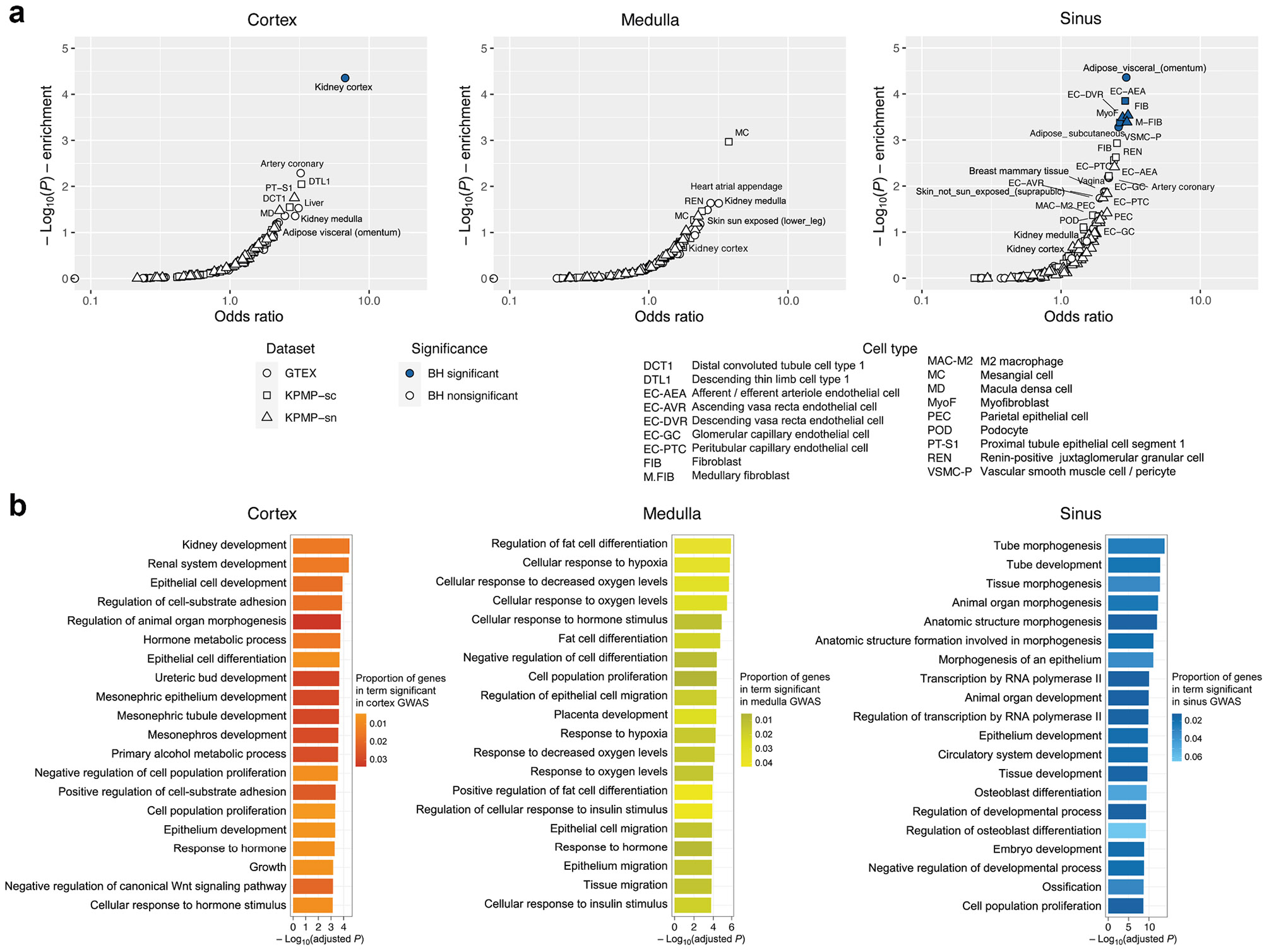
Distinct tissues, cell types, and biological processes are enriched for genes associated with kidney subvolumes. Enrichment analysis for the prioritized genes from genome-wide association study (GWAS) of cortex (left), medulla (middle), and sinus (right) volumes for (**a**) high gene expression in specific cell types based on Genotype-Tissue Expression (GTEx) project version 8 and the Kidney Precision Medicine Project (KPMP) single-cell (sc) and single-nucleus (sn) data as well as (**b**) biological processes based on Gene Ontology terms. (**a**) Tissues and cell types with empirical *P* < 0.05 are labeled. BH, Benjamini-Hochberg.

Enrichment analyses of the prioritized genes showed that cortex volume–related genes were highly expressed only in kidney cortex (odds ratio [OR], 6.73; *P* = 4.4e – 5; Methods), whereas sinus volume–associated genes were highly expressed in subcutaneous and visceral adipose tissue (ORs, 2.6 and 2.9, respectively; Figure 4a and Supplementary Table S8). Medulla volume–associated prioritized genes were highly expressed in the 9 kidney medulla tissue samples from the Genotype-Tissue Expression (GTEx) project (OR, 3.18), although this was not significant after correction for multiple testing. These findings were further supported by kidney cell type–specific overrepresentation analyses and single-cell expression patterns (Supplementary Table S8 and Supplementary Figure S12), implicating different proximal and distal tubular cell types for cortex-related genes, whereas medulla-associated genes showed stronger expression in the distal loop of Henle and thick ascending limb. Sinus-related genes were most prominently expressed in endothelial and other vascular-related cell types.

Moreover, prioritized genes were strongly overrepresented in pathways reflecting kidney- and embryonic development–related molecular functions, cellular components, and biological processes (Methods; Supplementary Table S9). Whereas genes associated with TKV and cortex volumes, including *BICC1, VEGFA, RARB, PKHD1, HOXD11*, and *SHH*, were most strongly overrepresented in terms related to kidney, renal system, tube, mesonephros, and epithelial development, genes related to medulla volume were overrepresented in hypoxia- and oxygen-related biological processes terms, including *PPARG, KCNK3, STC1, RORA*, and *VEGFA* (Figure 4). Genes related to kidney sinus volume were most strongly enriched for terms related to embryo, tissue, and anatomic structure development in general and to tube morphogenesis in particular (Supplementary Table S9). For example, genes related to ureteric bud development included *PBX1, SLIT2, GDNF, FOXD1, TCF21, GREM1, FMN1*, and *SMAD3*.

Last, we systematically tested whether the top 3 prioritized genes per independent association signal overlapped with genes known to result in abnormal kidney development or size when genetically manipulated in mice, or as causative or likely causative for congenital abnormalities of the kidney or urinary tract in humans or for cystic renal disease (Supplementary Table S10; Methods). Table 1 shows that 25 prioritized genes in volume-associated loci either overlapped with renal phenotypes in mice (N = 17), including top 1 prioritized *BICC1* and *TIMP4* for small kidneys, and *SLIT2, EBF1*, and *TCF21* for enlarged kidneys, or with human congenital abnormalities of the kidney or urinary tract (N = 4, sinus-related *PBX1, JAG1*, and *SLIT2* as well as cortex-related *SALL1*) or cystic renal disease (N = 4, *GLIS2, MKKS, PKHD1*, and *UMOD*). Index SNPs in 11 of these 25 genomic regions did not contain a variant associated with eGFR at *P* < 1e – 5 (Table 1).

### Shared genetic basis of kidney volumes with molecular traits

Genetic colocalization analyses were also performed to assess the evidence for a shared genetic basis with intermediate molecular traits, namely, gene expression in multiple human tissues,^5,27,28^ and circulating protein levels,^29,30^ as such connections may implicate underlying molecular links (Methods).

In 354 instances, a shared variant driving the association with ≥1 of the kidney volumes and the expression of a *cis* gene in the kidney and/or blood was implicated (88 for TKV, 58 for cortex, 25 for medulla, 183 for sinus; Supplementary Table S11). These observations are consistent with a mechanism whereby genetic variants with regulatory potential relate to kidney volumes via affecting transcript levels of the implicated gene. In addition, there were 1186 instances with evidence for a shared variant driving the associations with ≥1 of the kidney subvolumes and with the levels of circulating proteins encoded in *cis* (N = 9) or in *trans* (N = 1177; Supplementary Table S11). For instance, genetic variants in the known CKD risk locus *UMOD/PDILT* were strongly associated not only with TKV, cortex, and medulla volumes but also with the levels of multiple circulating proteins encoded in *trans* that are known to correlate with kidney function, including the filtration marker cystatin C,^31^ the tubular markers β2-microglobulin,^32^ α1-microglobulin,^32^ and trefoil factor 3 (TFF3),^33^ and the kidney injury marker KIM-1.^34^

We next evaluated the evidence for a shared basis with traditional kidney function traits using some of the largest publicly available datasets of GWAS meta-analyses of eGFR, blood urea nitrogen, serum urate, or the urine albumin-creatinine ratio performed by the CKDGen Consortium.^4,35-37^ Overall, 94 instances of shared genetic signals were identified, underlying the associations between kidney subvolumes (38 for TKV, 18 for medulla, 27 for cortex, and 11 for sinus) and traditional kidney function traits (12 for blood urea nitrogen, 48 for eGFR, 6 for UACR, and 28 for urate) (Supplementary Table S12 and Supplementary Figure S13). Although these findings support a shared genetic basis of kidney function and structure at the implicated loci, many kidney volume–associated loci were not associated with traditional markers of kidney function even in GWAS that had >10-fold greater sample sizes.

### Association of kidney volume–related variants and genes with human traits and diseases

**Figure 5 ∣:**
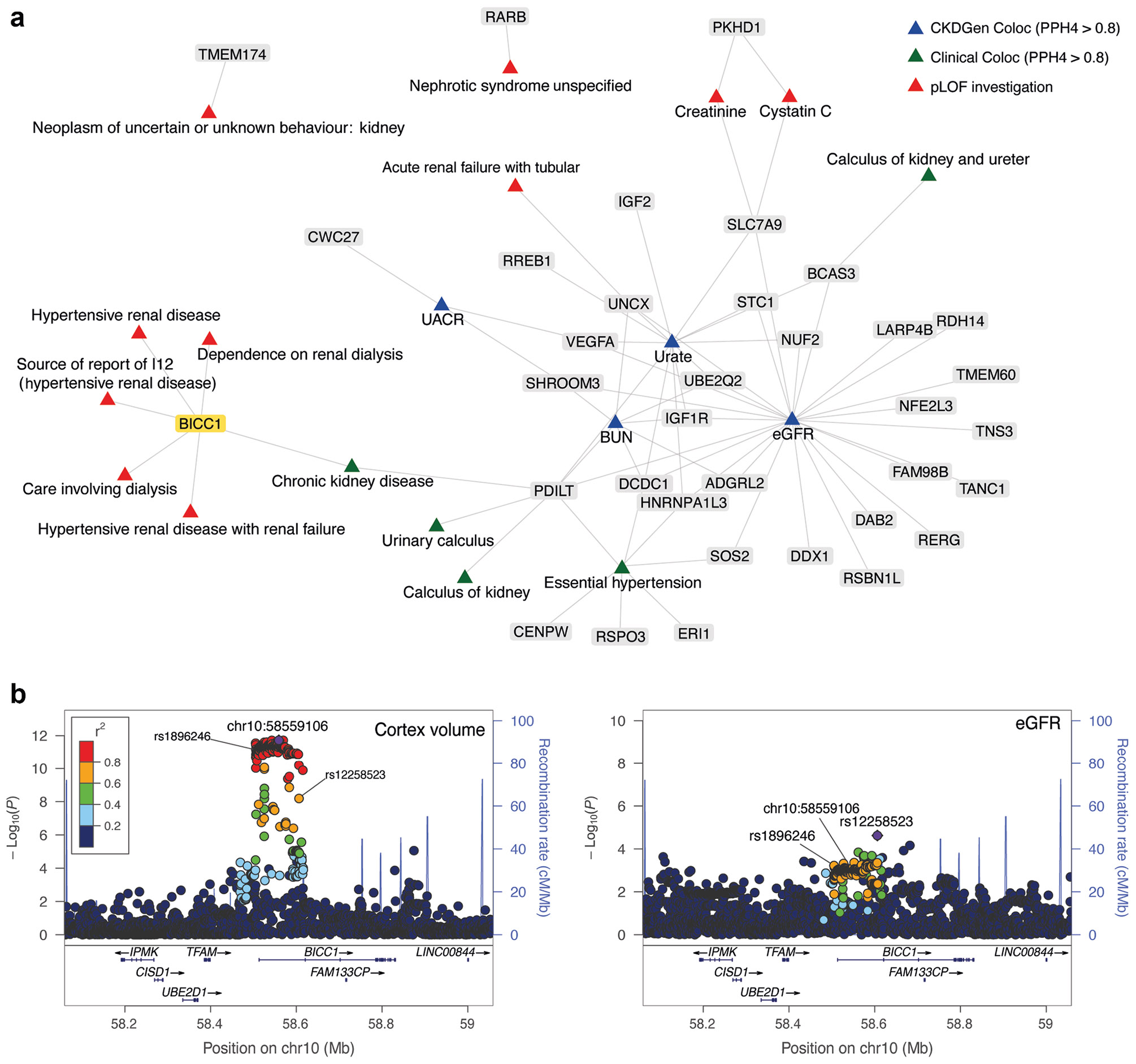
Kidney volume–associated genetic loci are associated with kidney-related phenotypes. (**a**) Network displaying top 1 prioritized genes and their relationships with kidney phenotypes by colocalization (Coloc) analysis with kidney traits from the Chronic Kidney Disease Genetics (CKDGen) Consortium (blue) and hundreds of clinical outcomes and traits from the UK Biobank and FinnGen (red), as well as with effects of rare, exonic putative loss-of-function (pLOF) variants (Methods). (**b**) Regional association plots for the *BICC1* locus. Single-nucleotide polymorphisms (SNPs) are plotted by position (b38) in a 500-kb window versus −log_10_(association *P*) from genome-wise association study of cortex volume (left) and estimated glomerular filtration rate (eGFR) (right; N = 38,816). The purple diamond highlights the most significant SNP for each association. SNPs are color coded to reflect their linkage disequilibrium with this SNP. Plots were generated using Locus Zoom.^40^ Chr, chromosome.

A shared genetic basis with human traits and diseases was tested based on UKB^9,38^ and FinnGen^39^ databases (Methods). We detected colocalization of ≥1 kidney subvolume(s) with kidney disease and urolithiasis at the *PDILT/UMOD* locus and between several other loci and kidney function–related diseases, such as hypertension, cardiovascular disease, and diabetes (Supplementary Table S13 and Figure 5a).^40^ Moreover, we tested whether rare, putative loss-of-function (pLoF) variants in any of the top 3 prioritized genes for each independent signal were associated at *P* < 1e – 5 with ≥1 of 29 kidney-related health outcomes or 4 kidney function measures (Methods). The well-established association between pLoF variants in *UMOD* (*PDILT/UMOD* locus) and CKD, as well as multiple CKD-related outcomes, can be considered proof of concept (Figure 5a and Supplementary Table S14). Additional CKD-associated genes included *SLC22A7*, consistent with the encoded protein’s function as a creatinine transporter, the main marker to estimate kidney function and stage CKD, as well as cortex- and TKV-related *BICC1* (*P* = 2.7e – 6; Supplementary Table S14), previously linked to cystic kidney disease in mice^41^ and in a case report of 2 children.^24^ Sinus volume–related genes in which pLoF variants were associated with adverse kidney outcomes included *UNCX* (acute renal failure, *P* = 3.7e – 6). Genetic associations at the *UNCX* locus colocalized with renal *UNCX* expression and with circulating levels of several proteins encoded in *trans*, including cystatin C. Associations between the aggregated effect of pLoF variants and continuous markers for kidney function or damage identified the top 1 prioritized genes *PKHD1, SLC22A7*, and *SLC7A9* for creatinine, and *PKHD1* and *SLC7A9* for cystatin C (Supplementary Table S15).

**Figure 6 ∣:**
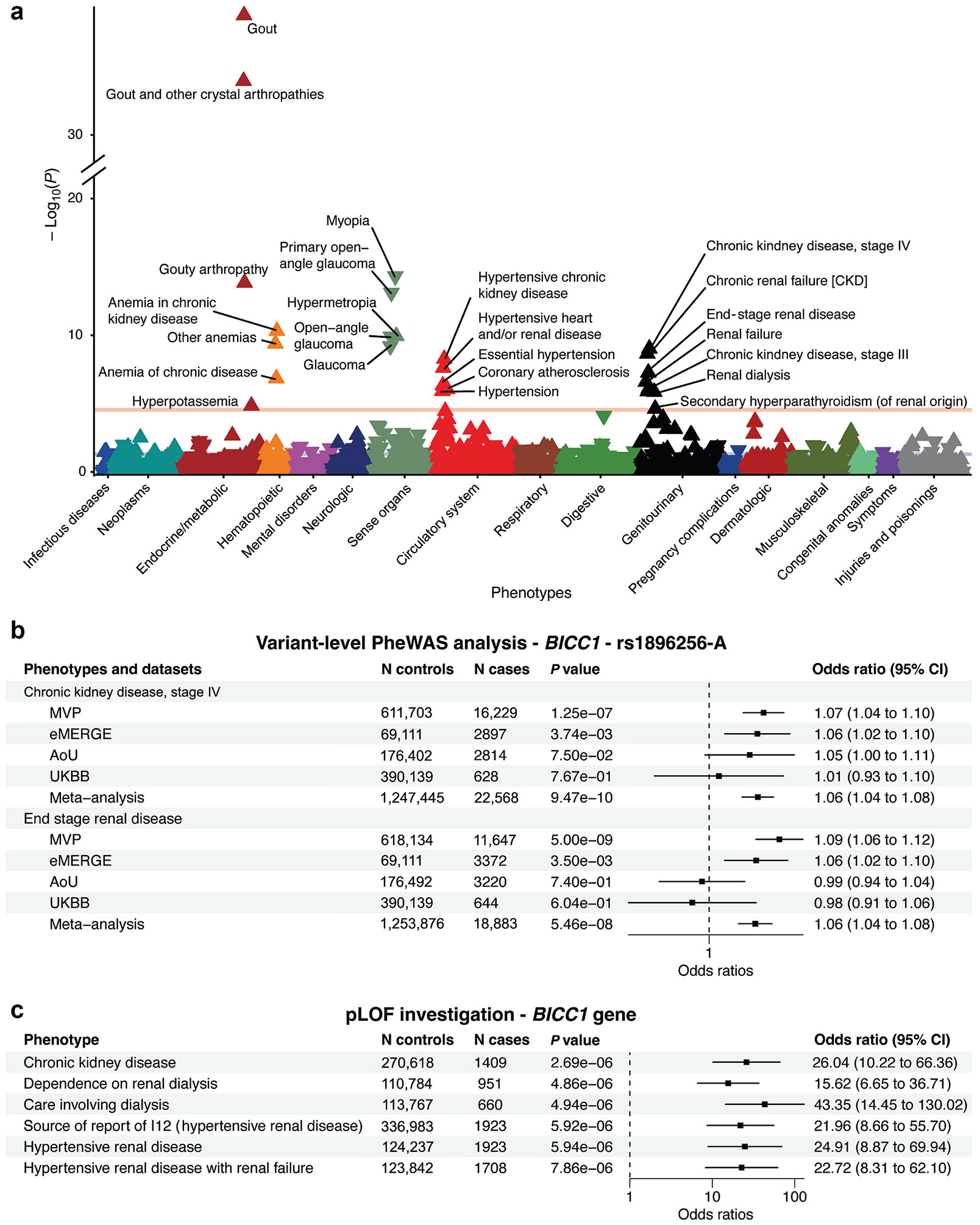
*BICC1* is associated with chronic kidney disease (CKD) risk. (**a**) Manhattan plot from a meta-analysis of phenome-wide association studies (PheWASs) of *BICC1* rs1896256 in several large electronic medical record studies (Methods; the *G* allele is modeled). *y* Axis: −log_10_(association *P*); *x* axis: phenotypes by category. (**b**) Forest plot showing odds ratios and confidence intervals (CIs) for selected phenotypes associated with rs1896256-A in the individual studies. (**c**) Forest plot showing the aggregated effect of putative loss-of-function (pLOF) variants in *BICC1* with kidney-related phenotypes in the UK Biobank. AoU, All of Us; eMERGE, Electronic Medical Records and Genomics; MVP, Million Veteran Program; UKBB, UK Biobank.

*BICC1* was of special interest, as we detected associations with TKV and cortex volume but not with eGFR in the MRI sample (Figure 5b) nor in a 20-fold larger eGFR GWAS meta-analysis^4^ (Supplementary Table S12). Its potential role in CKD is supported by associations we identified with the aggregated effect of rare pLoF variants. A targeted study of the association between genotype at the common *BICC1* variant rs1896246, a proxy of the TKV index variant, with 1817 harmonized *International Classification of Diseases, Tenth Revision*–based clinical codes across 4 large electronic medical record databases (Methods) revealed significant (*P* < 2.75e – 5) associations with increased odds of CKD, kidney failure, dialysis, and gout, and decreased odds of glaucoma and myopia (Figure 6a). The associations with advanced CKD stage 4 and with end-stage kidney failure were supported by several of the evaluated studies (Figure 6b) and further substantiated by highly elevated odds for CKD (OR = 26.0) and dialysis (OR = 15.6) among carriers of pLoF variants in *BICC1* (Figure 6c). Supplementary Table S16 summarizes these and additional disease associations also for the *UNCX* and *UMOD/PDILT* loci. These results are consistent with a potential dual role of *BICC1*, with effects on kidney cysts during development and on kidney structure and CKD in the adult population, and generate a basis for future experimental studies and the targeted evaluation of *BICC1* variants in sequencing data from patients with CKD.

## DISCUSSION

Our study has several principal findings: first, GWAS of kidney subvolumes identify genetic loci missed when only investigating TKV, especially for kidney medulla and sinus. Second, a substantial fraction of kidney volume–associated index variants does not show significant associations with the conventional kidney function marker eGFR, indicating that the genetic architecture of kidney volumes contains information beyond filtration. Third, enrichment analyses revealed that prioritized genes were significantly overrepresented among terms related to kidney development and growth, supporting a role beyond embryonic development. Fourth, we detected a molecular footprint of the identified volume-associated genetic variants on renal gene expression and on circulating protein biomarkers of kidney function at many loci. Fifth, we identified genes with significant associations with kidney volumes but not with eGFR, in which pLoF variants were linked to kidney disease, including *BICC1* and *PKHD1*. Converging evidence from both rare and common variant association in *BICC1* with advanced CKD and kidney failure suggests that the analysis of kidney volumes in a relatively small sample represents a promising approach to identifying CKD risk genes in humans.

A previous, similarly sized GWAS of TKV based on the MR images of the UKB reported the identification of 12 independent signals,^14^ compared with 42 such signals found in our study. The almost perfect correlation of genetic effect sizes between the previous and our study suggests that our identification of many additional loci may be related to different choices related to data analysis and to a different algorithm to perform population-scale segmentation of the kidneys from MRI images.^15^ Our algorithm was developed and validated in the independent NAKO study.^15^ The presence of many genes identified in our study with highly biologically plausible links to the kidney underscores the effectiveness and robustness of transferring a trained convolutional neural network between large datasets.

Only a subset of genes associated with kidney volumes also showed significant associations with eGFR, suggesting that kidney volumes are related to glomerular filtration but also capture additional important properties of the kidney. A previous Mendelian randomization study based on the published genetic associations with TKV^14^ found that lower genetically predicted TKV was associated with higher risk of CKD.^42^ The strong enrichment for terms related to kidney development in our study is consistent with genetic mechanisms that affect kidney development and subsequently kidney volumes among adults. Such mechanisms could affect nephron number,^43^ but also structures such as renal vasculature or adipose or connective tissues. The latter is supported by the observation that 54% of medulla- and 92% of sinus-associated loci were not significantly associated with eGFR and that prioritized genes showed enrichment for angiogenesis and general embryonic development. Reliable data to further test the proposed link between intrauterine growth restriction or prematurity and low nephron number^44^ were unavailable in the UKB. The relevance of sinus volume for kidney function is supported by an inverse relation between sinus fat and GFR and renal vascular resistance.^45^ Additionally, renal biopsies from patients with CKD showed that higher renal sinus fat is linked to worse renal outcomes and systemic hypertension.^46^ Thus, genetic studies of measures of kidney structure, such as kidney subvolumes, may eventually translate into insights of clinical relevance.

Another noteworthy finding emerging from our study is that genetic analyses of kidney volumes in the general population successfully identified genes clearly linked to cyst formation, ciliopathies, and kidney failure in humans and animal models. For instance, rare loss-of-function mutations in *PKHD1* can cause autosomal-recessive polycystic kidney disease.^23^ The kidneys in autosomal-recessive polycystic kidney disease are characterized by enlarged, echogenic kidneys with dilatation of the collecting ducts and early onset of kidney failure. We found that common noncoding variants in the *PKHD1* locus with regulatory potential were associated with TKV and cortex. These findings are consistent with a continuum of genetic effects, in which rare deleterious variants lead to pronounced enlargement of the kidneys and early-onset disease, whereas common variants of smaller effect size are associated with differences in kidney subvolumes among adults. Interestingly, the effect size of the volume-associated index variants on eGFR in our analysis sample was less than half as large and did not even achieve suggestive significance, and the locus also did not show evidence of a shared genetic basis with eGFR using summary statistics from an eGFR GWAS meta-analysis of almost 800,000 individuals.^4^

A second example is *BICC1*, in which we identified a common intronic and a small deletion variant associated with TKV and cortex volume, respectively. The gene encodes for the RNA-binding molecule bicaudal-C, which has an important role in the developing kidney.^47^ Rare deleterious mutations in *BICC1* have been linked to cystic kidney disease in 2 children,^24^ and disruption of *bicc1* in model organisms causes ciliary phenotypes, including kidney cysts.^41,48-50^ Again, there was no colocalization with genetic associations with eGFR in 20-fold larger genetic screens of eGFR,^4^ although the locus was reported in a recent GWAS of eGFR among 282,852 persons of East Asian ancestry^51^ as well as in several GWASs of blood urea nitrogen levels.^4,6,52,53^ In our study, the targeted investigation of kidney volume–associated genes that were also linked to CKD through rare pLoF variants revealed a common variant in *BICC1* that was significantly and concordantly associated with CKD, kidney failure and dialysis, hypertensive CKD, hypertension, and gout. Consistent with a recent report of the locus in association with end-stage renal disease in the Million Veteran Program (MVP) study,^52^ this further establishes *BICC1* as a risk gene for advanced kidney failure in population-based studies and underscores the value of studying kidney volumes. The link of *BICC1* to the Gene Ontology terms “kidney development” and “renal system development” supports its role in kidney morphogenesis and function, which may also underlie its association with hypertension. Similar phenotypes have been observed for other genes causing human ciliopathies, such as *PKD1/2*.

These 2 examples suggest that genetic studies of kidney volumes in the general population may represent a more general approach to gain a deeper understanding of the mechanism underlying cystic kidney disease. Although *PKHD1, BICC1,* and *UMOD* were linked to cortex and TKV, there were also genes linked to human and murine cystic kidney disease, such as *GLIS2* and *SLIT2,* that were only associated with sinus volume, making it unlikely that the presence of cortical cysts can fully explain the observed associations. Along similar lines, most but not all congenital abnormalities of the kidney or urinary tract–related prioritized genes were identified in association with sinus volumes, making it unlikely that developmental abnormalities in the renal pelvis fully account for our findings. Dedicated studies of the presence of cysts as well as their size and volume require special segmentation protocols and are an interesting area of future research.

Strengths of our study include its large sample size, the ability to segment not only TKV but also kidney compartments, and the detailed downstream characterization of identified loci, including comprehensive assessment of molecular and clinical phenotypes. The restriction of the study sample to persons of European ancestry may impact generalizability. However, several of our findings were supported by associations with rare pLoF variants, which should not be subject to potential population stratification, as well as by existing genetically manipulated models. Conventional markers used to calculate kidney function, creatinine, and cystatin C were not measured concomitantly with MR images. However, any time difference between clinical biochemistry and imaging should be nondifferential with respect to genotype, mitigating this concern.

In summary, our study shows that investigations of the genetic architecture of measures of kidney structure, such as kidney subvolumes, provide complementary insights into analyses of kidney function and can successfully highlight genes of relevance in human kidney development and disease.

## Supplementary Material

1

2

3

4

5

6

Supplementary material is available online at www.kidney-international.org.

## Figures and Tables

**Table 1 ∣:** Overview of 25 prioritized genes at kidney volume–associated loci with supporting evidence from human and mouse phenotypes

Gene	Lowest scoring rank	Associated kidney volume(s)	Index variant(s)	Human disease(s)	Mouse phenotype(s)	Association with eGFR (*P* < 1e – 5)
*GATA5*	Top 3	Sinus	rs6142766	NA	Abnormal kidney morphology	True
*MPLKIP*	Top 2	Sinus	rs79841539	NA	Abnormal kidney morphology	True
*TMEM174*	Top 1	Sinus	rs55656316 (cond), 5:72608832_TCTC_T, 5:72608842_CTCT_C (cond)	NA	Abnormal kidney morphology	True
*TMEM60*	Top 1	TKV, cortex, medulla	rs11767651, rs17385641	NA	Abnormal kidney morphology	False
*H1-0*	Top 2	Cortex, sinus	rs71324877, 22:38180407_CAA_C	NA	Abnormal kidney morphology, enlarged kidney	True
*SPRED2*	Top 2	Sinus	2:65581686_GA_G	NA	Abnormal kidney morphology, enlarged kidney	True
*TCF21*	Top 1	Sinus	rs1029212	NA	Abnormal kidney morphology, enlarged kidney	False
*COA6*	Top 3	Sinus	rs271762	NA	Abnormal kidney morphology, increased kidney weight	True
*ADAMTS6*	Top 2	Sinus	rs2163955	NA	Abnormal kidney morphology, small kidney	True
*BICC1*	Top 1	TKV, cortex	rs11593230, 10:60318866_AC_A	NA	Abnormal kidney morphology, small kidney	False
*IL17A*	Top 2	TKV, cortex	rs7449794, rs2580009	NA	Abnormal kidney morphology, small kidney	True
*SSR1*	Top 2	TKV, medulla	rs753277196, rs6925389	NA	Abnormal kidney morphology, small kidney	True
*MLLT3*	Top 1	Sinus	rs1330922	NA	Decreased kidney weight	False
*EBF1*	Top 1	Sinus	rs34155622	NA	Enlarged kidney	False
*COL6A3*	Top 3	TKV, cortex	rs79186933	NA	Enlarged kidney	True
*RND3*	Top 1	Sinus	rs368931559	NA	Polycystic kidney	False
*TIMP4*	Top 1	Medulla, sinus	3:12360884_TG_T, rs9826367 (cond), rs17036160, rs17036160 (cond)	NA	Small kidney	True
*JAG1*	Top 1	Sinus	rs6040079	CAKUT (green; green and amber)	NA	False
*PBX1*	Top 1	Sinus	rs10918068	CAKUT (green; green and amber)	NA	False
*SALL1*	Top 2	TKV, cortex	rs149197133, rs12927956	CAKUT (green; green and amber)	NA	True
*SLIT2*	Top 1	Sinus	rs778833676, rs13113612 (cond)	CAKUT (green and amber)	Abnormal kidney morphology, enlarged kidney	False
*GLIS2*	Top 1	Sinus	rs59945160	Cystic renal disease (green; green and amber)	NA	False
*MKKS*	Top 3	Sinus	rs6040079	Cystic renal disease (green; green and amber)	NA	True
*PKHD1*	Top 1	TKV, cortex	rs7449794, rs2580009	Cystic renal disease (green; green and amber)	NA	False
*UMOD*	Top 2	TKV, cortex, medulla	rs77924615	Cystic renal disease (green; green and amber)	NA	True

CAKUT, congenital abnormalities of the kidney or urinary tract; eGFR, estimated glomerular filtration rate; NA, not applicable; TKV, total kidney volume.

For each gene, the table lists its lowest scoring rank, the associated kidney subvolume(s), the list of index variants (with lead variants from conditional summary statistics indicated as "[cond]"), known associations with human monogenic kidney diseases, mouse renal phenotypes, and whether the index variant shows an independent association with eGFR (*P* < 1e – 5).

## Data Availability

Genome-wide summary statistics of total kidney volume (TKV), cortex, medulla, and sinus volumes are available in the GWAS catalog (https://www.ebi.ac.uk/gwas/, study accessions: GCST90667997 [TKV], GCST90667998 [cortex], GCST90667999 [medulla], and GCST90668000 [sinus]). Access to the genome-wide summary statistics of TKV from Liu *et al.* can be found in the respective publication.^14^ The data used for colocalization analysis (Supplementary Tables S11-S13) are available from the following: Genotype-Tissue Expression (GTEx) V8: https://www.gtexportal.org/home/; Kidney expression quantitative trait loci (eQTL): https://susztaklab.com/Kidney_eQTL/; eQTLGen phase I: https://eqtlgen.org/phase1.html; UK Biobanck PPP: https://www.nature.com/articles/s41586-023-06592-6; Icelanders pGWAS: https://pubmed.ncbi.nlm.nih.gov/34857953/; CKDGen Consortium: urine albumin-creatinine ratio, MA: https://ckdgen.imbi.uni-freiburg.de/datasets/Teumer_2019; urate: https://ckdgen.imbi.uni-freiburg.de/datasets/Tin_2019; and estimated glomerular filtration rate, blood urea nitrogen: https://ckdgen.imbi.uni-freiburg.de/datasets/Wuttke_2019; UKB-TOPMed: https://pheweb.org/UKB-TOPMed/about; and FinnGen release 9: https://r9.finngen.fi/. The dataset of genes associated with abnormal kidney morphology is available from the International Mouse Phenotyping Consortium at https://www.mousephenotype.org/data/phenotypes/MP:0002135. The genes for human congenital abnormalities of the kidney or urinary tract are available from the Genomics England PanelApp at https://panelapp.genomicsengland.co.uk/panels/234. All other relevant data are available from the corresponding author on request. Genome-wide association study annotation pipeline scripts are available at https://github.com/genepi-freiburg/GWASannotation.
