# Supplementary material for: Genetic screens of imaging-derived kidney volumes identify genes linked to kidney function
Source: Kidney Int. Author manuscript; Available in PMC 2026 Jul 5. (PMC13333066; doi:10.1016/j.kint.2025.08.038)
Supplement: 1 [file NIHMS2184648-supplement-1.pdf]

Genetic screens of imaging-derived kidney volumes identify genes linked to kidney function

Table of Contents

Supplementary Methods 2
Image processing and kidney volume assessment 2
Phenotype definition for genetic analyses 3
Heritability estimation 3
Genetic Correlation Analysis 3
Sensitivity Analyses 3
Comparison of GWAS Effect Sizes 4
Conditional analyses 4
GWAS annotation 4
Colocalization 6
GTEx and KPMP enrichment analysis 6
GO, KEGG enrichment analyses 7
Overlap Analysis with Kidney Morphology Data in Mice and Human CAKUT 7
Investigation of significant genes for associations with kidney traits and diseases through analysis of whole-genome-sequencing data 7
Investigation of selected index variants through Phenome-wide association (PheWAS) analysis 8
References 9
Supplementary Results 13
Supplementary Figures 14
Supplementary Figure S1: Distribution of total kidney volume (TKV) (grey), cortex (orange), medulla (yellow), and sinus (blue). 15
Supplementary Figure S2: Phenotypic and genotypic correlation of kidney sub-volumes and eGFR. 16
Supplementary Figure S3: GWAS results for kidney volumes. 17
Supplementary Figure S4: Regional association plots for each of the 34 significant regions identified in TKV GWAS (Methods). See separate file. 18
Supplementary Figure S5: Regional association plots for each of the 24 significant regions identified in cortex GWAS (Methods). See separate file. 18
Supplementary Figure S6: Regional association plots for each of the 26 significant regions identified in medulla GWAS (Methods). See separate file. 18
Supplementary Figure S7: Regional association plots for each of the 71 significant regions identified in sinus GWAS (Methods). See separate file. 18
Supplementary Figure S8: Overview of distinct and shared associated genetic loci across kidney (sub-)volumes 19
Supplementary Figure S9: GWAS results for eGFR. 19
Supplementary Figure S10: Sensitivity analyses after inclusion of eGFR. 20
Supplementary Figure S11: Sensitivity analyses omitting the adjustment of kidney volumes for body surface area (BSA). 21
Supplementary Figure S12: Expression patterns of prioritized genes in loci associated with each kidney volume. 23
Supplementary Figure S13: UpSet plots showing overlap of positive colocalization (PPH4 > 0.8) of significant loci for TKV (a), cortex (b), medulla (c), and sinus (d) volumes with the kidney function markers eGFR, urate, BUN, and UACR. 24

Supplementary Methods

Image processing and kidney volume assessment

We utilized Patchwork,[S1,S2] a hierarchical, multiscale U-net framework, to segment the kidney into compartments and studied the cortex, medulla, and sinus volumes. The convolutional neural network was trained using binary cross-entropy loss, with ground truth established through manual segmentation by experienced radiologists in an iterative procedure. As a starting point, we utilized a previously trained and validated model based on the NAKO Health study.[S3] This model was applied to a random subset of 604 images. While the original NAKO protocol included a separate compartment for cysts and other space-occupying lesions, we adopted a slightly modified strategy in this study. Due to the complex nature of these structures, they were not explicitly segmented and were instead incorporated into the other compartments (cortex, medulla, or sinus) by the model. The results were visually inspected and corrected, were applicable, and subsequently, a final training run was performed. Verification was conducted through manual segmentations performed by two experienced radiologists across twenty cases. To enhance the model's accuracy, the final thresholds for the segmentations were slightly adjusted compared to the original NAKO model to minimize the average volume differences between the model predictions, the manual segmentations and the NAKO statistics. The final model was then applied to all cases, and compartmental volumes were calculated in milliliters (mL) by extracting the full kidneys from probability maps, and selecting the two largest connected segments as the left and right kidneys. In line with previous studies[4], total kidney volume (TKV) was defined as the combined cortex and medulla volumes of both kidneys. After exclusion of incomplete or corrupt imaging data, valid kidney volume measurements were obtained for 48,089 participants.

Individuals flagged during quality control of the genotyping array data were then excluded based on missing heterozygosity, sex chromosome aneuploidy, and genetic kinship information as described previously,[S5] resulting in 43,224 remaining participants, and limited to 42,140 individuals of European Ancestry.[S6] For participants with repeat MRIs, kidney volumes were calculated from their first MRI visit for each participant (instance 2 or 3; **Supplementary Table S1**).

Phenotype definition for genetic analyses

Body surface area (BSA) was calculated from weight and height measurements collected at the time of the MRI visit, as part of the standardized imaging protocol in UK Biobank. BSA values were available for 40,807 of the participants, using the Mosteller formula and expressed as ml/m^2 (**Supplementary Table S1**). Prior to GWAS, BSA-normalized volumes were then inversely transformed, to enable comparison of genetic effect sizes across traits. Lastly, the dataset was restricted to participants whose estimated glomerular filtration rate (eGFR) could be calculated from serum creatinine levels using the CKD-EPI formula,[S7,S8] resulting in a final sample size of 38,816 participants.

Heritability estimation

A genetic relationship matrix was calculated from all autosomal SNPs with minor allele frequency (MAF) of >0.01 using GCTA-GRM.[S9] Kidney volume residuals were adjusted for principal components (PC1-PC10), sex, age at MRI, age squared, and assessment center. GCTA-GREML[S10] was then used to estimate the proportion of variation in these residuals explained by these SNPs.

Genetic Correlation Analysis

We estimated the pairwise genetic correlation (r_g) between TKV and sub-volumes (cortex, medulla, and sinus) using Linkage Disequilibrium Score Regression (LDSC v1.0.1).[S11] GWAS summary statistics for each trait were filtered to include only variants present in the HapMap3 reference panel. Precomputed linkage disequilibrium (LD) scores from the 1000 Genomes Project European (EUR) population were used, as recommended. We extracted the LD score regression intercept for each trait, which quantifies the contribution of confounding factors, such as population stratification or cryptic relatedness, to the inflation of test statistics.

Sensitivity Analyses

We conducted a sensitivity analysis using two additional GWAS models to evaluate the robustness of the associations identified in our primary GWAS model (Model 1, above). To assess whether adjustment for eGFR attenuated the associations between genetic variants and kidney volumes, Model 2 included additional adjustment for eGFR. To evaluate the

potential influence of normalizing kidney volumes to body surface in Model 1, Model 3 omitted the BSA normalization step of the kidney volumes, instead using raw volume data that were inverse normal transformed prior to analysis.

Comparison of GWAS Effect Sizes

To compare the effect sizes between two GWAS, we calculated Z-statistics for each SNP of interest using the formula:

$$Z = \frac{\beta_1 - \beta_2}{\sqrt{SE_1^2 + SE_2^2}}$$

where β1 and β2 represent the effect sizes from the two GWAS, and SE1 and SE2 are the corresponding standard errors. The comparison P-values were derived from these Z-statistics using a two-tailed normal distribution.

Conditional analyses

Independent association signals within these loci were identified based on approximate conditional analyses via the GCTA COJO-Slct algorithm,[S9] with default parameters (*P*<5e-8 and a collinearity of 0.9). For each conditionally independent SNP, conditional summary statistics were computed by conditioning on all other independent SNPs in the gene region using the GCTA COJO-Cond algorithm with default parameters (collinearity of 0.9).[S9]

GWAS annotation

The GWAS annotation pipeline used the following sources of evidence:

<u>Nearest Gene Identification</u>: index variants were defined as the most statistically significant variants in the GWAS in each defined region. For each index variant, genes within a ±1 Mb window were identified using the **GenomicRanges** R package.[S12] The nearest genes were ranked by their proximity to the variant, based on the gene start and end position. Only protein-coding genes were considered.

<u>LD Overlapping Genes</u>: proxy variants were identified using PLINK (v1.90)[S13] by calculating LD between the index variants and other variants in the dataset, with a window size of 1 Mb and an r^2 threshold of 0.8. Proxy variants with r^2 >0.8 were selected for further analysis. LD regions were defined by merging the regions of proxy variants with those of the sentinel variants,

extending their coordinates as needed. The LD range for each index variant was defined as the span between the left- and right-most proxy variants (±5 kb). Genes overlapping these LD regions were then extracted and annotated using Ensembl gene models (GRCh38). In cases where the index variant had no proxies, the coordinates of the index variant were taken as the LD range.

eQTL association: significant variant-gene pair associated with *cis*-eGenes from GTEx v8[S14] were used for all available tissues. Index and linked proxy variants were checked for *cis*-eQTL associations, and unique gene targets were retained. Gene annotations were retrieved using Ensembl 111 gene models (GRCh38).[S15]

Colocalization with eQTL and pQTL: colocalization analysis was performed to assess the posterior probability of the same variant driving the association with kidney volume and gene expression or protein levels in a given region (see below). *Cis*-eQTL datasets included all available tissues eQTLs from GTEx v8,[S14] eQTLGen[S16] and Kidney eQTL.[S17] GTEx *cis*-eQTL colocalization results were specifically scrutinized for available tissues of interest, namely kidney cortex, liver, and whole blood additionally to Kidney eQTL. For colocalization with *cis*-pQTLs, we used plasma pQTL datasets from UKB[S18] and Icelanders.[S19] Colocalization results were filtered to retain only those with a high posterior probability of a shared underlying variant (PPH4 >0.8) and significant *cis*-eQTL or *cis*-pQTL associations (P<5e-8).

Variants with high VEP impact: functional consequences of proxy variants were assessed using the Ensembl 101 Variant Effect Predictor (VEP).[S20] Variants with MODERATE or HIGH impact were considered.

Scoring and Causal Gene Selection: a composite score was calculated for each gene based on several factors: nearest gene information, with scores of 1.00, 0.99 and 0.98 for the first, second and third nearest gene, respectively; LD overlapping gene: 1.00; variants with high VEP impact: 1.02; eQTL association: 0.75; colocalization with eQTL (tissues of interest): 1.04; colocalization with eQTL (general): 1.03, colocalization with pQTL: 1.04. The final score for eQTL colocalization was applied only once: if colocalization with tissues of interest was detected, the highest score (1.04) was assigned; otherwise, if a more general colocalization with eQTLs was observed, a score of 1.03 was given. In the absence of colocalization, the gene's eQTL association score (0.75) was applied. Lastly, for each independent index variant, the top three genes (Top1, Top2, and Top3) were prioritized based on their final scores. The

differences in the second decimal between scores were introduced to facilitate the discrimination between the top three genes for each independent index variant.

Colocalization

Genetic colocalization analysis was performed utilizing the coloc.abf function in the coloc R package[21] to examine whether genetic associations with two traits were likely caused by the same underlying variant. Colocalization was only conducted when both traits showed at least one variant with *P*<5e-8 in the region of interest. The results were then filtered to include only those with a posterior probability of H4 (PPH4)>0.8, indicating a shared causal variant. Colocalization analyses were performed among the kidney sub-volumes, as well as for each kidney sub-volume with molecular traits (eQTL summary statistics from GTEx v8,[S14] eQTLGen,[S16] and Kidney eQTL,[S17] plasma pQTL summary statistics from UKB[S18] and Icelanders[S19]), with established kidney function traits from the CKDGen Consortium,[S22] including eGFR,[S23] urate,[S24] blood urea nitrogen (BUN)[S23] and urine albumin-creatinine ratio (uACR),[S25] and with clinical traits or diseases from UKB TOPMed[S26] and FinnGen.[S27]

Marginal summary statistics were used for all colocalization analyses to maintain a uniform workflow across all traits, because colocalization was performed not only among the kidney sub-volumes but also across hundreds of publicly available datasets for which individual-level data were not accessible. Among kidney sub-volumes, the potential of false negative colocalizations was considered low: since cortex and medulla volumes are part of TKV, a common genetic architecture of shared association signals is expected, rather than distinct causal genes mapping into immediate physical proximity by chance.

GTEx and KPMP enrichment analysis

Tissue and cell type enrichment analyses were conducted using lists with the top 1 prioritized genes as the input, separately for TKV, cortex, medulla, and sinus. Using GTEx v8[S14] with reassigned kidney and medulla samples as described previously[S28] as well as KPMP kidney single-cell and single-nucleus RNA-seq data,[S29] the top 10% of highly expressed genes for each GTEx tissue and KPMP cell type were identified.[S30] A database was created using all Entrez gene identifiers based on the R package org.Hs.eg.db version 3.12.0. We stored gene length, the number of independent SNPs per gene,[S31] and whether a gene was among the top 10% highly expressed genes in a given tissue or cell type. To assess whether genes of interest were

significantly overrepresented, we performed 100 million random draws, selecting the same number of genes as in each input gene list while matching the background genes for deciles of independent SNP counts and gene length. These draws were used to compare the overlaps with tissues or cell types identified for the original gene list, yielding empirical p-values. Nominal p-values were then adjusted for multiple testing using the Benjamini-Hochberg procedure,[S32] for GTEx and KPMP data separately.

GO, KEGG enrichment analyses

Enrichment testing of the Top 1 prioritized genes with our GWAS annotation pipeline for TKV (34 genes), cortex (24 genes), medulla (26 genes) and sinus (71 genes) was performed using Gene Ontology (GO) terms and Kyoto Encyclopedia of Genes and Genomes (KEGG) pathways.[S33] For all enrichment analyses, we used hypergeometric tests implemented in the R package clusterProfiler version 4.0.5,[S34] using the genes available in the respective resource as background genes (GO-BP: 18888; GO-MF: 18522; GO-CC: 19894 ; KEGG: 8878). *P*-values were corrected for multiple testing using the Benjamini-Hochberg procedure[S32] separately in each resource. All results were filtered for terms with at least two genes and adjusted $P<0.05$.

Overlap Analysis with Kidney Morphology Data in Mice and Human CAKUT

We analyzed whether the top three prioritized genes per association signal overlapped with genes associated with abnormal kidney morphology in mice from the International Mouse Phenotyping Consortium (IMPC),[S35] with genes known to harbor causative mutations for human congenital anomalies of the kidney and urinary tract (CAKUT), and with genes known to harbor causative mutations for cystic renal disease. The phenotype categories for the mouse analysis included "abnormal kidney morphology," "enlarged kidney," "decreased kidney weight," "small kidney," "increased kidney weight," "polycystic kidney," and "single kidney." For human CAKUT, we used "green and amber" genes from the CAKUT (Version 1.178) panel available on the Genomics England PanelApp as of 29 November 2024[36]. For human cystic renal disease, we used "green and amber" genes from the cystic renal disease (Version 12.3) panel available on the Genomics England PanelApp as of 27 May 2025.[S36] Human-to-mouse orthologs were identified using the file, downloaded from the Human and Mouse Orthology Prediction (HCOP) tool[S37] at https://www.genenames.org/tools/hcop/.

Investigation of significant genes for associations with kidney traits and diseases through analysis of whole-genome-sequencing data

Queries of the association between the aggregate effect of rare, putative loss-of-function variants in each gene with kidney traits and diseases were based on analysis of whole-genome sequencing data from the EUR subset of 484,111 UKB participants, as available through the AstraZeneca PheWAS Portal.[S38] We limited our queries to gene-level associations to directly link them with the top 3 genes identified in our GWAS annotation pipeline for TKV, cortex, medulla, and sinus. Collapsing analyses aggregated all rare, putatively deleterious, qualifying variants in each gene that met pre-specified criteria, including nine dominant and one recessive model, and testing them against a given phenotype. *P*-values for binary traits associations were determined by Fisher's exact two-sided test and *P*-values for quantitative traits were determined by linear regression corrected for age, sex, and their interaction (age×sex). Associated genes with P<1e-5 in any of the models were kept, and further filtered to only retain binary traits with ≥50 cases and controls and continuous traits with more than 30 observations. Additionally, the number of cases and controls with qualifying variants for binary phenotypes or the number of participants with qualifying variants for quantitative phenotypes had to be ≥3.

Investigation of selected index variants through Phenome-wide association (PheWAS) analysis

Comprehensive meta-PheWAS analysis was conducted by integrating electronic health record (EHR) data from the eMERGE-III,[S39] All of Us (AoU),[S40] UK Biobank (UKBB),[S5] and Million Veteran Program (MVP)[S41] datasets for each independent genome-wide significant locus. The eMERGE-III dataset included EHR-linked GWAS data for 102,138 individuals, while the AoU dataset comprised 312,944 individuals. The UKBB dataset consisted of 488,377 individuals with a similar EHR-linked GWAS setup. The MVP PheWAS data were utilized from a recent publication, with a total sample size of N=635,969.[S42] To ensure harmonized meta-analysis, we standardized phenotype data by converting all ICD-10 codes to the ICD-9-CM system. This conversion revealed 20,783 unique ICD-9 codes for eMERGE participants, 12,945 for AoU, and 10,221 for UKBB participants. These codes were then mapped to 1817 unique phecodes, followed by logistic regression analyses.[S43] Our regression models adjusted for covariates, including age, sex, study site, imputation batch, and five principal components representing

ancestry information. We then performed a fixed-effects meta-analysis of the study-specific PheWAS from eMERGE-III, AoU UKBB, and MVP datasets using the PheWAS R package.[S44] To determine phenome-wide significance, we applied a stringent Bonferroni correction, setting the significance threshold at P<2.75e-5 to account for multiple testing across the 1817 independent phecodes investigated.

References

S1. Reisert M, Russe M, Elsheikh S, Kellner E, Skibbe H. Deep Neural Patchworks: Coping with Large Segmentation Tasks. Published online June 7, 2022. doi:10.48550/arXiv.2206.03210

S2. Çiçek Ö, Abdulkadir A, Lienkamp SS, Brox T, Ronneberger O. 3D U-Net: Learning Dense Volumetric Segmentation from Sparse Annotation. In: Ourselin S, Joskowicz L, Sabuncu MR, Unal G, Wells W, eds. *Medical Image Computing and Computer-Assisted Intervention – MICCAI 2016*. Springer International Publishing; 2016:424-432. doi:10.1007/978-3-319-46723-8_49

S3. Kellner E, Sekula P, Lipovsek J, et al. Imaging Markers Derived From MRI-Based Automated Kidney Segmentation. *Dtsch Arztebl Int*. 2024;121(9):284-290. doi:10.3238/arztebl.m2024.0040

S4. Roseman DA, Hwang SJ, Oyama-Manabe N, et al. Clinical associations of total kidney volume: the Framingham Heart Study. *Nephrol Dial Transplant*. 2017;32(8):1344-1350. doi:10.1093/ndt/gfw237

S5. Bycroft C, Freeman C, Petkova D, et al. The UK Biobank resource with deep phenotyping and genomic data. *Nature*. 2018;562(7726):203-209. doi:10.1038/s41586-018-0579-z

S6. Karczewski KJ, Gupta R, Kanai M, et al. Pan-UK Biobank GWAS improves discovery, analysis of genetic architecture, and resolution into ancestry-enriched effects. Published online October 1, 2024:2024.03.13.24303864. doi:10.1101/2024.03.13.24303864

S7. Levey AS, Stevens LA, Schmid CH, et al. A new equation to estimate glomerular filtration rate. *Ann Intern Med*. 2009;150(9):604-612. doi:10.7326/0003-4819-150-9-200905050-00006

S8. Pattaro C, Riegler P, Stifter G, Modenese M, Minelli C, Pramstaller PP. Estimating the glomerular filtration rate in the general population using different equations: effects on classification and association. *Nephron Clin Pract*. 2013;123(1-2):102-111. doi:10.1159/000351043

S9. Yang J, Lee SH, Goddard ME, Visscher PM. GCTA: a tool for genome-wide complex trait analysis. *Am J Hum Genet*. 2011;88(1):76-82. doi:10.1016/j.ajhg.2010.11.011

S10. Yang J, Benyamin B, McEvoy BP, et al. Common SNPs explain a large proportion of the heritability for human height. *Nat Genet*. 2010;42(7):565-569. doi:10.1038/ng.608

S11. Bulik-Sullivan BK, Loh PR, Finucane HK, et al. LD Score regression distinguishes confounding from polygenicity in genome-wide association studies. *Nat Genet*. 2015;47(3):291-295. doi:10.1038/ng.3211

S12. Lawrence M, Huber W, Pagès H, et al. Software for computing and annotating genomic ranges. *PLoS Comput Biol*. 2013;9(8):e1003118. doi:10.1371/journal.pcbi.1003118

S13. Chang CC, Chow CC, Tellier LC, Vattikuti S, Purcell SM, Lee JJ. Second-generation PLINK: rising to the challenge of larger and richer datasets. *Gigascience*. 2015;4:7. doi:10.1186/s13742-015-0047-8

S14. Consortium Gte. The GTEx Consortium atlas of genetic regulatory effects across human tissues. *Science*. 2020;369(6509):1318-1330. doi:10.1126/science.aaz1776

S15. Dyer SC, Austine-Orimoloye O, Azov AG, et al. Ensembl 2025. *Nucleic Acids Res*. 2025;53(D1):D948-D957. doi:10.1093/nar/gkae1071

S16. Võsa U, Claringbould A, Westra HJ, et al. Large-scale cis- and trans-eQTL analyses identify thousands of genetic loci and polygenic scores that regulate blood gene expression. *Nat Genet*. 2021;53(9):1300-1310. doi:10.1038/s41588-021-00913-z

S17. Liu H, Doke T, Guo D, et al. Epigenomic and transcriptomic analyses define core cell types, genes and targetable mechanisms for kidney disease. *Nat Genet*. 2022;54(7):950-962. doi:10.1038/s41588-022-01097-w

S18. Sun BB, Chiou J, Traylor M, et al. Plasma proteomic associations with genetics and health in the UK Biobank. *Nature*. 2023;622(7982):329-338. doi:10.1038/s41586-023-06592-6

S19. Ferkingstad E, Sulem P, Atlason BA, et al. Large-scale integration of the plasma proteome with genetics and disease. *Nat Genet*. 2021;53(12):1712-1721. doi:10.1038/s41588-021-00978-w

S20. McLaren W, Gil L, Hunt SE, et al. The Ensembl Variant Effect Predictor. *Genome Biol*. 2016;17(1):122. doi:10.1186/s13059-016-0974-4

S21. Giambartolomei C, Vukcevic D, Schadt EE, et al. Bayesian test for colocalisation between pairs of genetic association studies using summary statistics. *PLoS Genet*. 2014;10(5):e1004383. doi:10.1371/journal.pgen.1004383

S22. Köttgen A, Pattaro C. The CKDGen Consortium: ten years of insights into the genetic basis of kidney function. *Kidney Int*. 2020;97(2):236-242. doi:10.1016/j.kint.2019.10.027

S23. Wuttke M, Li Y, Li M, et al. A catalog of genetic loci associated with kidney function from analyses of a million individuals. *Nat Genet*. 2019;51(6):957-972. doi:10.1038/s41588-019-0407-x

S24. Tin A, Marten J, Halperin Kuhns VL, et al. Target genes, variants, tissues and transcriptional pathways influencing human serum urate levels. *Nat Genet*. 2019;51(10):1459-1474. doi:10.1038/s41588-019-0504-x

S25. Teumer A, Li Y, Ghasemi S, et al. Genome-wide association meta-analyses and fine-mapping elucidate pathways influencing albuminuria. *Nat Commun*. 2019;10(1):4130. doi:10.1038/s41467-019-11576-0

S26. Taliun D, Harris DN, Kessler MD, et al. Sequencing of 53,831 diverse genomes from the NHLBI TOPMed Program. *Nature*. 2021;590(7845):290-299. doi:10.1038/s41586-021-03205-y

S27. Kurki MI, Karjalainen J, Palta P, et al. FinnGen provides genetic insights from a well-phenotyped isolated population. *Nature*. 2023;613(7944):508-518. doi:10.1038/s41586-022-05473-8

S28. Haug S, Muthusamy S, Li Y, et al. Multi-omic analysis of human kidney tissue identified medulla-specific gene expression patterns. *Kidney Int*. 2024;105(2):293-311. doi:10.1016/j.kint.2023.10.024

S29. Lake BB, Menon R, Winfree S, et al. An atlas of healthy and injured cell states and niches in the human kidney. *Nature*. 2023;619(7970):585-594. doi:10.1038/s41586-023-05769-3

S30. Finucane HK, Reshef YA, Anttila V, et al. Heritability enrichment of specifically expressed genes identifies disease-relevant tissues and cell types. *Nat Genet*. 2018;50(4):621-629. doi:10.1038/s41588-018-0081-4

S31. Schlosser P, Scherer N, Grundner-Culemann F, et al. Genetic studies of paired metabolomes reveal enzymatic and transport processes at the interface of plasma and urine. *Nat Genet*. 2023;55(6):995-1008. doi:10.1038/s41588-023-01409-8

S32. Benjamini Y, Hochberg Y. Controlling the False Discovery Rate: A Practical and Powerful Approach to Multiple Testing. *Journal of the Royal Statistical Society: Series B (Methodological)*. 1995;57(1):289-300. doi:10.1111/j.2517-6161.1995.tb02031.x

S33. Kanehisa M, Goto S. KEGG: kyoto encyclopedia of genes and genomes. *Nucleic Acids Res*. 2000;28(1):27-30. doi:10.1093/nar/28.1.27

S34. Wu T, Hu E, Xu S, et al. clusterProfiler 4.0: A universal enrichment tool for interpreting omics data. *Innovation (Camb)*. 2021;2(3):100141. doi:10.1016/j.xinn.2021.100141

S35. Groza T, Gomez FL, Mashhadi HH, et al. The International Mouse Phenotyping Consortium: comprehensive knockout phenotyping underpinning the study of human disease. *Nucleic Acids Res*. 2023;51(D1):D1038-D1045. doi:10.1093/nar/gkac972

S36. Martin AR, Williams E, Foulger RE, et al. PanelApp crowdsources expert knowledge to establish consensus diagnostic gene panels. *Nat Genet*. 2019;51(11):1560-1565. doi:10.1038/s41588-019-0528-2

S37. Yates B, Gray KA, Jones TEM, Bruford EA. Updates to HCOP: the HGNC comparison of orthology predictions tool. *Brief Bioinform*. 2021;22(6):bbab155. doi:10.1093/bib/bbab155

S38. Wang Q, Dhindsa RS, Carss K, et al. Rare variant contribution to human disease in 281,104 UK Biobank exomes. *Nature*. 2021;597(7877):527-532. doi:10.1038/s41586-021-03855-y

S39. Khan A, Shang N, Petukhova L, et al. Medical Records-Based Genetic Studies of the Complement System. *J Am Soc Nephrol*. 2021;32(8):2031-2047. doi:10.1681/ASN.2020091371

S40. All of Us Research Program Genomics Investigators. Genomic data in the All of Us Research Program. *Nature*. 2024;627(8003):340-346. doi:10.1038/s41586-023-06957-x

S41. Verma A, Huffman JE, Rodriguez A, et al. Diversity and scale: Genetic architecture of 2068 traits in the VA Million Veteran Program. *Science*. 2024;385(6706):eadj1182. doi:10.1126/science.adj1182

S42. Liu H, Abedini A, Ha E, et al. Kidney multiome-based genetic scorecard reveals convergent coding and regulatory variants. *Science*. 2025;387(6734):eadp4753. doi:10.1126/science.adp4753

S43. Denny JC, Ritchie MD, Basford MA, et al. PheWAS: demonstrating the feasibility of a phenome-wide scan to discover gene-disease associations. *Bioinformatics*. 2010;26(9):1205-1210. doi:10.1093/bioinformatics/btq126

S44. Carroll RJ, Bastarache L, Denny JC. R PheWAS: data analysis and plotting tools for phenome-wide association studies in the R environment. *Bioinformatics*. 2014;30(16):2375-2376. doi:10.1093/bioinformatics/btu197

Supplementary Results

In sensitivity analyses that additionally adjusted for eGFR, 15 of 34 TKV-associated index SNPs remained significant (P<5e-8), nine out of 24 cortex-associated index SNPs, and 14 out of 26 medulla-associated index SNPs, highlighting SNPs that carry information about kidney structure above and beyond filtration (**Supplementary Table S7**). Sinus volume associations remained hardly unchanged, with 69 out of 71 index SNPs still showing genome-wide significant, eGFR-adjusted associations. The Pearson correlation coefficients between the genetic effect sizes with and without eGFR adjustment were >0.9 for each kidney sub-volume (**Supplementary Figure S10**, **Supplementary Table S7**). A second sensitivity analysis without BSA-adjustment showed that some of the BSA-adjusted index SNPs from the main analysis no longer reached genome-wide significance, but SNP effect sizes remained highly correlated (Pearson correlation≥0.99 for all volumes; **Supplementary Figure S11**, **Supplementary Table S7**). Additional sensitivity analyses using height-normalized volumes, as well as with BSA adjustment instead of normalization, also yielded very similar results (data not shown).

Supplementary Figures

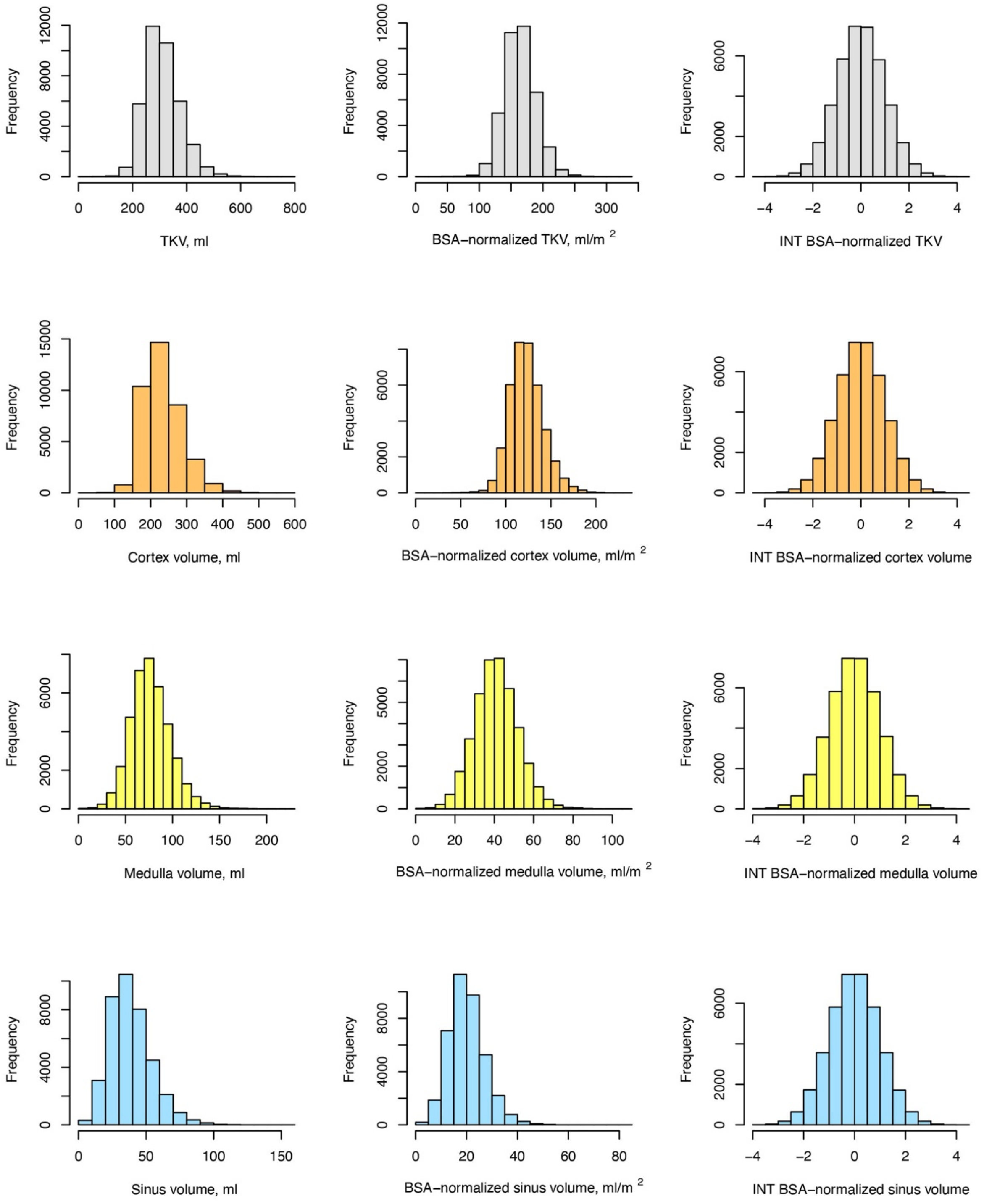

Supplementary Figure S1: Distribution of total kidney volume (TKV) (grey), cortex (orange), medulla (yellow), and sinus (blue). For each volume, the left histogram displays the raw volume measurements (ml), the middle histogram shows the volume corrected for Body Surface Area (BSA) (ml/m²), and the right histogram presents the inverse normal transformed corrected volume. BSA: body surface area; INT: inverse normal transformed.

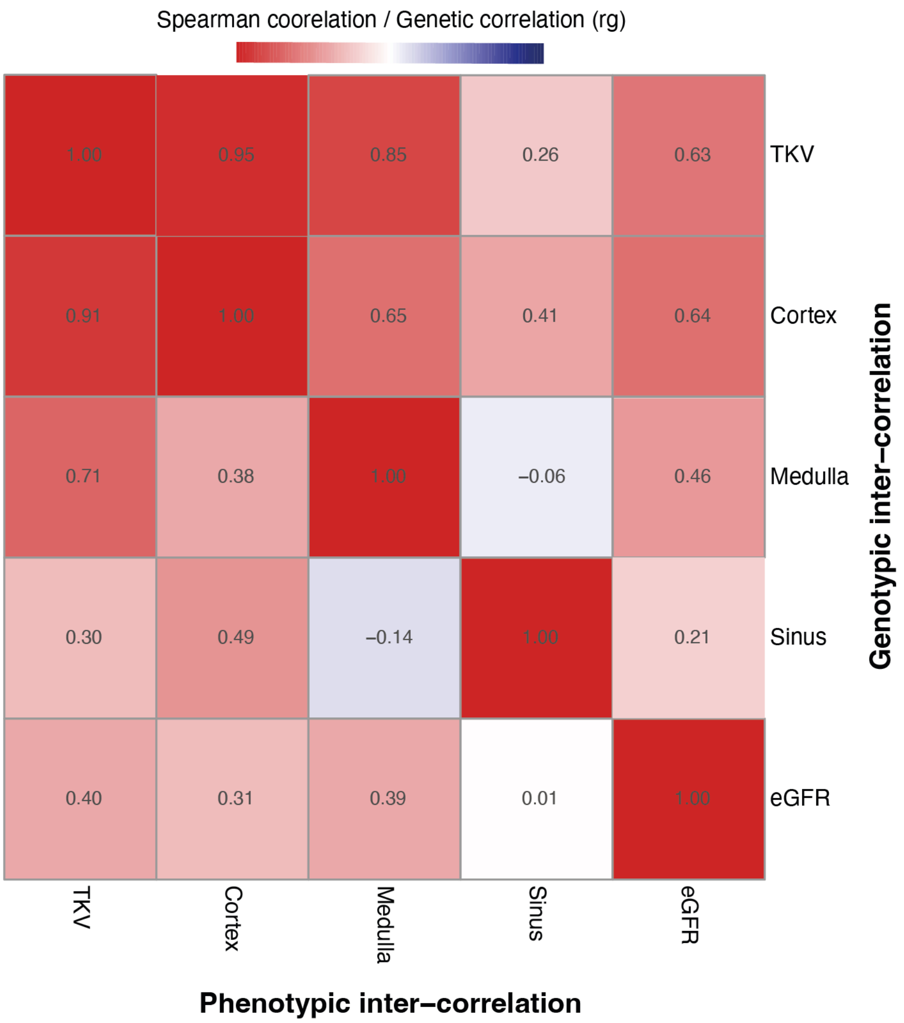

Supplementary Figure S2: Phenotypic and genotypic correlation of kidney sub-volumes and eGFR. The heatmap displays pairwise correlations between total kidney volume (TKV), cortex, medulla, sinus, and eGFR. Lower triangle shows phenotypic inter-correlations (Spearman's ρ), and the upper triangle shows genetic correlations (r_g). Warmer colors indicate stronger positive correlations, while cooler colors represent negative correlations.

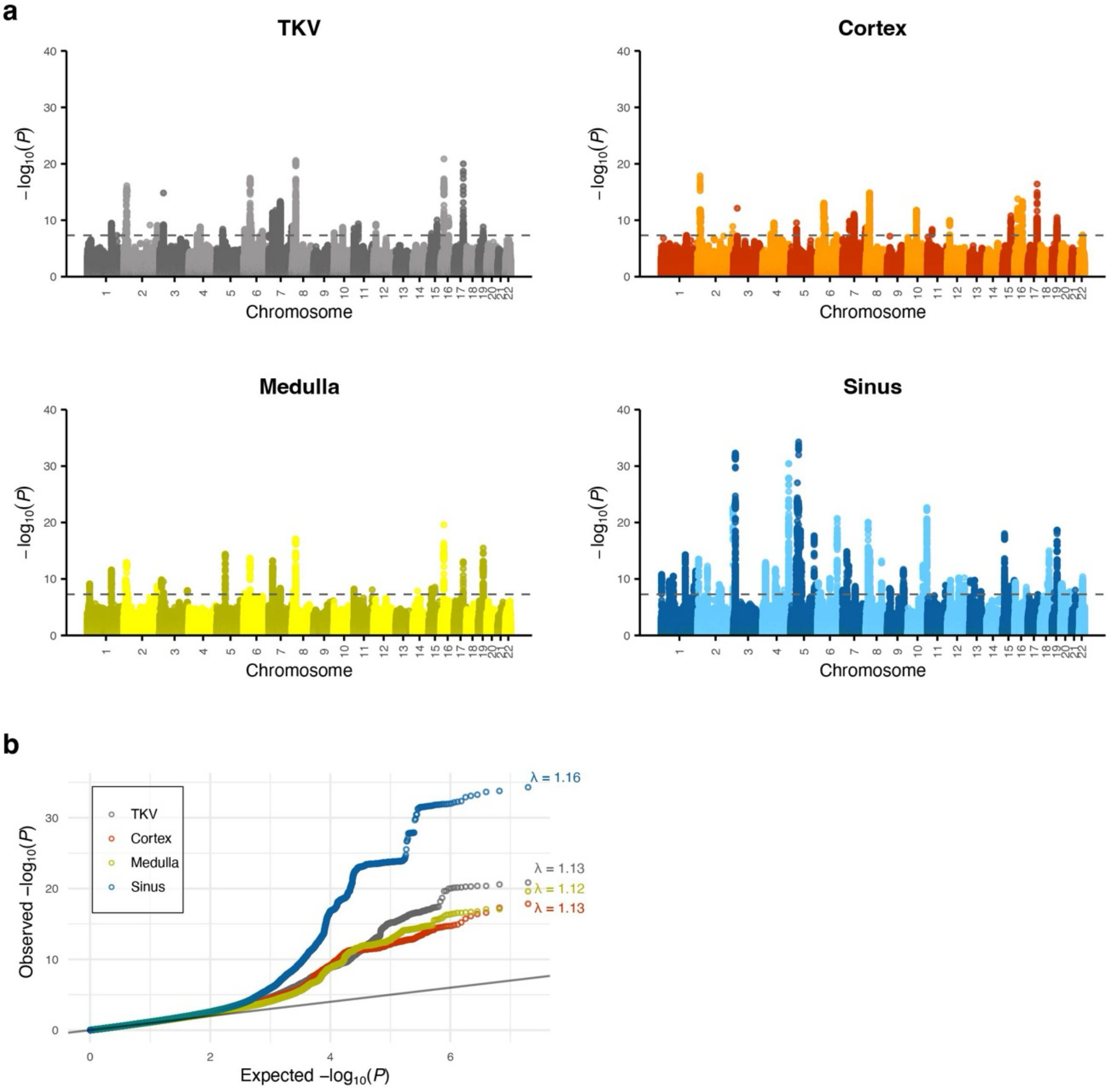

Supplementary Figure S3: GWAS results for kidney volumes. (a) Manhattan plots for total kidney volume (TKV) (grey), cortex (orange), medulla (yellow), and sinus (blue). The dashed lines indicate the genome-wide significance threshold (5e-8). (b) Quantile-quantile (QQ) plots identify and corresponding inflation (λ) for volume (TKV) (grey), cortex (orange), medulla (yellow), and sinus (blue). Volumes were BSA-normalized and inverse-normal transformed. Intercepts from LD score regression of 1.01 each for TKV, cortex, and medulla volumes, as well as of 1.05 for sinus volumes, indicate that confounding factors like population stratification and cryptic relatedness are unlikely to have caused the observed, slight inflation in test statistics (l values).

Supplementary Figure S4: Regional association plots for each of the 34 significant regions identified in TKV GWAS (Methods). The region is included above each plot. SNPs are plotted by position (b38) for the defined window versus -log10 (association P-values) from marginal TKV P-values. For regions for which there were identified more than one independent SNPs, the following plots were created sequentially: marginal statistics, marginal statistics depicting the independent variants in distinct colors, and conditional statistics. The purple diamond highlights the most significant SNP for each association. SNPs are color-coded to reflect their linkage disequilibrium (LD) with this SNP. Genes, exons and the direction of transcription from the University of California at Santa Cruz genome browser are depicted. Plots were generated using Locus Zoom (Pruim et al. 2010). **See separate file.**

Supplementary Figure S5: Regional association plots for each of the 24 significant regions identified in cortex GWAS (Methods). The region is included above each plot. SNPs are plotted by position (b38) for the defined window versus -log10 (association P-values) from marginal cortex P-values. For regions for which there were identified more than one independent SNPs, the following plots were created sequentially: marginal statistics, marginal statistics depicting the independent variants in distinct colors, and conditional statistics. The purple diamond highlights the most significant SNP for each association. SNPs are color-coded to reflect their linkage disequilibrium (LD) with this SNP. Genes, exons and the direction of transcription from the University of California at Santa Cruz genome browser are depicted. Plots were generated using Locus Zoom (Pruim et al. 2010). **See separate file.**

Supplementary Figure S6: Regional association plots for each of the 26 significant regions identified in medulla GWAS (Methods). The region is included above each plot. SNPs are plotted by position (b38) for the defined window versus -log10 (association P-values) from marginal medulla P-values. For regions for which there were identified more than one independent SNPs, the following plots were created sequentially: marginal statistics, marginal statistics depicting the independent variants in distinct colors, and conditional statistics. The purple diamond highlights the most significant SNP for each association. SNPs are color-coded to reflect their linkage disequilibrium (LD) with this SNP. Genes, exons and the direction of transcription from the University of California at Santa Cruz genome browser are depicted. Plots were generated using Locus Zoom (Pruim et al. 2010). **See separate file.**

Supplementary Figure S7: Regional association plots for each of the 71 significant regions identified in sinus GWAS (Methods). The region is included above each plot. SNPs are plotted by position (b38) for the defined window versus -log10 (association P-values) from marginal sinus P-values. For regions for which there were identified more than one independent SNPs, the following plots were created sequentially: marginal statistics, marginal statistics depicting the independent variants in distinct colors, and conditional statistics. The purple diamond highlights the most significant SNP for each association. SNPs are color-coded to reflect their linkage disequilibrium (LD) with this SNP. Genes, exons and the direction of transcription from the University of California at Santa Cruz genome browser are depicted. Plots were generated using Locus Zoom (Pruim et al. 2010). **See separate file.**

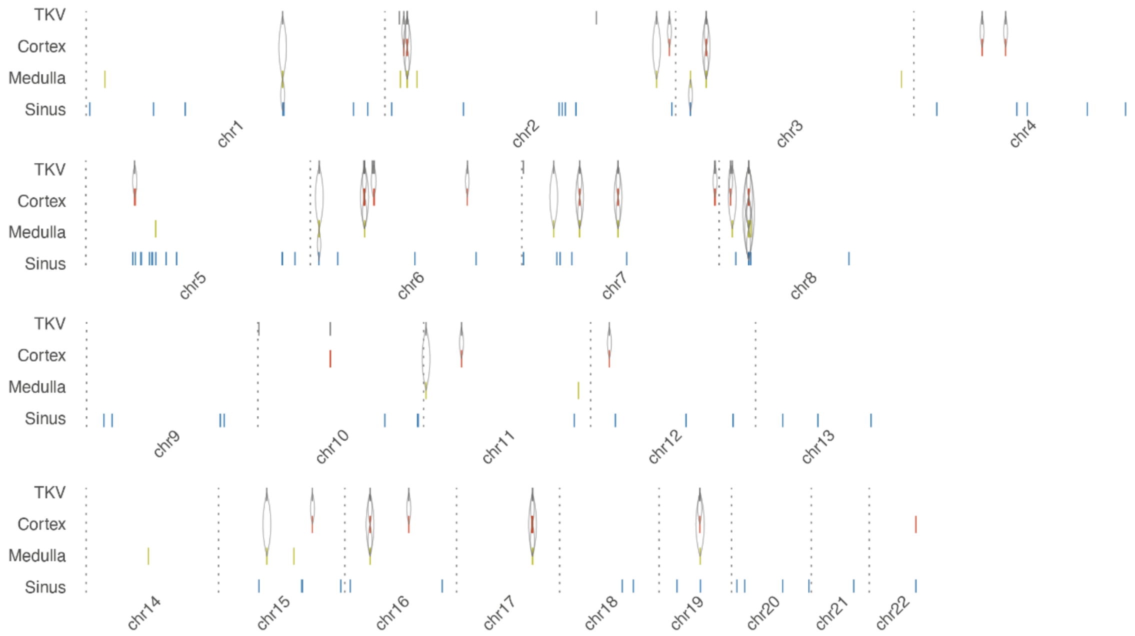

Supplementary Figure S8: Overview of distinct and shared associated genetic loci across kidney (sub-)volumes. Significant loci for each (sub-)volume are plotted along chromosomes 1–22 based on genomic position (x-axis). Gray curves connect loci that overlap between different traits (PPH4 > 0.8, P-value < 5e-5), highlighting shared genetic architecture. If a locus is unique to a trait, no connection is drawn.

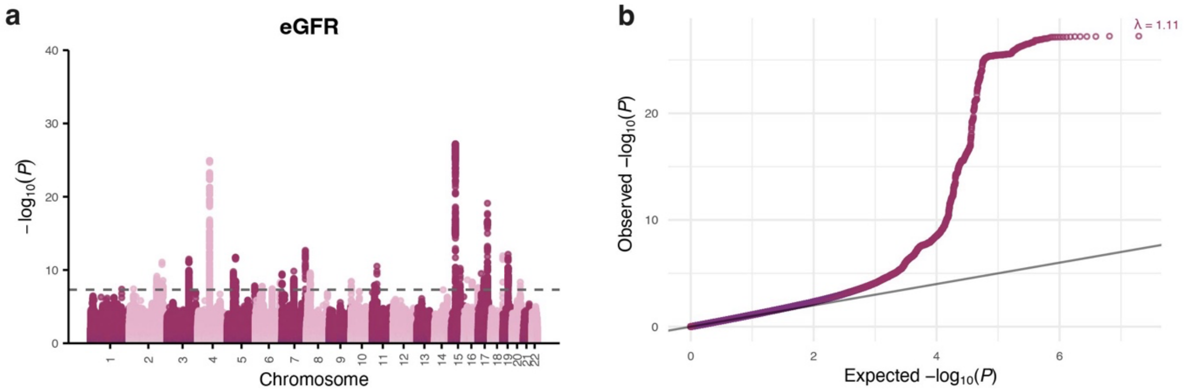

Supplementary Figure S9: GWAS results for eGFR. (a) Manhattan plot. The dashed lines indicate the genome-wide significance threshold (5e-8). (b) Quantile-quantile (QQ) plot and inflation (λ) for eGFR GWAS. eGFR was calculated using creatinine using CKDEpi.creat from nephro R package.

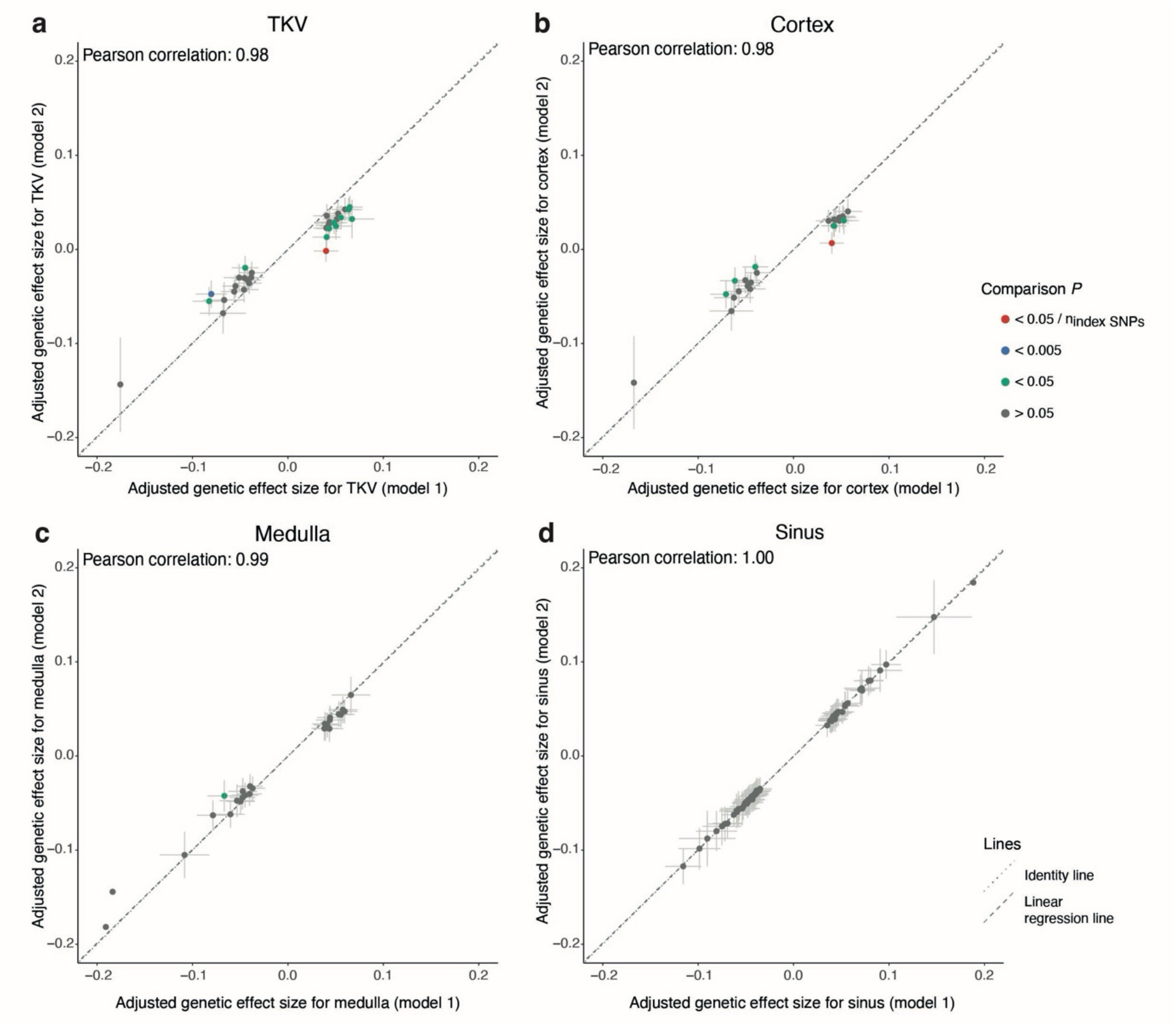

Supplementary Figure S10: Sensitivity analyses after inclusion of eGFR. Comparison of effect sizes before (model 1) and after (model 2) inclusion of eGFR as an additional covariate in GWAS of total kidney volume (TKV) (a), cortex (b), medulla (c) and sinus (d) volumes. Each point represents an index SNP, with colors indicating the level of statistical significance of the difference in effect sizes between the two GWAS analyses (Methods). Error bars correspond to 95% confidence intervals. Units correspond to the standard deviation of the respective traits.

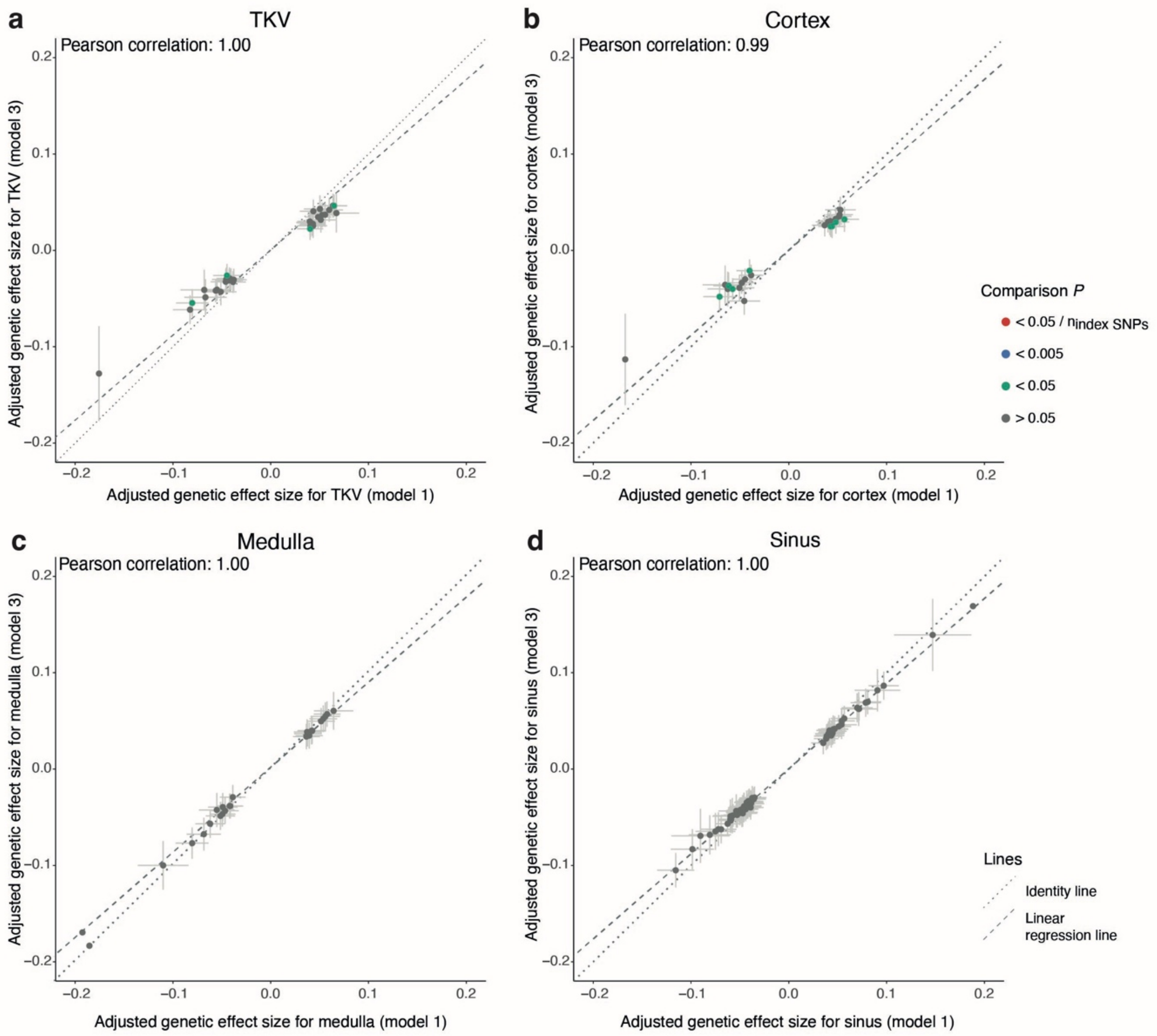

Supplementary Figure S11: Sensitivity analyses omitting the adjustment of kidney volumes for body surface area (BSA). Comparison of effect sizes before (model 3) and after (model 1) BSA-normalization in GWAS of total kidney volume (TKV) (a), cortex (b), medulla (c) and sinus (d) volumes. Each point represents an index SNP, with colors indicating the level of statistical significance of the difference in effect sizes between the two GWAS analyses (Methods). Error bars correspond to 95% confidence intervals. Units correspond to the standard deviation of the respective traits.

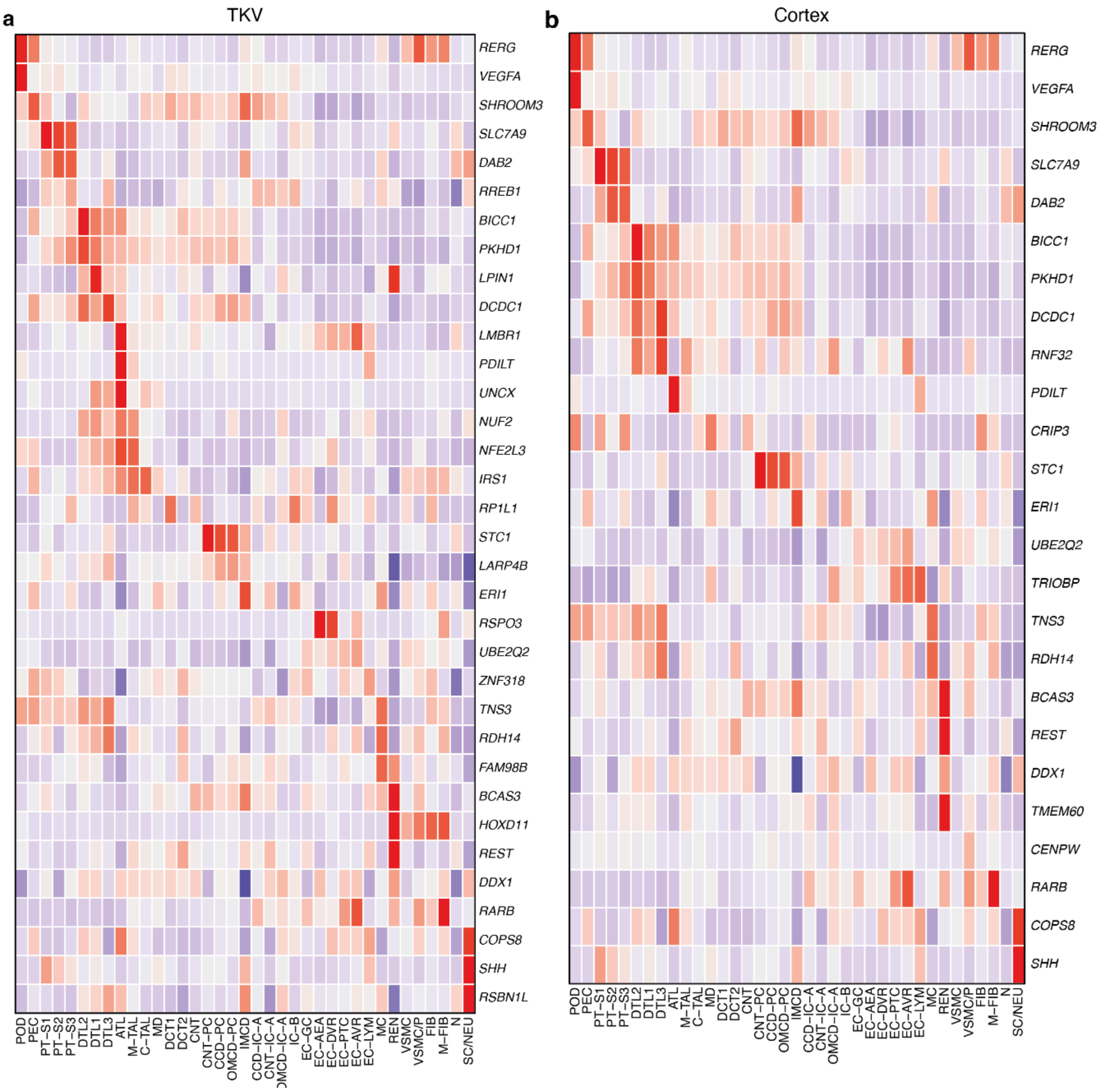

a
TKV
RERG
VEGFA
SHROOM3
SLC7A9
DAB2
RREB1
BICC1
PKHD1
LPIN1
DCDC1
LMBR1
PDILT
UNCX
NUF2
NFE2L3
IRS1
RP1L1
STC1
LARP4B
ERI1
RSPO3
UBE2Q2
ZNF318
TNS3
RDH14
FAM98B
BCAS3
HOXD11
REST
DDX1
RARB
COPS8
SHH
RSBN1L
POD
PEC
PT−S1
PT−S2
PT−S3
DTL2
DTL1
DTL3
ATL
M−TAL
C−TAL
MD
DCT1
DCT2
CNT
CNT−PC
CCD−PC
OMCD−PC
IMCD
CCD−IC−A
CNT−IC−A
IC−B
EC−GC
EC−AEA
EC−DVR
EC−PTC
EC−AVR
EC−LYM
MC
REN
VSMC
VSMC/P
FIB
M−FIB
N
SC/NEU
b
Cortex
RERG
VEGFA
SHROOM3
SLC7A9
DAB2
BICC1
PKHD1
DCDC1
RNF32
PDILT
CRIP3
STC1
ERI1
UBE2Q2
TRIOBP
TNS3
RDH14
BCAS3
REST
DDX1
TMEM60
CENPW
RARB
COPS8
SHH
POD
PEC
PT−S1
PT−S2
PT−S3
DTL2
DTL1
DTL3
ATL
M−TAL
C−TAL
MD
DCT1
DCT2
CNT
CNT−PC
CCD−PC
OMCD−PC
IMCD
CCD−IC−A
CNT−IC−A
OMCD−IC−A
IC−B
EC−GC
EC−AEA
EC−DVR
EC−PTC
EC−AVR
EC−LYM
MC
REN
VSMC
VSMC/P
FIB
M−FIB
N
SC/NEU

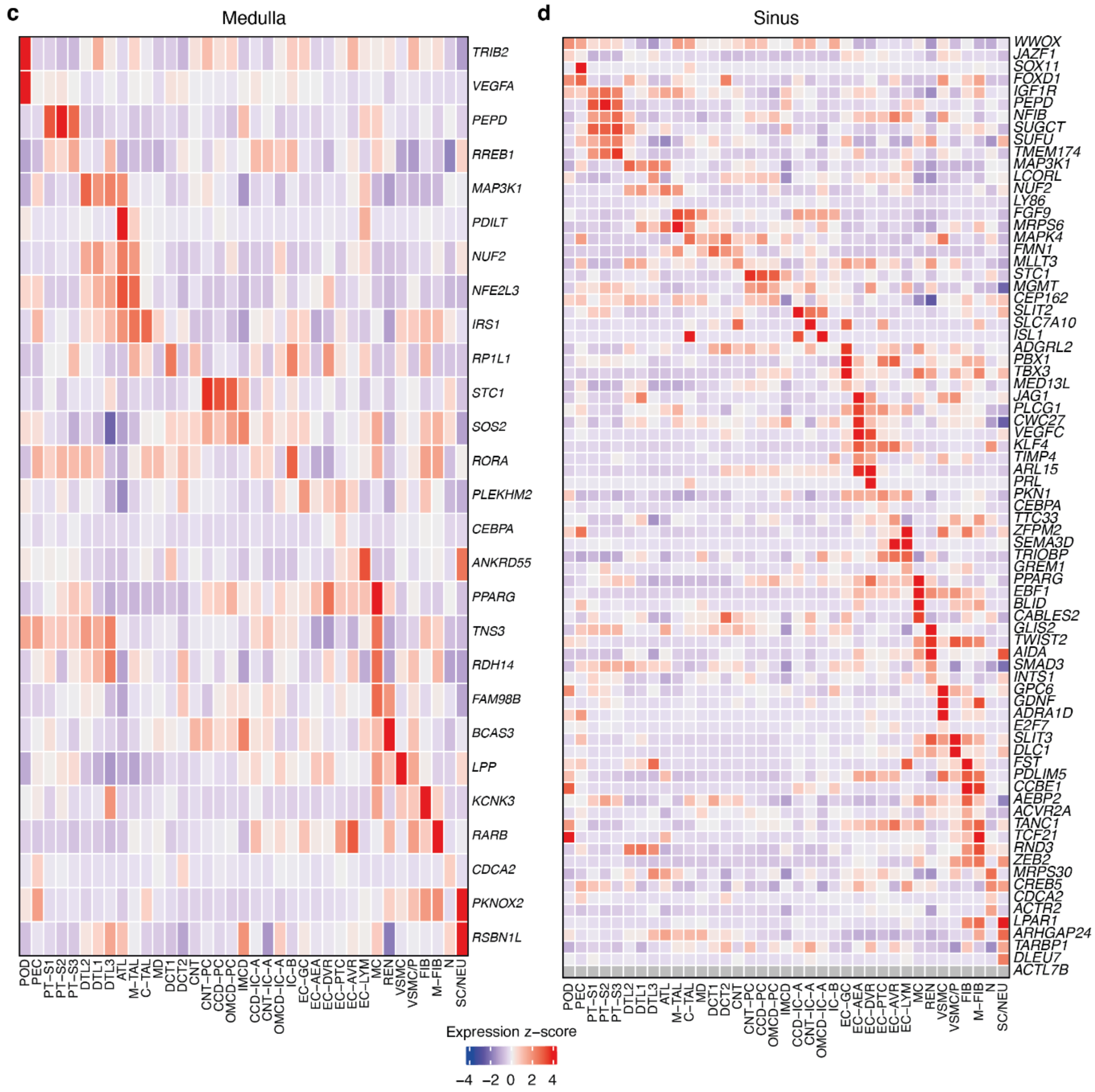

Supplementary Figure S12: Expression patterns of prioritized genes in loci associated with each kidney volume. Heatmaps showing KPMP snRNA-seq expression levels for TKV (a), cortex (b), medulla (c), and sinus (d) volumes.

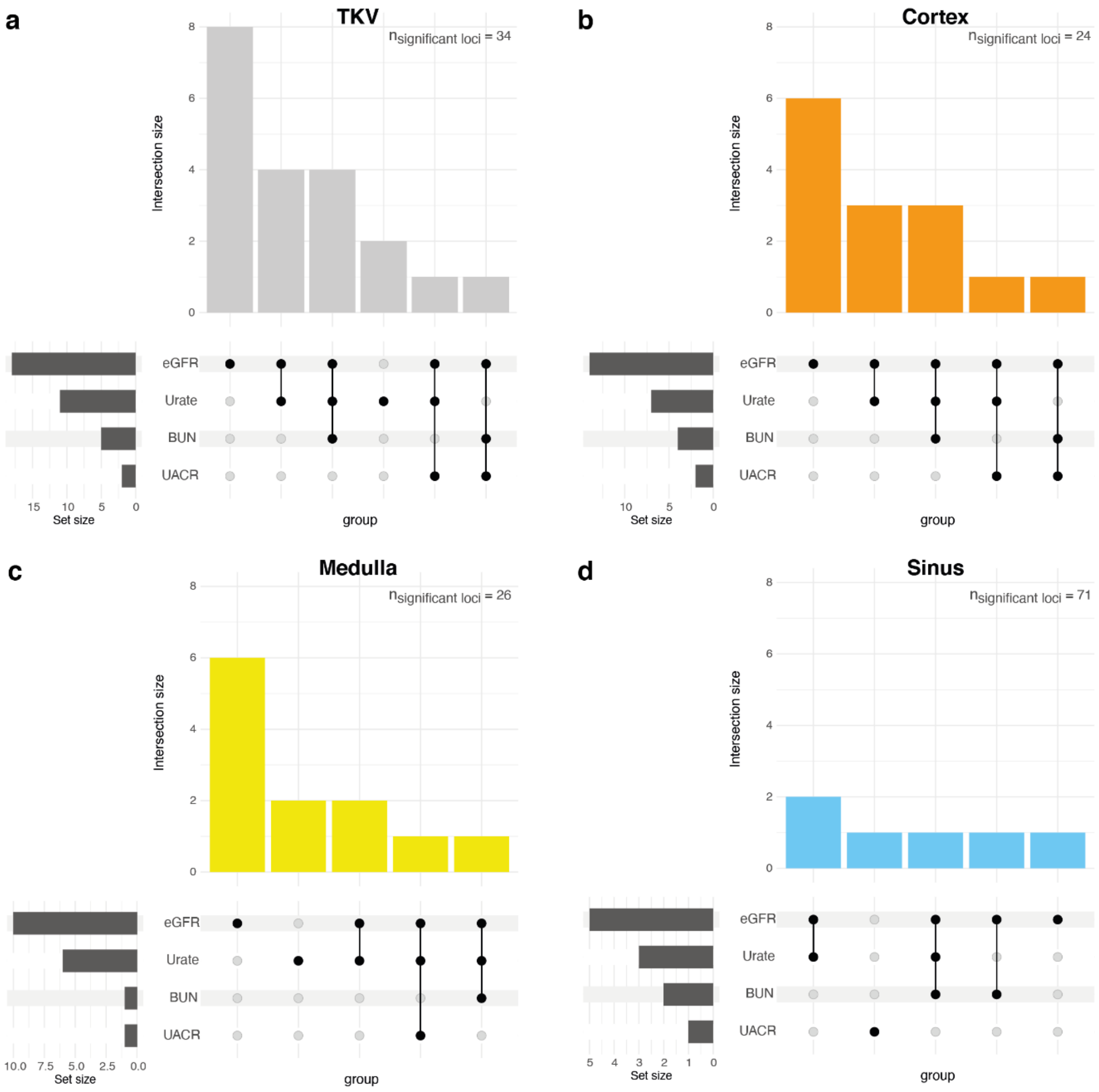

Supplementary Figure S13: UpSet plots showing overlap of positive colocalization (PPH4 > 0.8) of significant loci for TKV (a), cortex (b), medulla (c), and sinus (d) volumes with the kidney function markers eGFR, urate, BUN, and UACR. Set size bars (left) indicate the number of significant loci for each kidney function trait, and intersection size bars (top) show the number of colocalized loci for each trait combination.
