# Supplementary material for: Genetic screens of imaging-derived kidney volumes identify genes linked to kidney function
Source: Kidney Int. Author manuscript; Available in PMC 2026 Jul 5. (PMC13333066; doi:10.1016/j.kint.2025.08.038)

TKV

region chr1__163201840-164201840

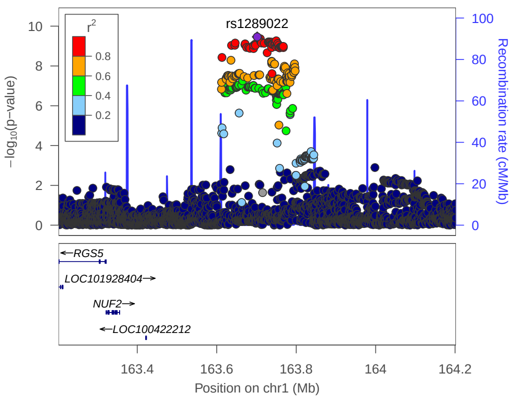

region chr2__11465317-12465317

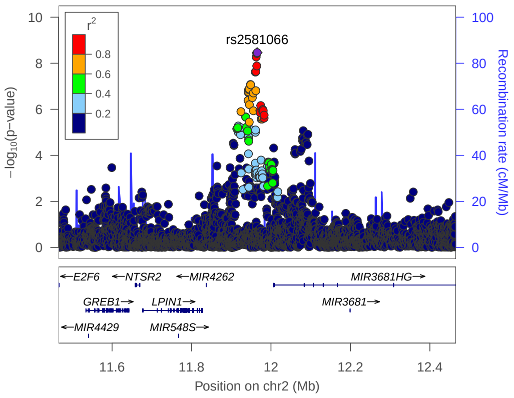

region chr2__15142347-16142347

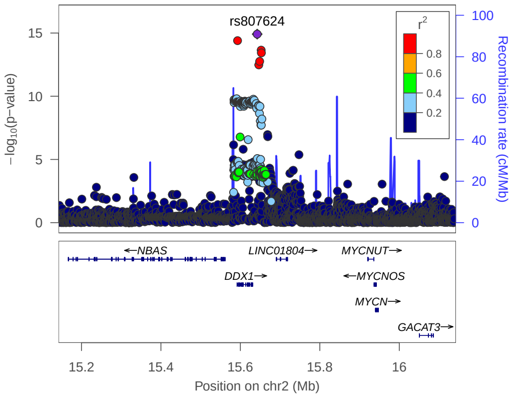

region chr2__17999475-18999475

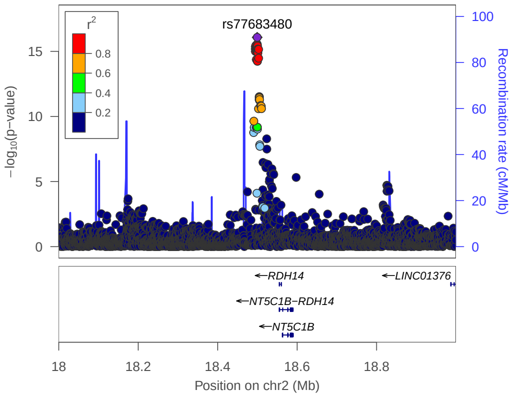

region chr2__175609376-176609376

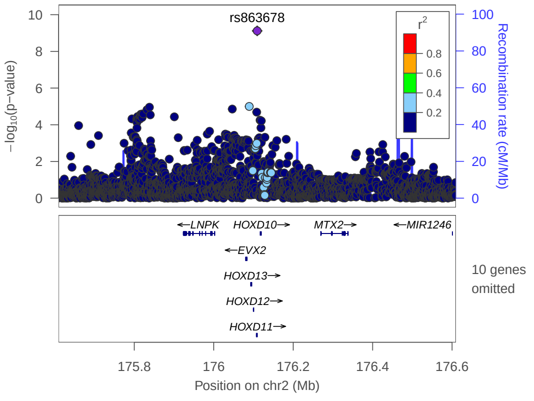

region chr2__225727434-226727434

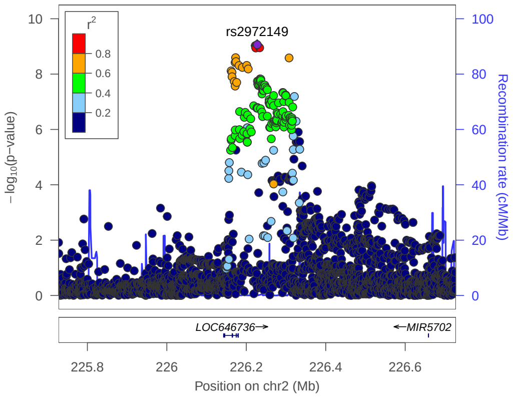

region chr2__236426112-237426112

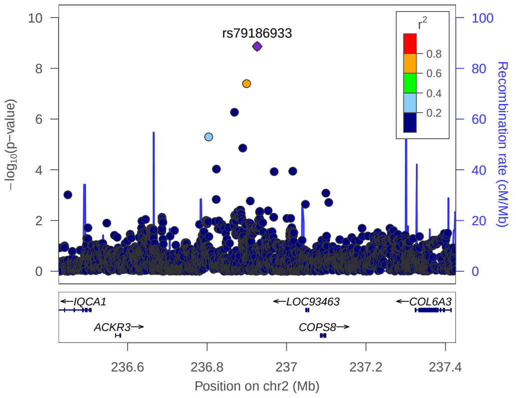

region chr3__24883438-25883438

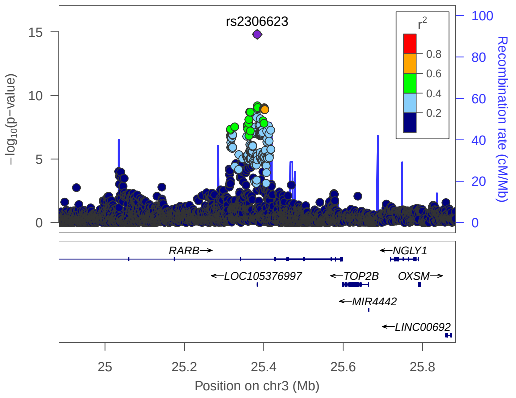

region chr4__56386384-57386384

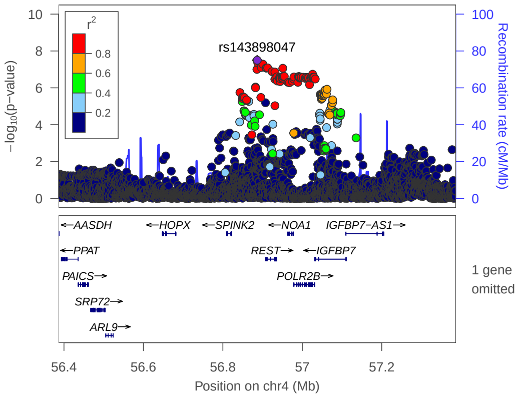

region chr4__75989165-76989165

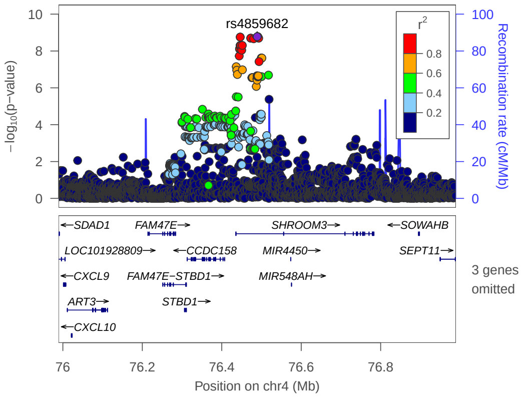

region chr5__38926205-39926205

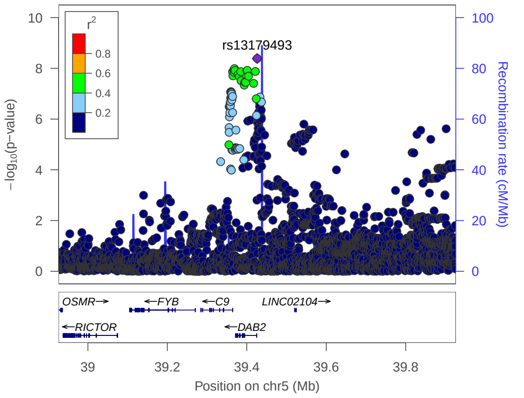

region chr6__6632825-7632825

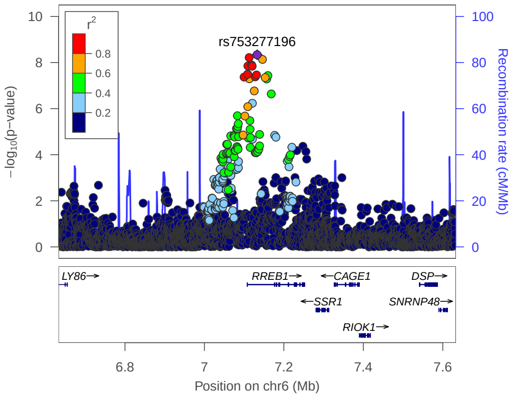

region chr6_42792922-44336366 has >1 independent SNPS

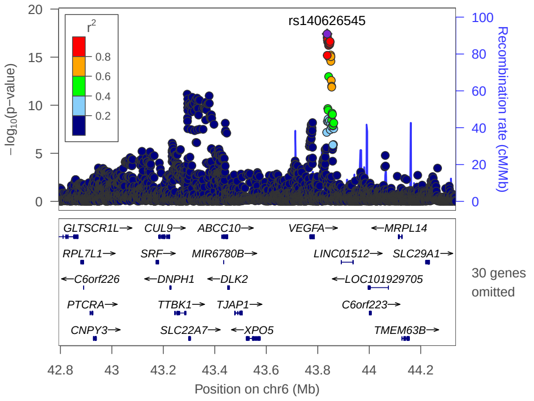

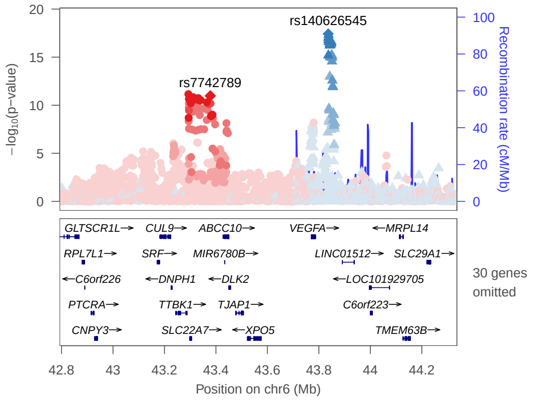

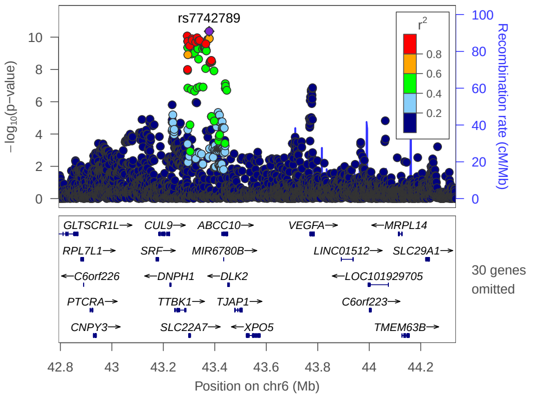

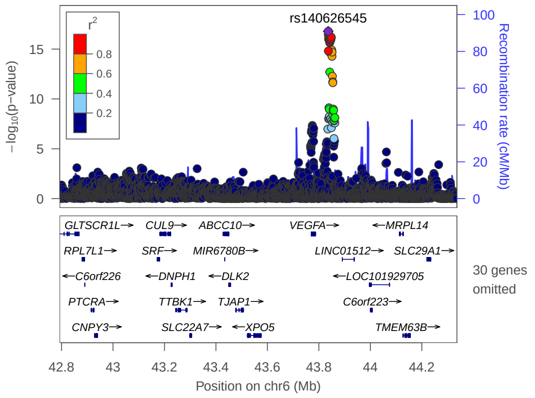

region chr6_49391760-52045803

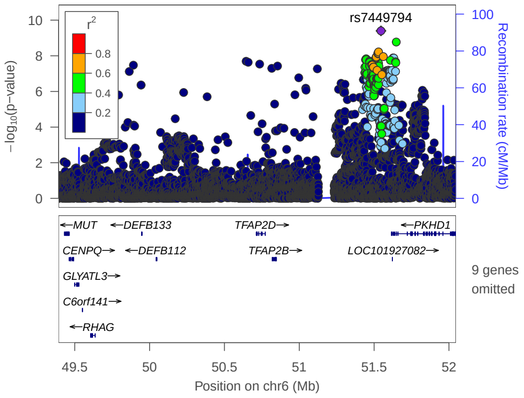

region chr6__126358586-127358586

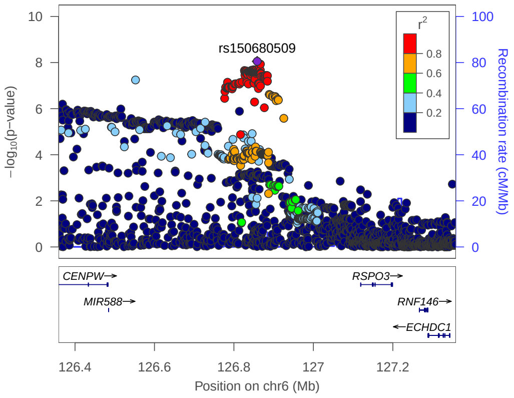

region chr7__746556-1746556

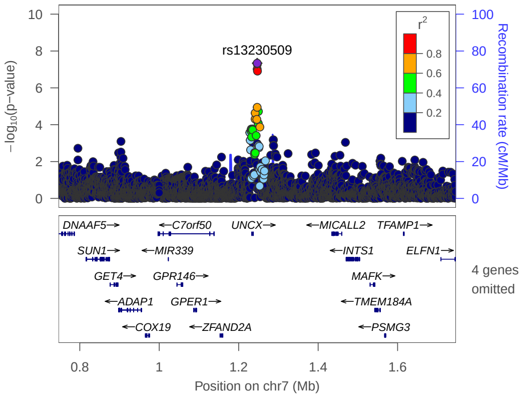

region chr7__25253637-26253637

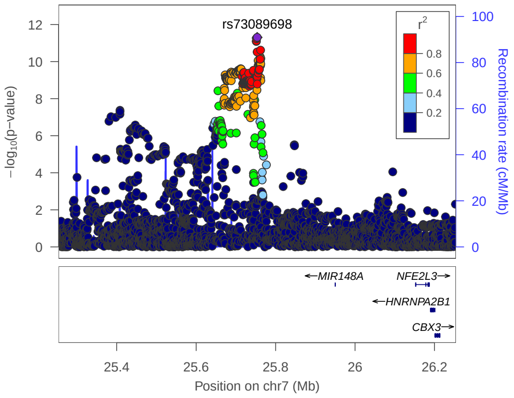

region chr7__46213893-47213893

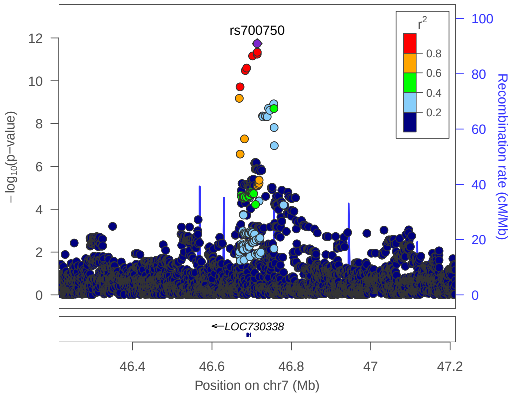

region chr7__77261063-78261063

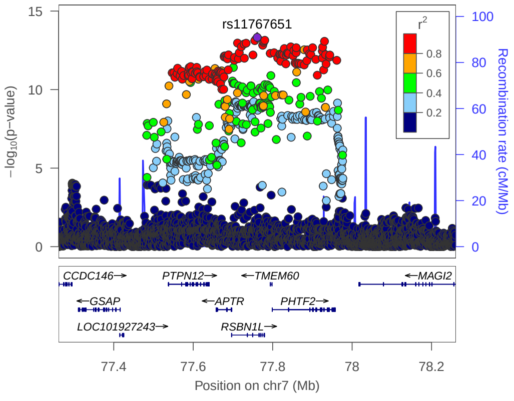

region chr7__155312377-156841391 has >1 independent SNPS

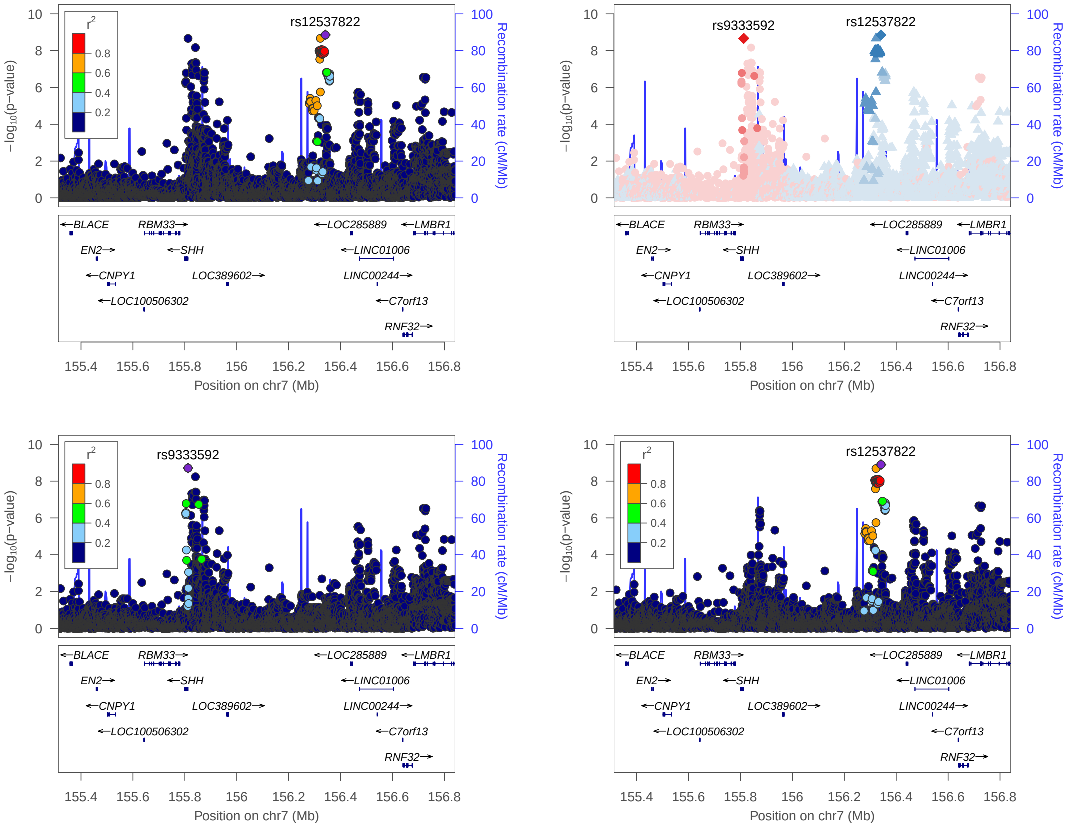

region chr8_8817108-9817108

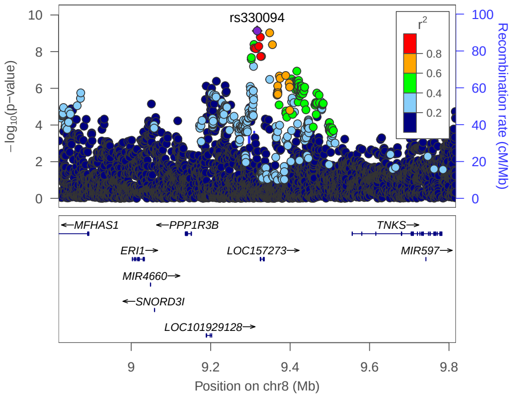

region chr8_10216192-11216192

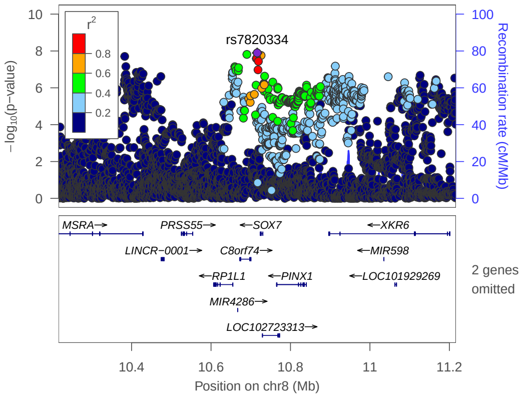

region chr8_23427074-24427074

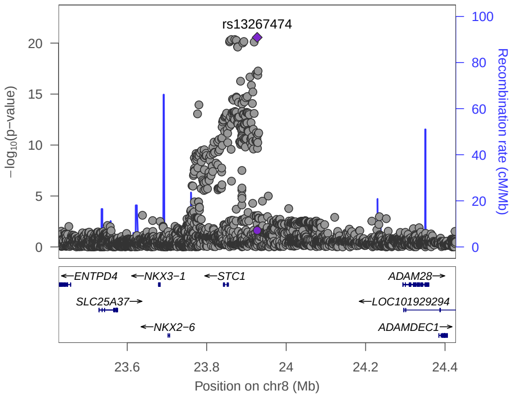

region chr10_379507-1379507

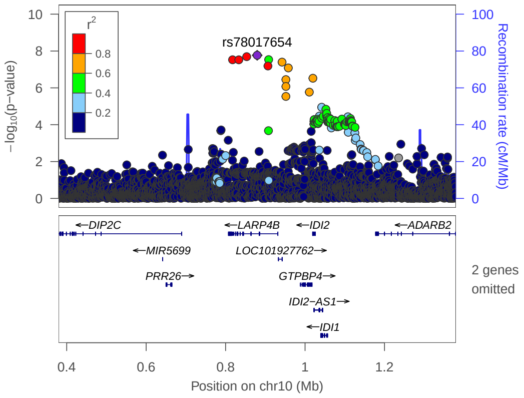

region chr10_58025115-59025115

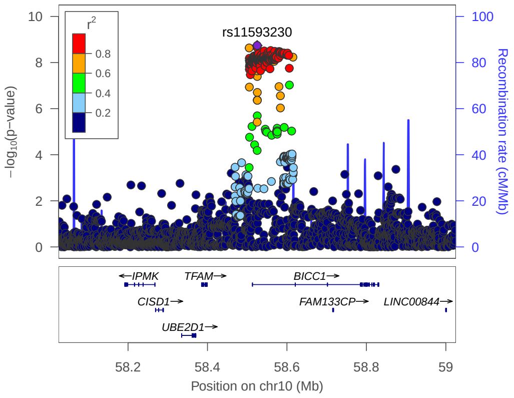

region chr11_1600782-2600782

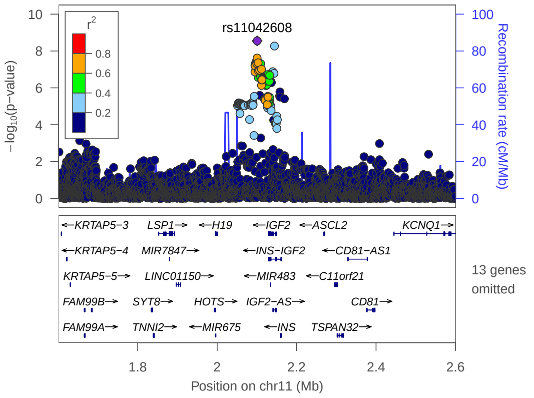

region chr11_30238234-31238234 has >1 independent SNPS

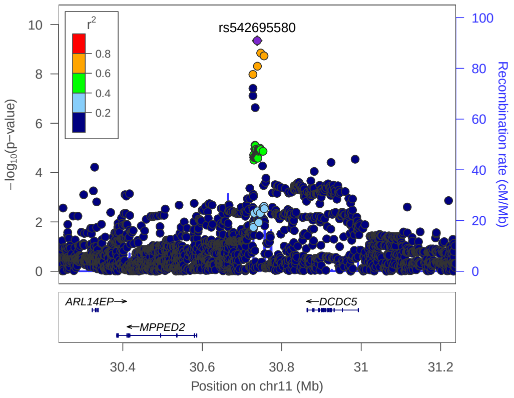

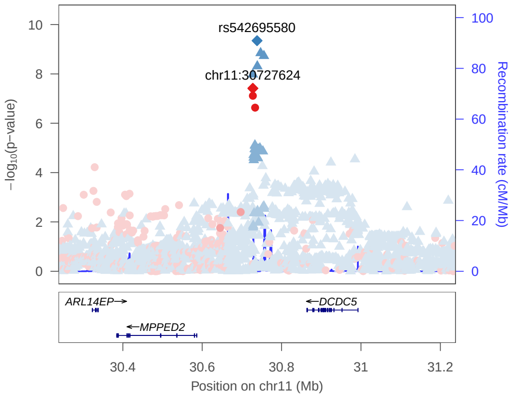

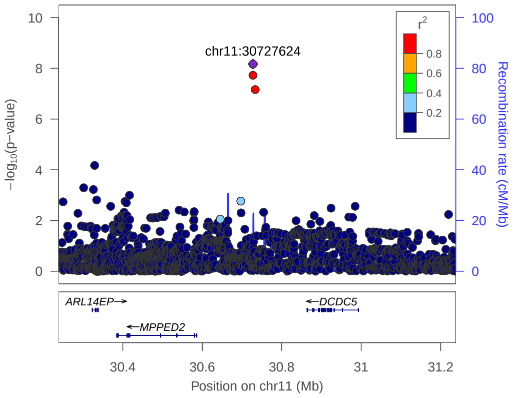

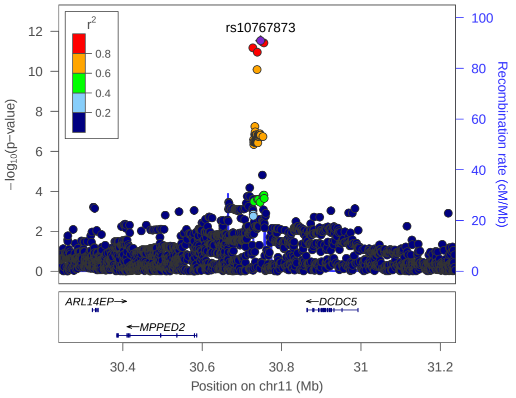

region chr12__14668987-15668987

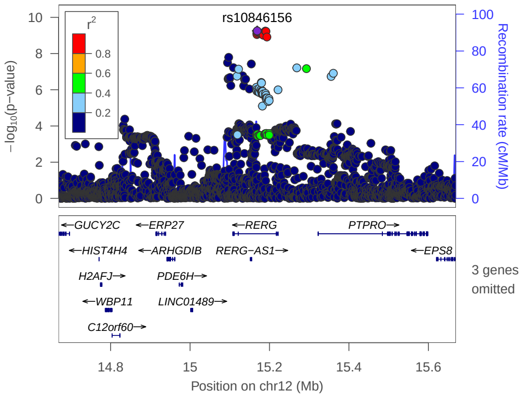

region chr15__38486960-39486960

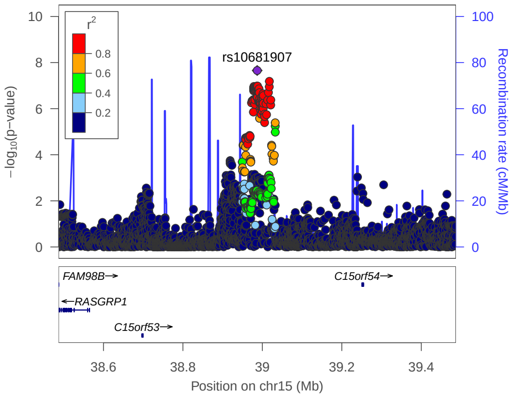

region chr15__75343853-76343853

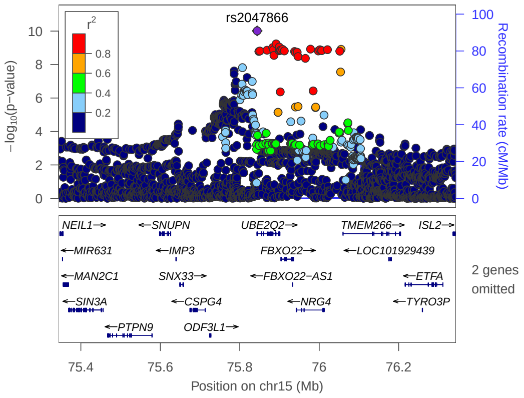

region chr16__19881010-20881010

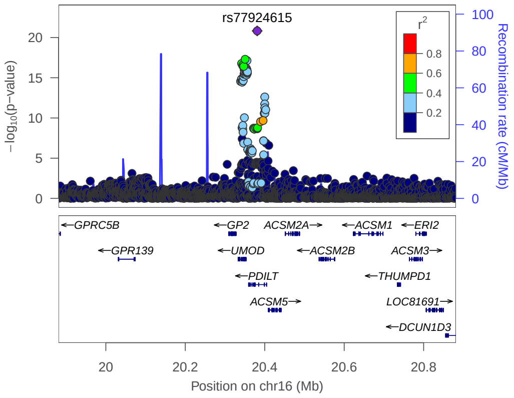

region chr16__51213001-52213001

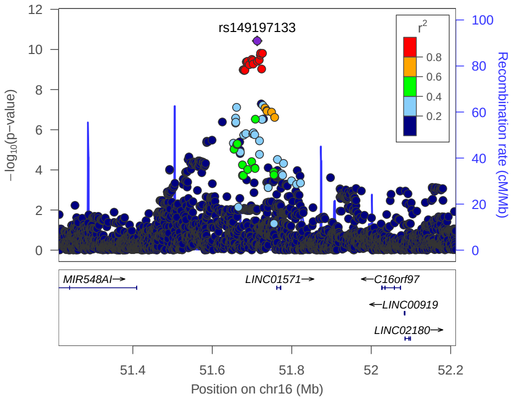

region chr17__60879228-61879228 has >1 independent SNPS

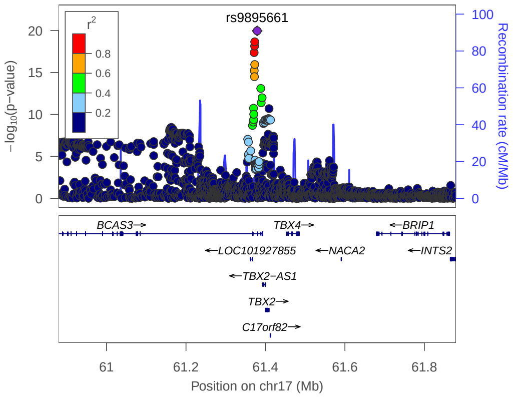

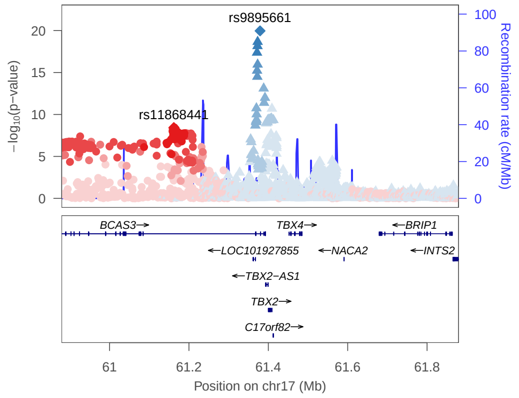

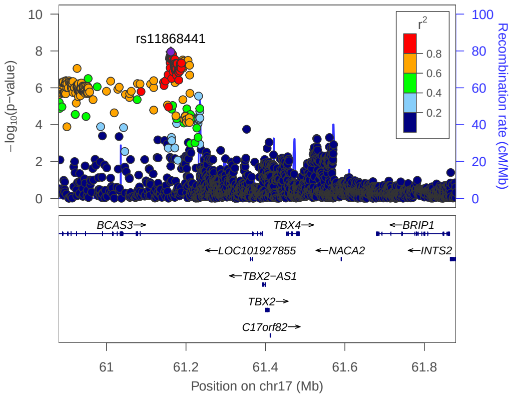

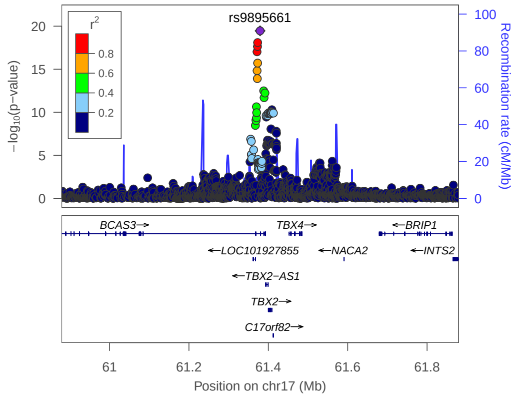

region chr19_32403184-33403184

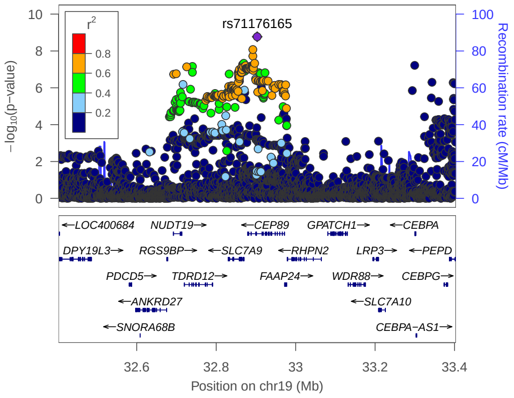

Supplement: 2 [file NIHMS2184648-supplement-2.pdf]
