# Supplementary material for: Genetic screens of imaging-derived kidney volumes identify genes linked to kidney function
Source: Kidney Int. Author manuscript; Available in PMC 2026 Jul 5. (PMC13333066; doi:10.1016/j.kint.2025.08.038)
Supplement: 3 [file NIHMS2184648-supplement-3.pdf]

Cortex

region chr2__15142347-16142347

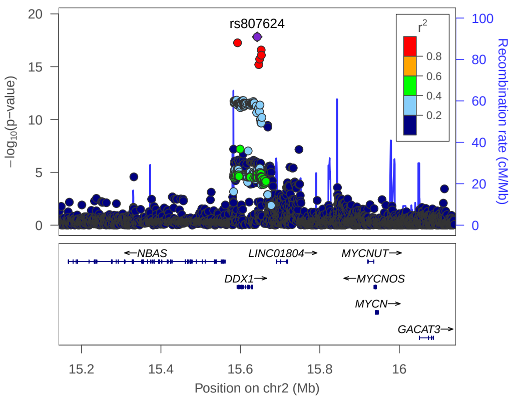

region chr2__17999475-18999475

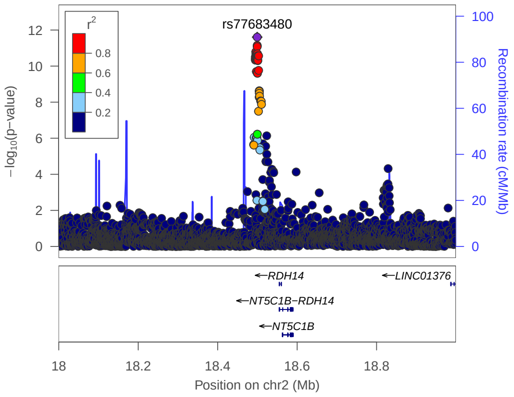

region chr2__236426112-237426112

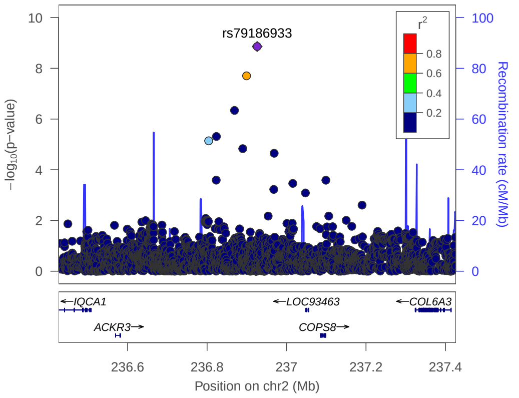

region chr3__24883438-25883438

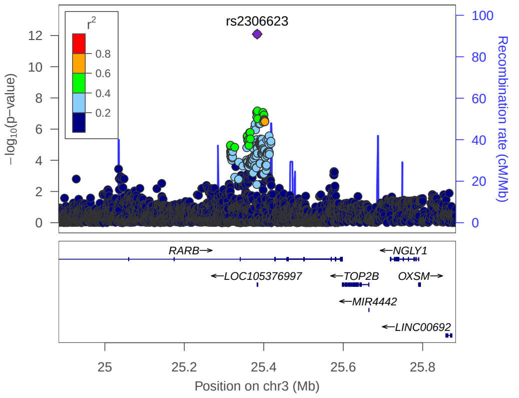

region chr4__56386384-57386384

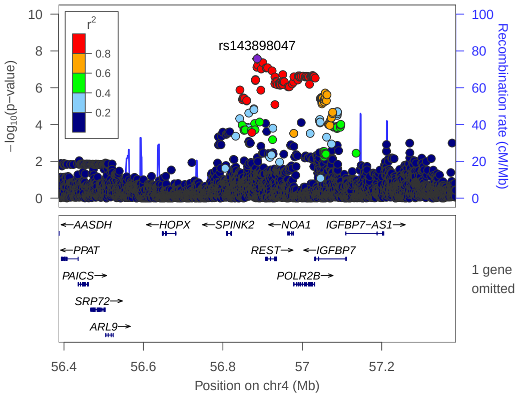

region chr4__75946427-76946427

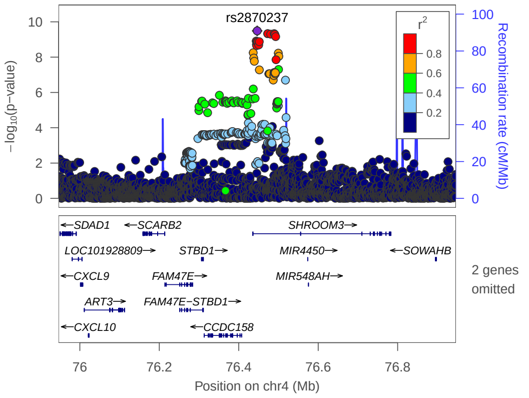

region chr5__38926205-40515246

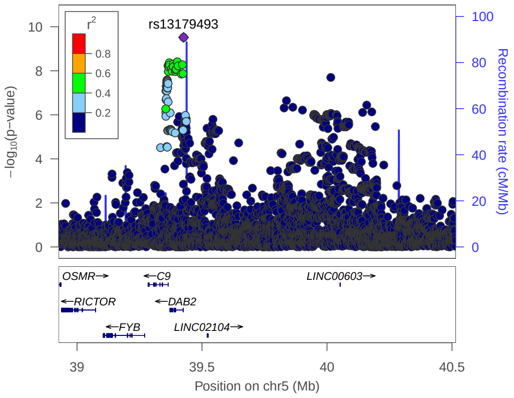

region chr6__42792922-44336366 has >1 independent SNPS

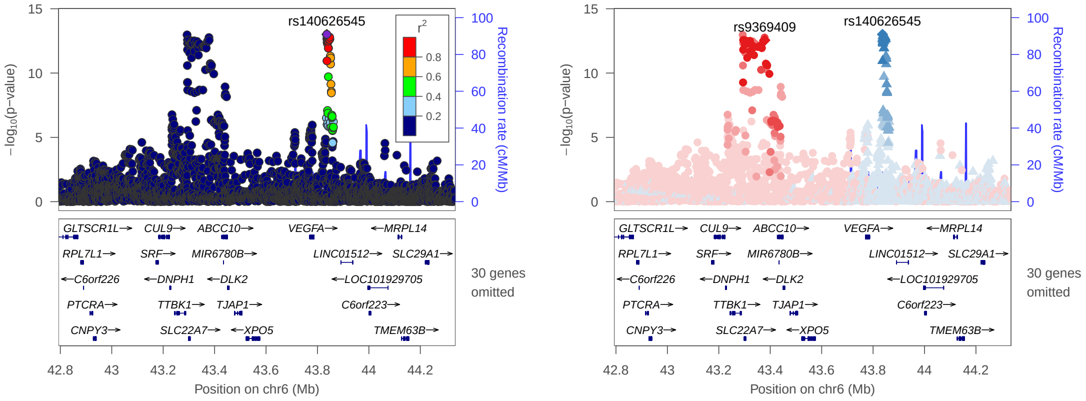

region chr6__50620816-52147909

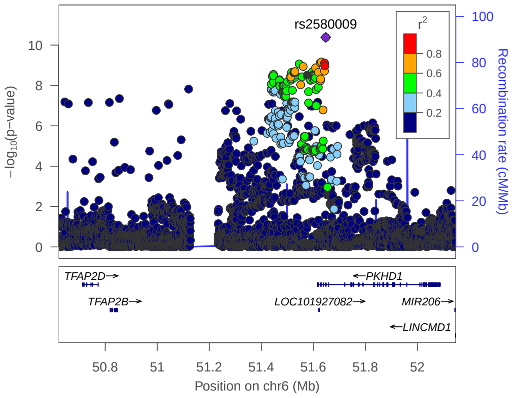

region chr6__126052277-127052277

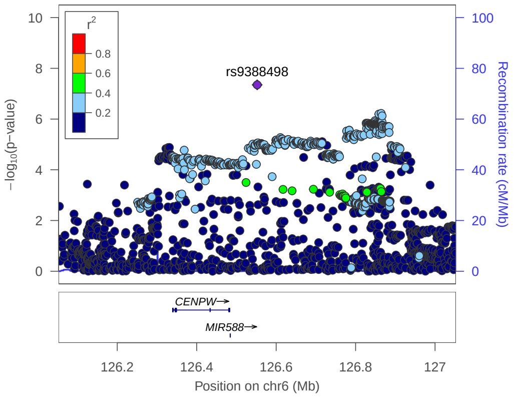

region chr7__46213893-47213893

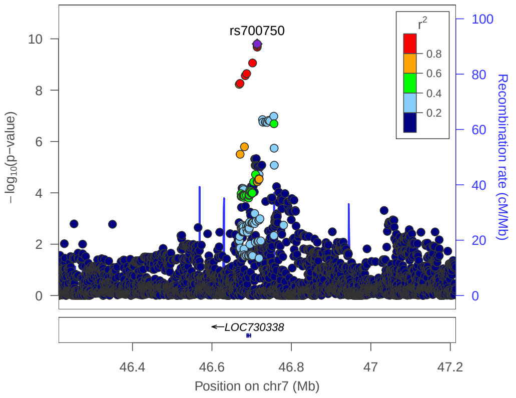

region chr7__77261063-78261063

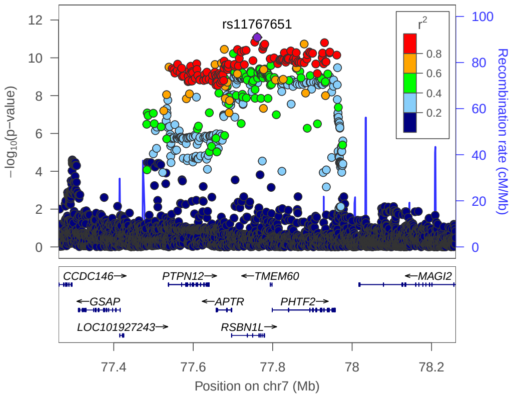

region chr7__155312377-156841391 has >1 independent SNPS

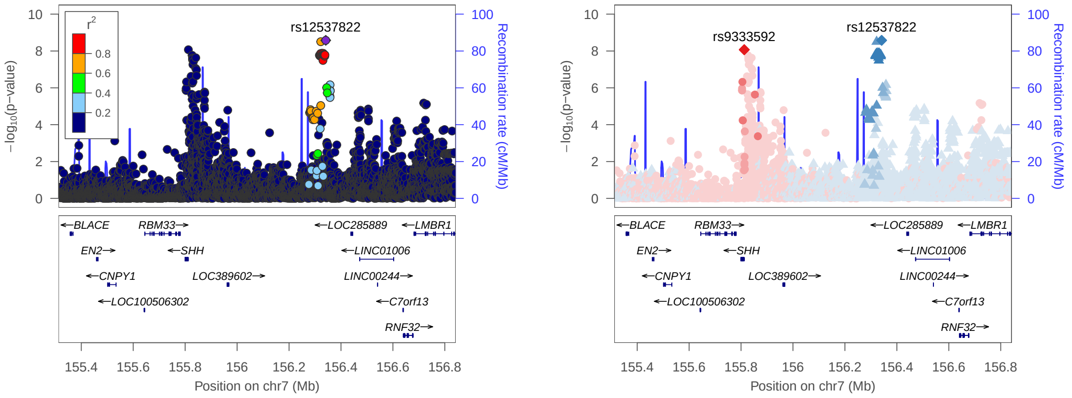

region chr8__8815699-9815699

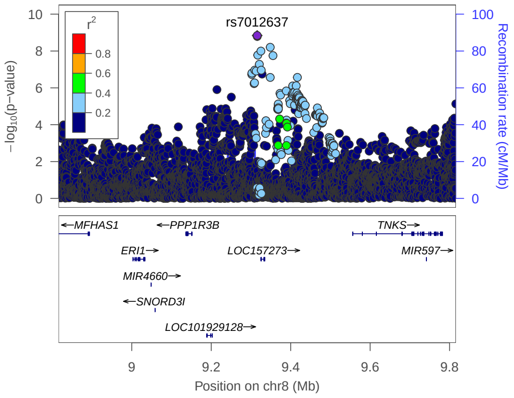

region chr8__23419493-24419493

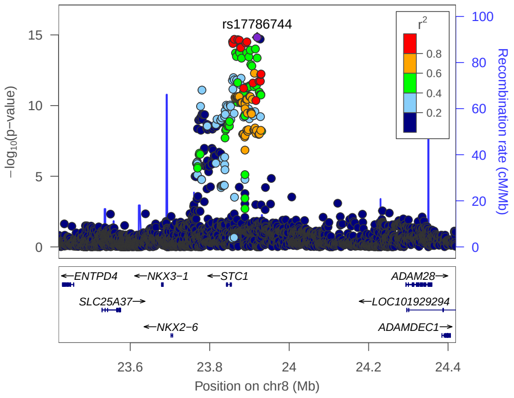

region chr10__58059106-59059106

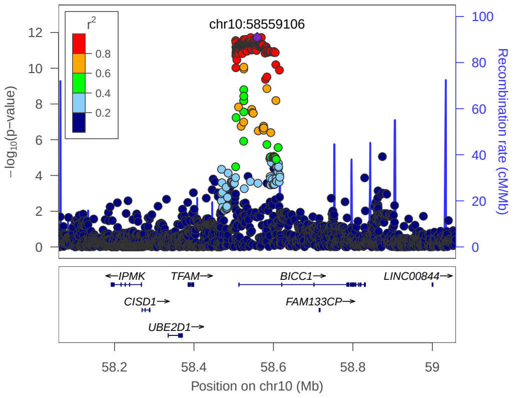

region chr11__30238234-31238234

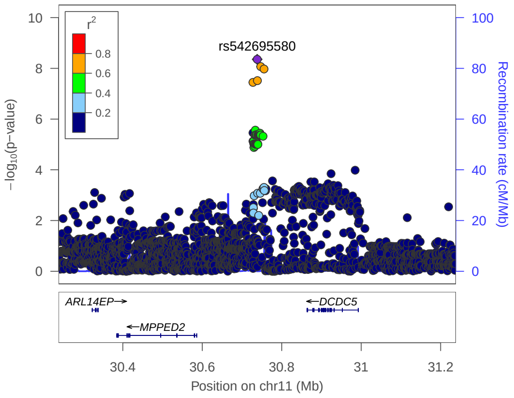

region chr12__14668987-15668987

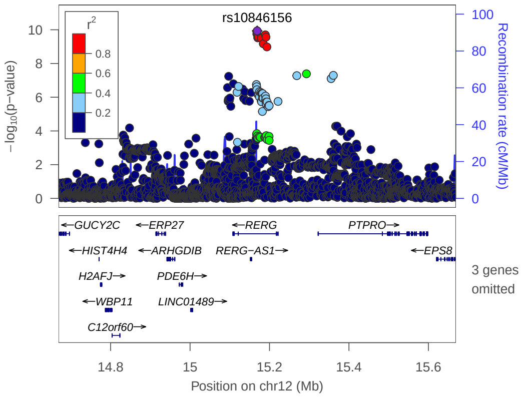

region chr15_75343853-76343853

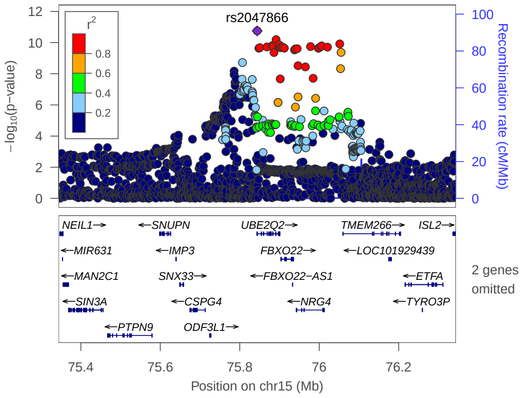

region chr16_19881010-20881010

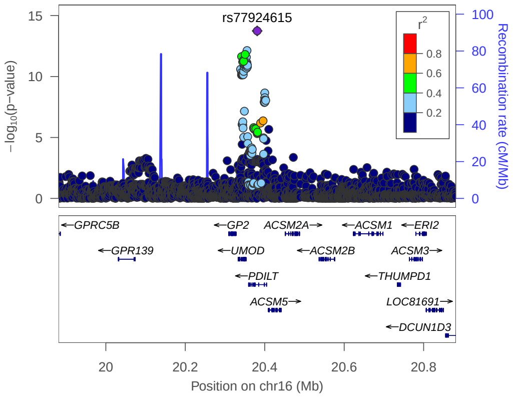

region chr16_51221460-52221460

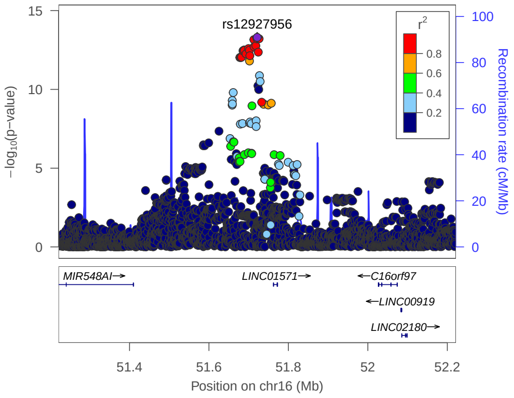

region chr17__60373430-61879228 has >1 independent SNPS

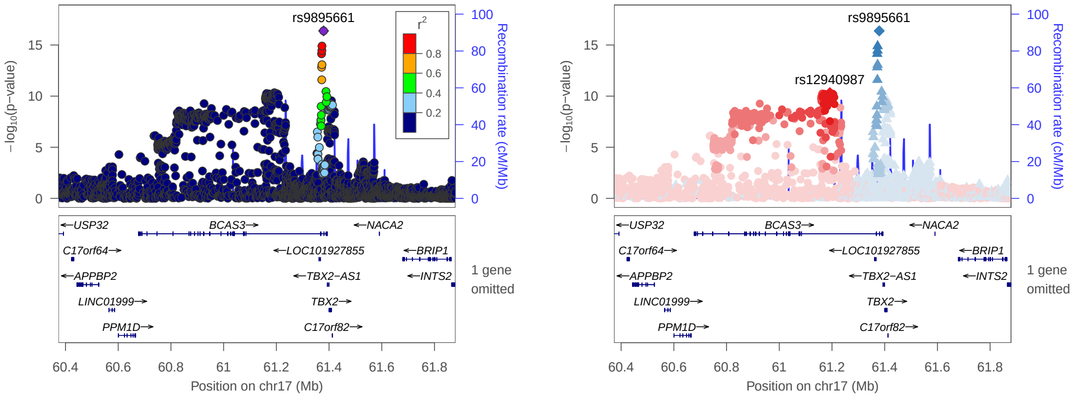

region chr19__32403184-33403184

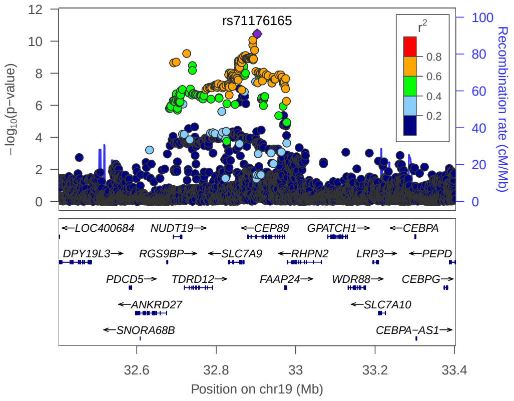

region chr22__37288634-38288634

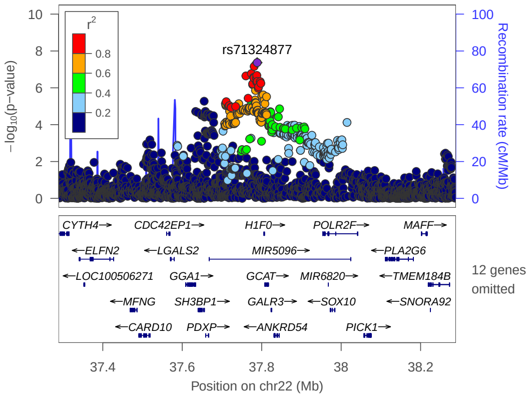
rs71324877
r2
0.8
0.6
0.4
0.2
−log10(p−value)
Recombination rate (cM/Mb)
CYTH4→
CDC42EP1→
H1F0→
POLR2F→
MAFF→
←ELFN2
←LGALS2
MIR5096→
←PLA2G6
←LOC100506271
GGA1→
GCAT→
MIR6820→
←TMEM184B
←MFNG
SH3BP1→
GALR3→
←SOX10
←SNORA92
←CARD10
PDXP→
←ANKRD54
PICK1→
12 genes omitted
Position on chr22 (Mb)
