# Supplementary material for: Genetic screens of imaging-derived kidney volumes identify genes linked to kidney function
Source: Kidney Int. Author manuscript; Available in PMC 2026 Jul 5. (PMC13333066; doi:10.1016/j.kint.2025.08.038)
Supplement: 4 [file NIHMS2184648-supplement-4.pdf]

Medulla

region chr1__15155446-16155446

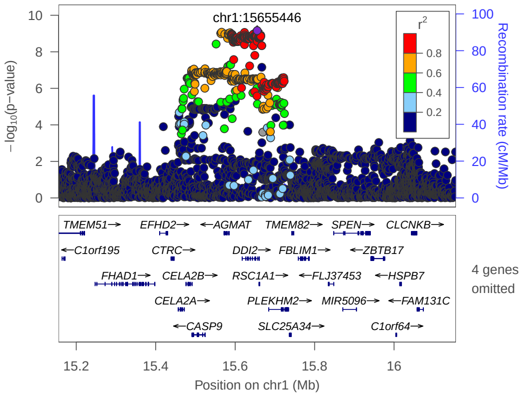

region chr1__163201840-164201840

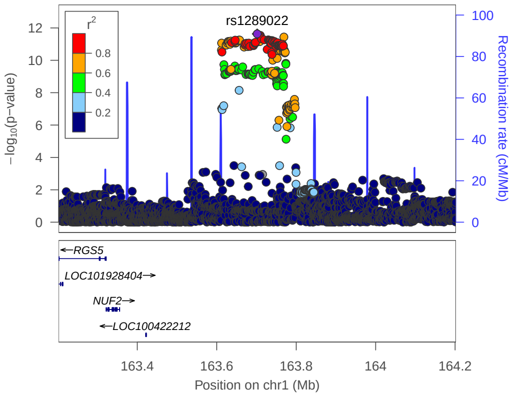

region chr2__12293865-13293865

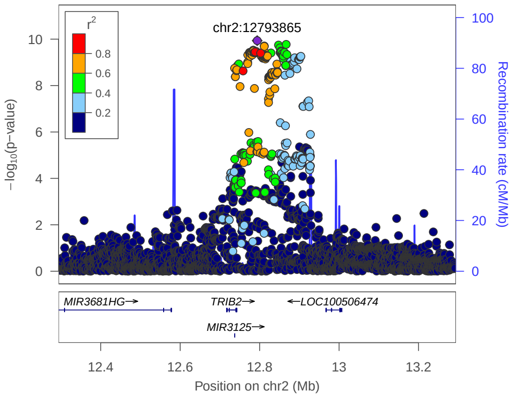

region chr2__17994999-18994999

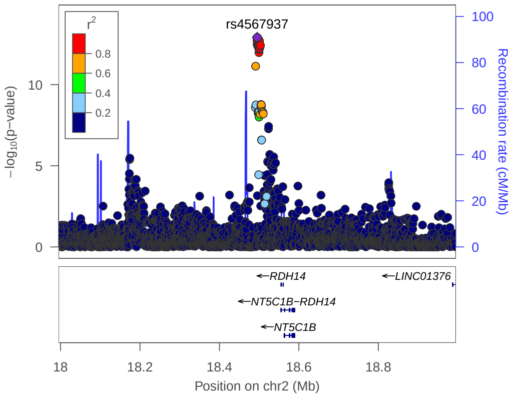

region chr2__26217426-27217426

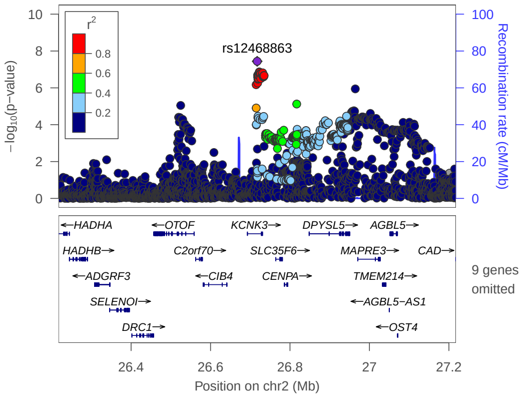

region chr2__225733671-226733671

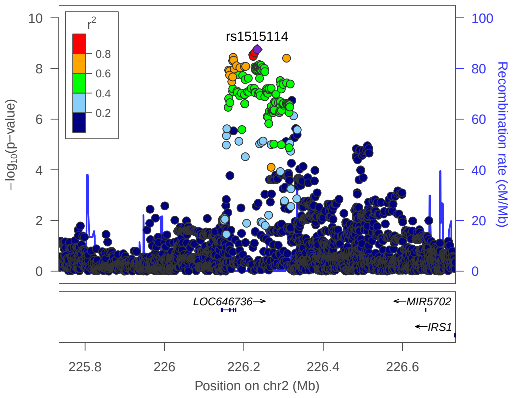

region chr3__11819385-12819385

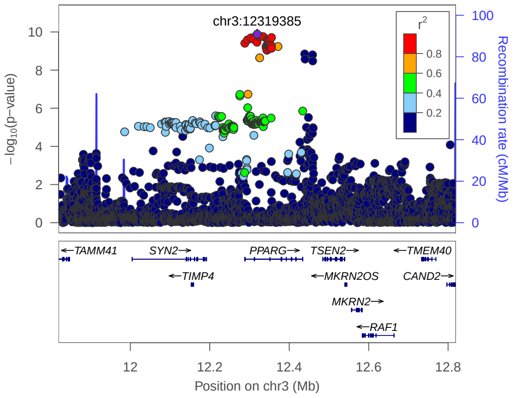

region chr3__24883438-25883438

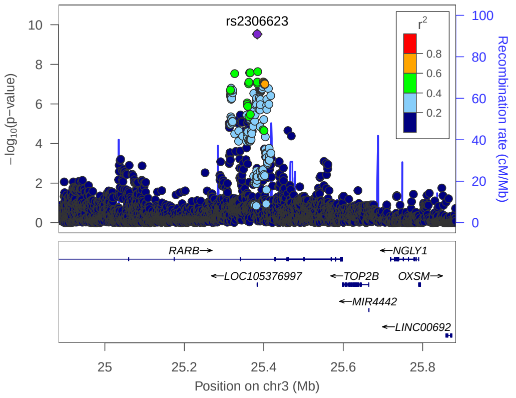

region chr3__187468766-188468766

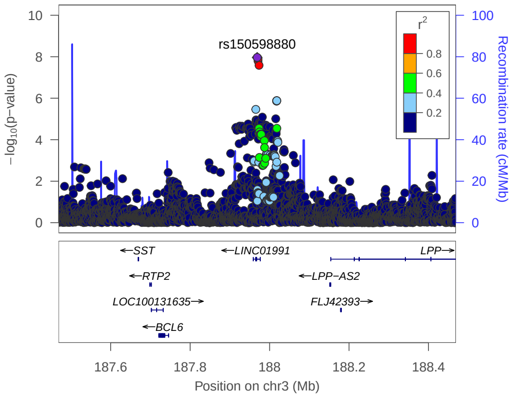

region chr5__55998805-56998805 has >1 independent SNPS

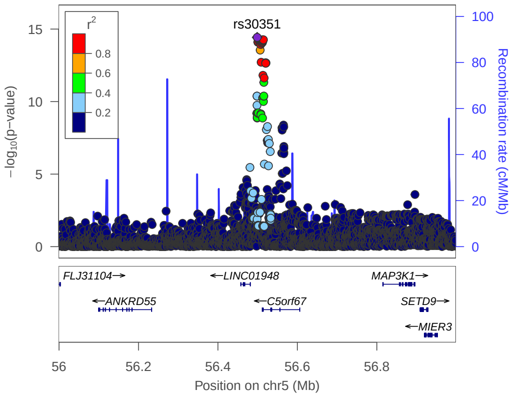

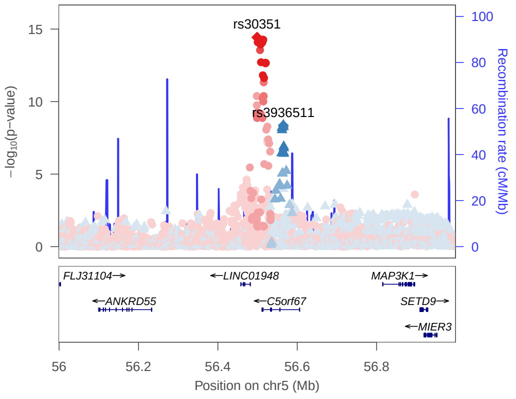

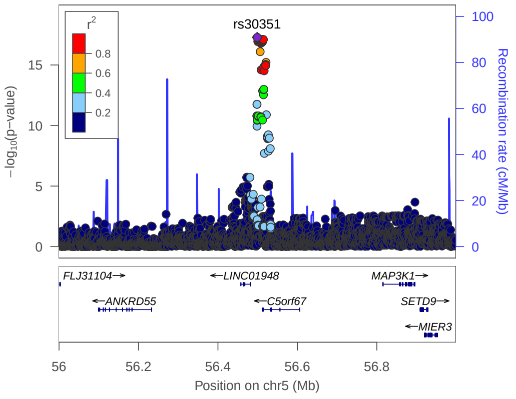

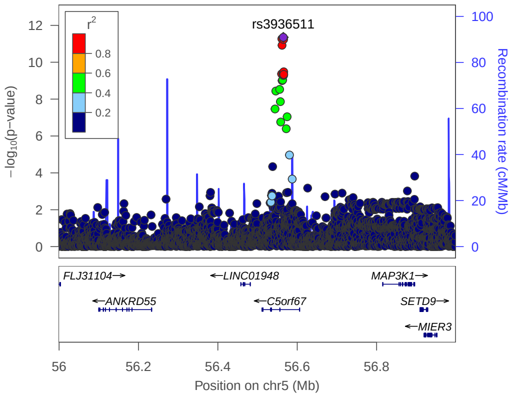

region chr6__6612586-7612586

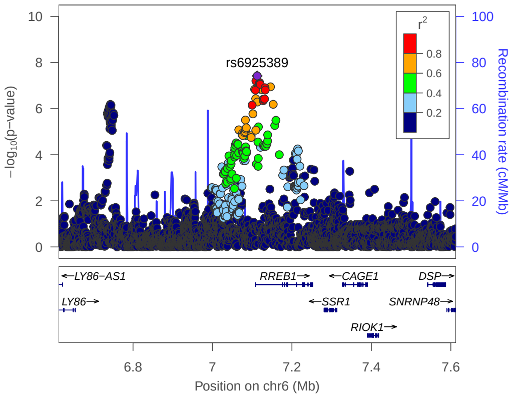

region chr6__43337625-44337625

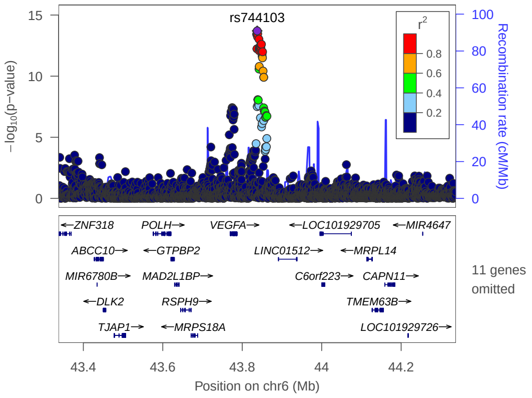

region chr7__25262140-26262140

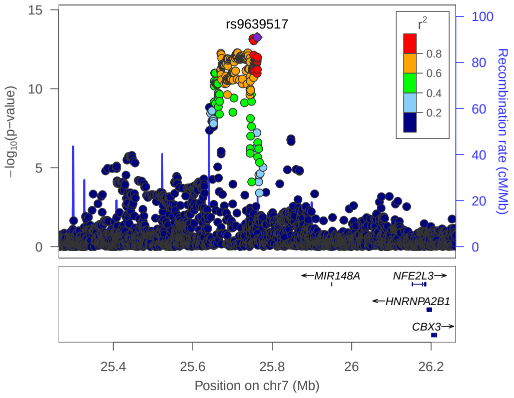

region chr7__46202245-47202245

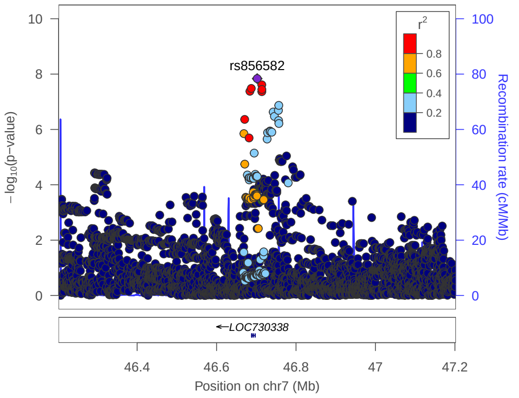

region chr7__77233172-78233172

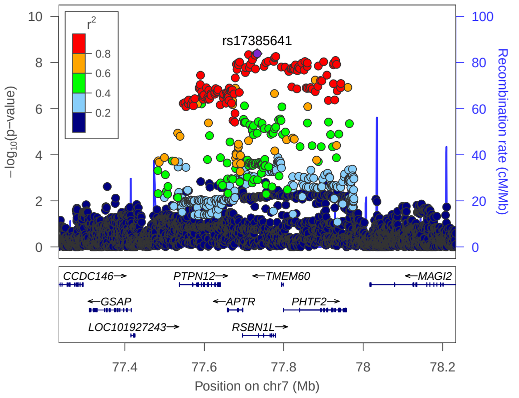

region chr8__10190248-11190248

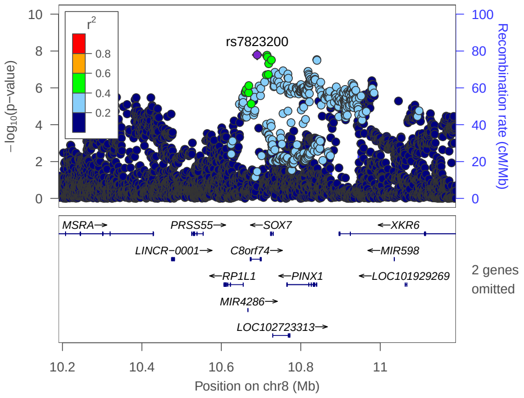

region chr8__23427074-24427074

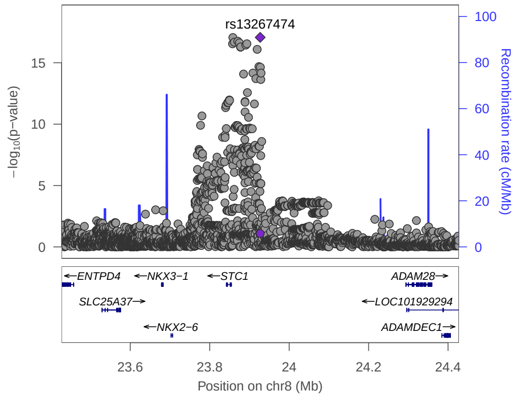

region chr8__25107154-26107154

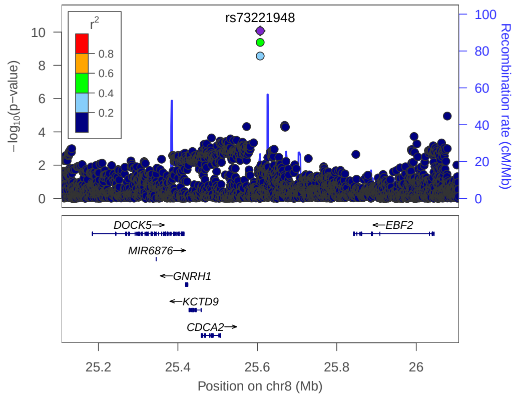

region chr11__1603205-2603205

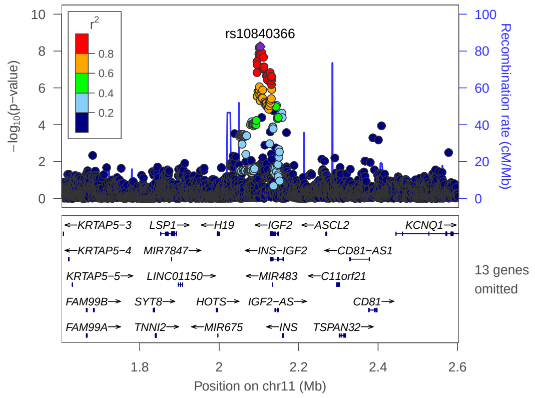

region chr11__124741650-125741650

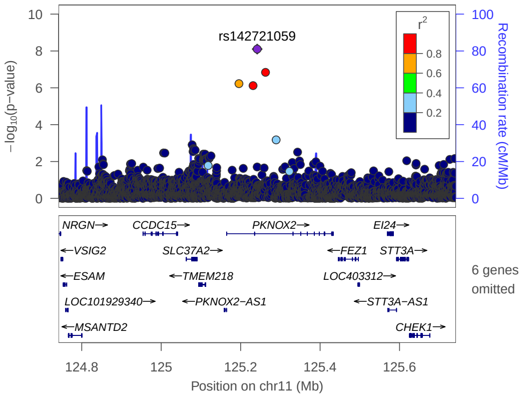

region chr14_49688639-50688639

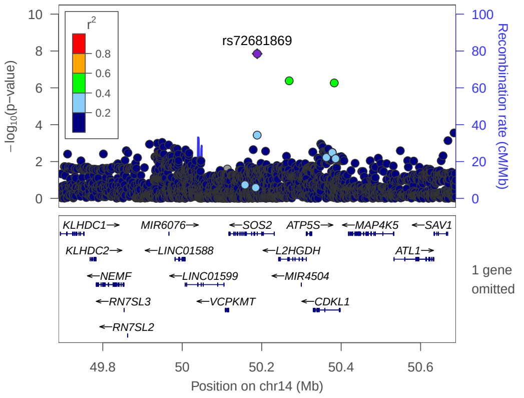

region chr15_38517614-39517614

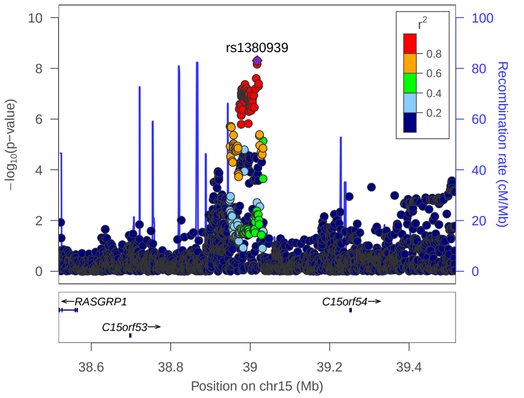

region chr15_60397812-61397812

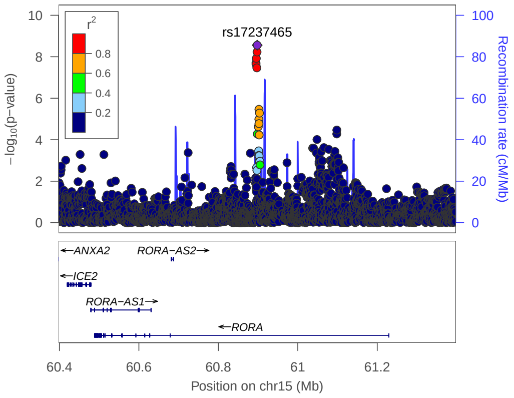

region chr16__19881010-20881010

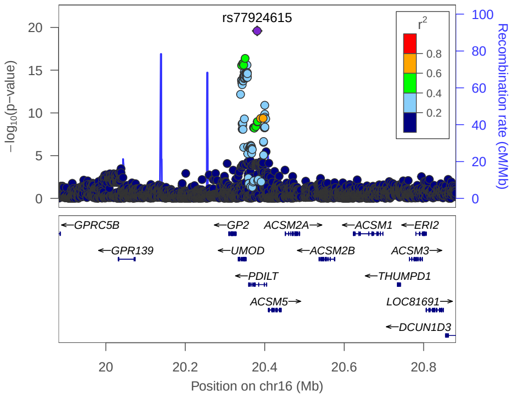

region chr17__60879228-61879228

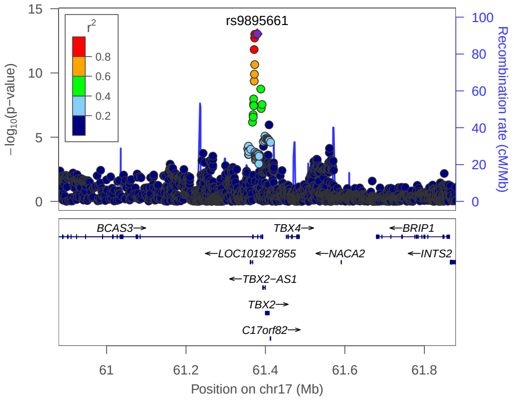

region chr19__32799650-33799650 has >1 independent SNPS

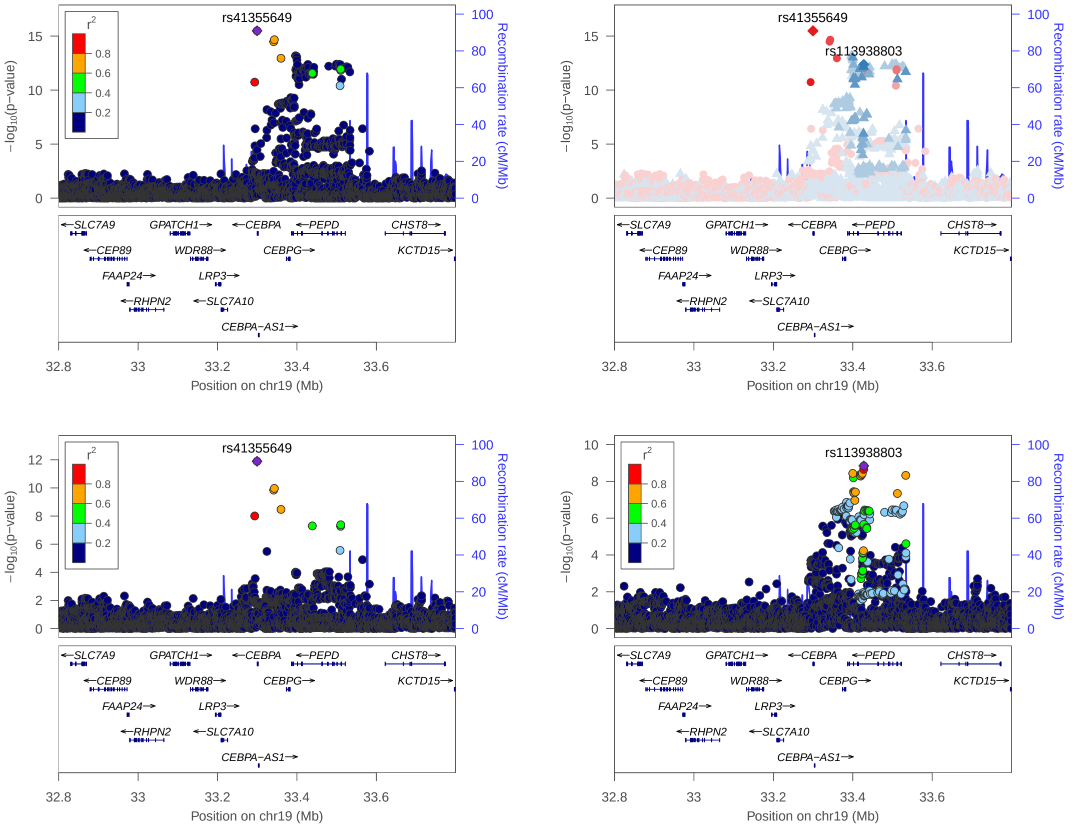
rs41355649
rs113938803
r2
0.8
0.6
0.4
0.2
−log10(p−value)
Recombination rate (cM/Mb)
←SLC7A9
GPATCH1→
←CEBPA
←PEPD
CHST8→
←CEP89
WDR88→
CEBPG→
KCTD15→
FAAP24→
LRP3→
←RHPN2
←SLC7A10
CEBPA−AS1→
32.8
33
33.2
33.4
33.6
Position on chr19 (Mb)
