# Supplementary material for: Genetic screens of imaging-derived kidney volumes identify genes linked to kidney function
Source: Kidney Int. Author manuscript; Available in PMC 2026 Jul 5. (PMC13333066; doi:10.1016/j.kint.2025.08.038)

# Sinus

## region chr1__2546929-3546929

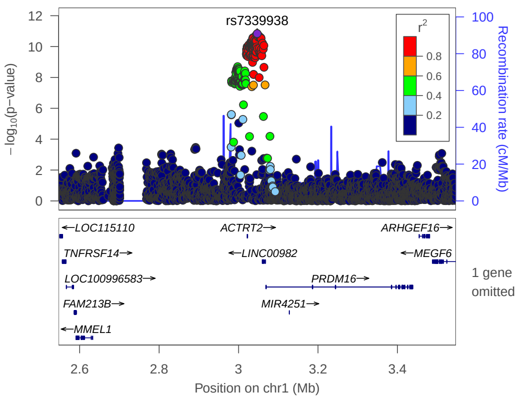


## region chr1__55608684-56608684

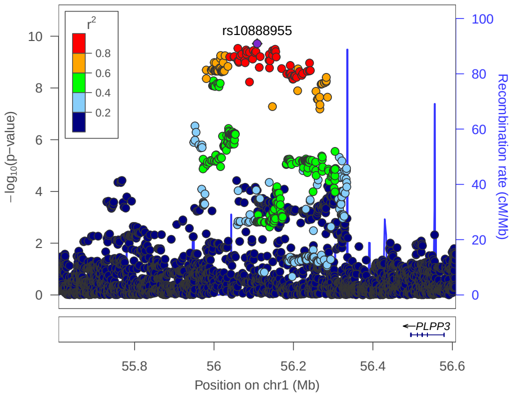


## region chr1__81989269-82989269

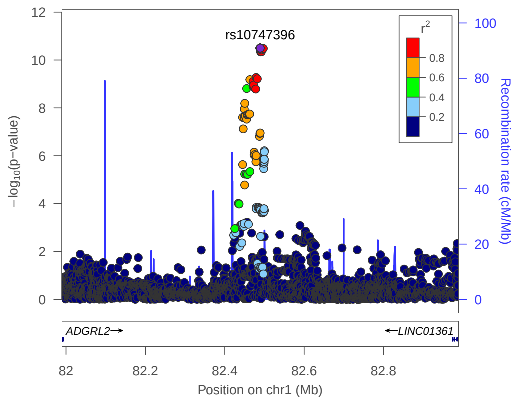


region chr1__163270480-164270480

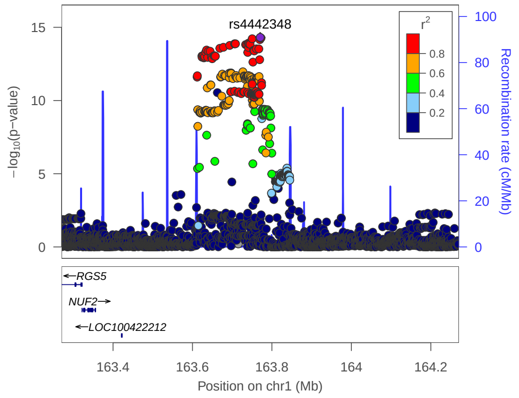


region chr1__164272351-165272351

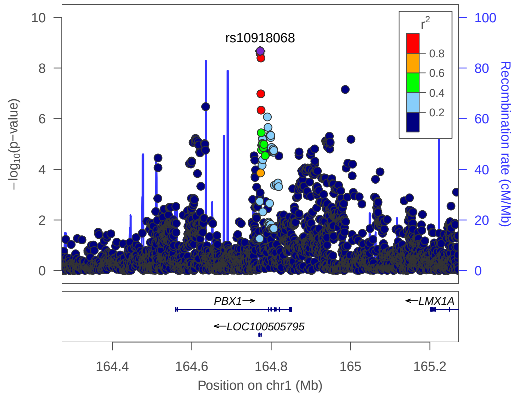


region chr1__222173540-223173540

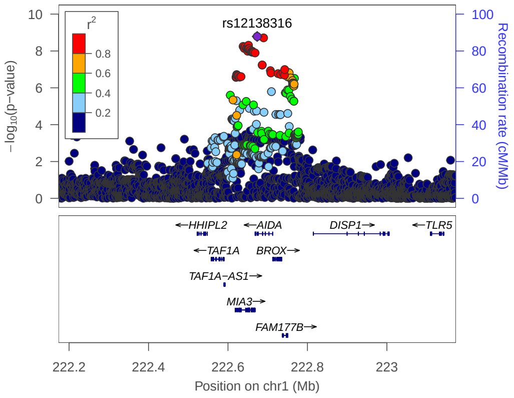


region chr1__234034101-235034101

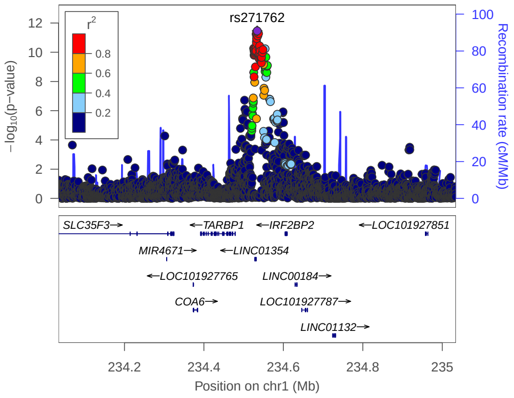


region chr2__5029060-6029060

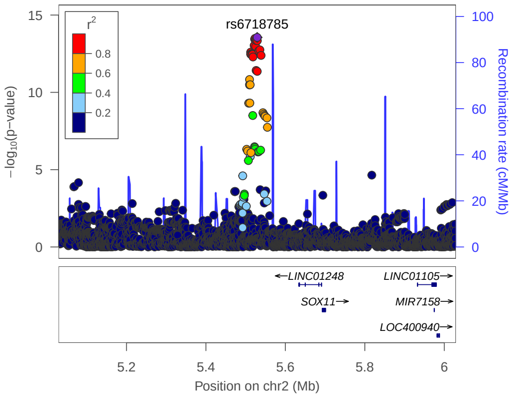


region chr2__64854552-65854552

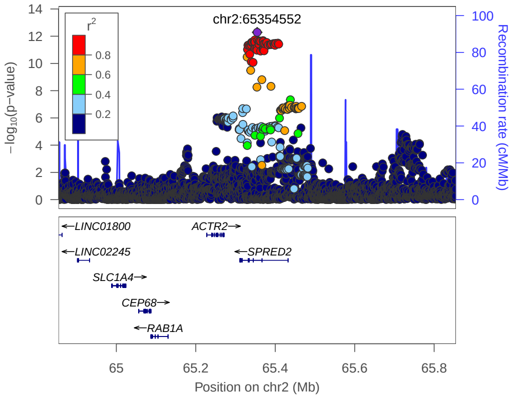


region chr2__144495857-145495857

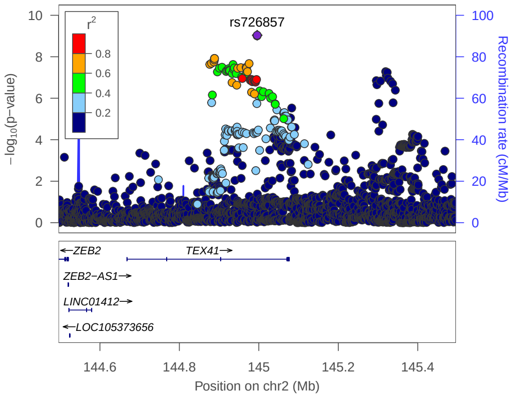


region chr2__147113960-148113960

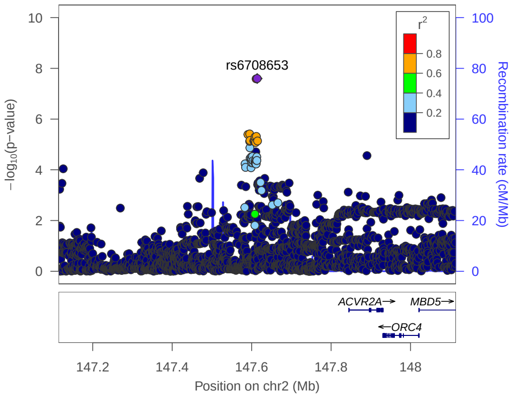


region chr2__149674625-150674625

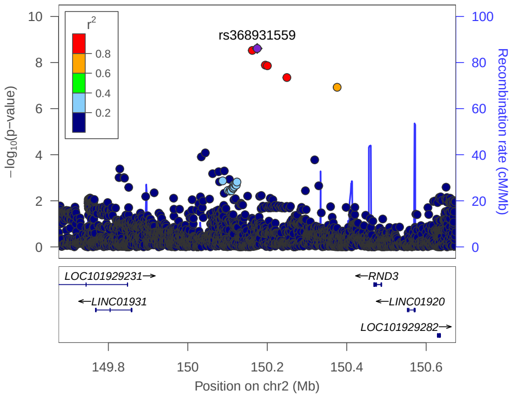


region chr2__158557430-159557430

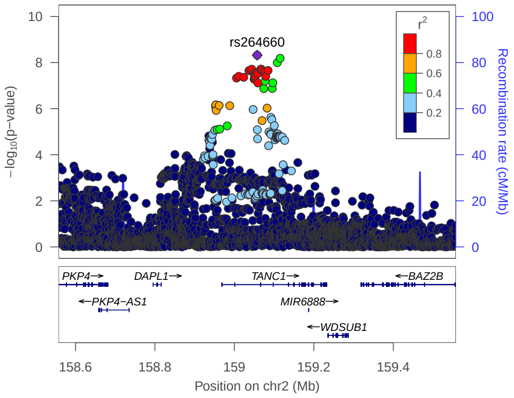


region chr2__238459613-239459613

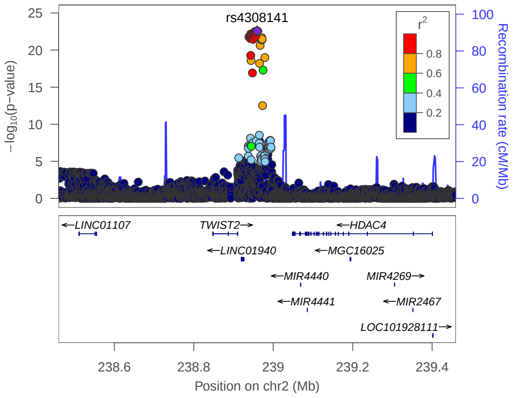


region chr3__11788284-12788284 has >1 independent SNPS

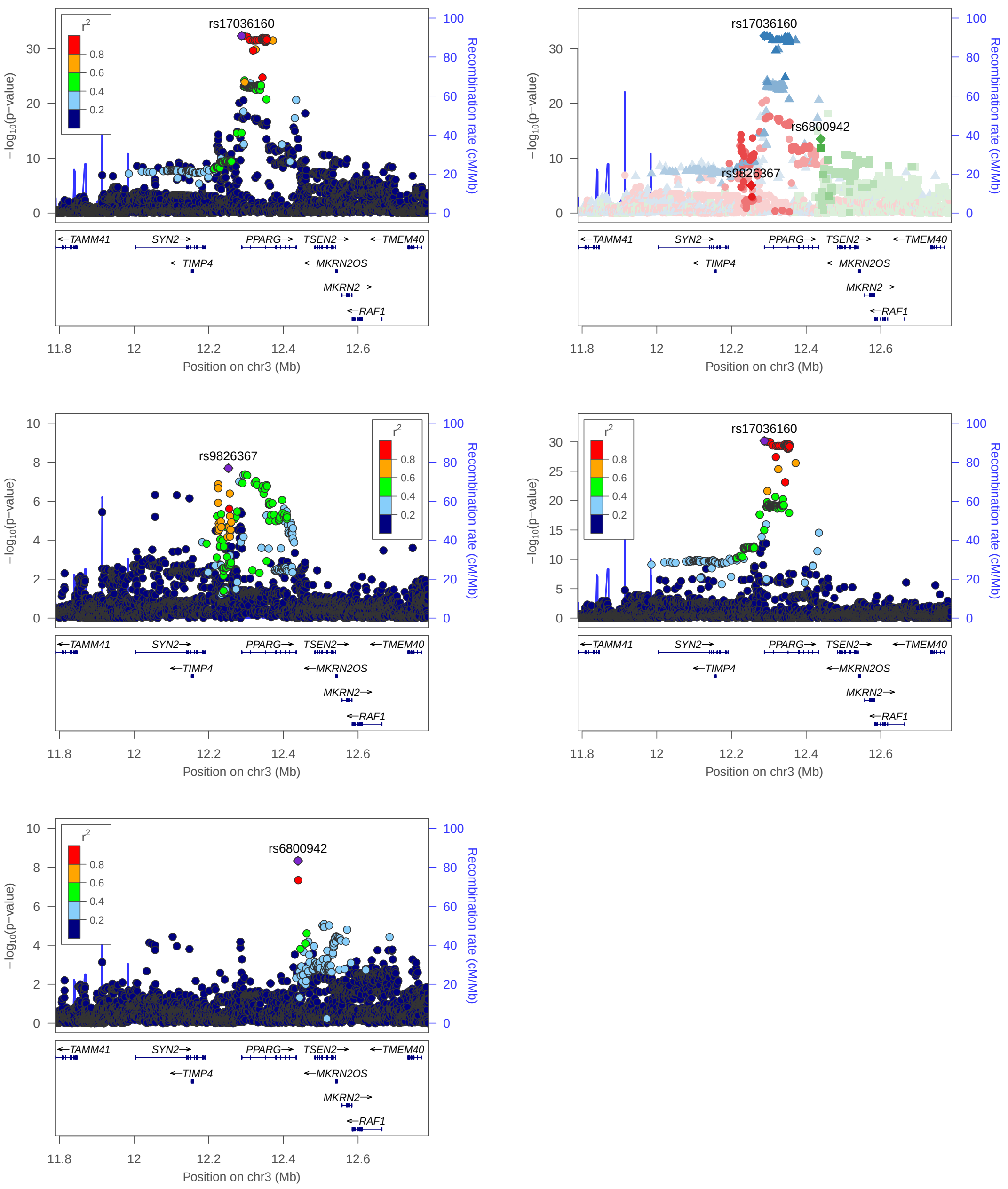


region chr4_18751406-19751406 has >1 independent SNPS

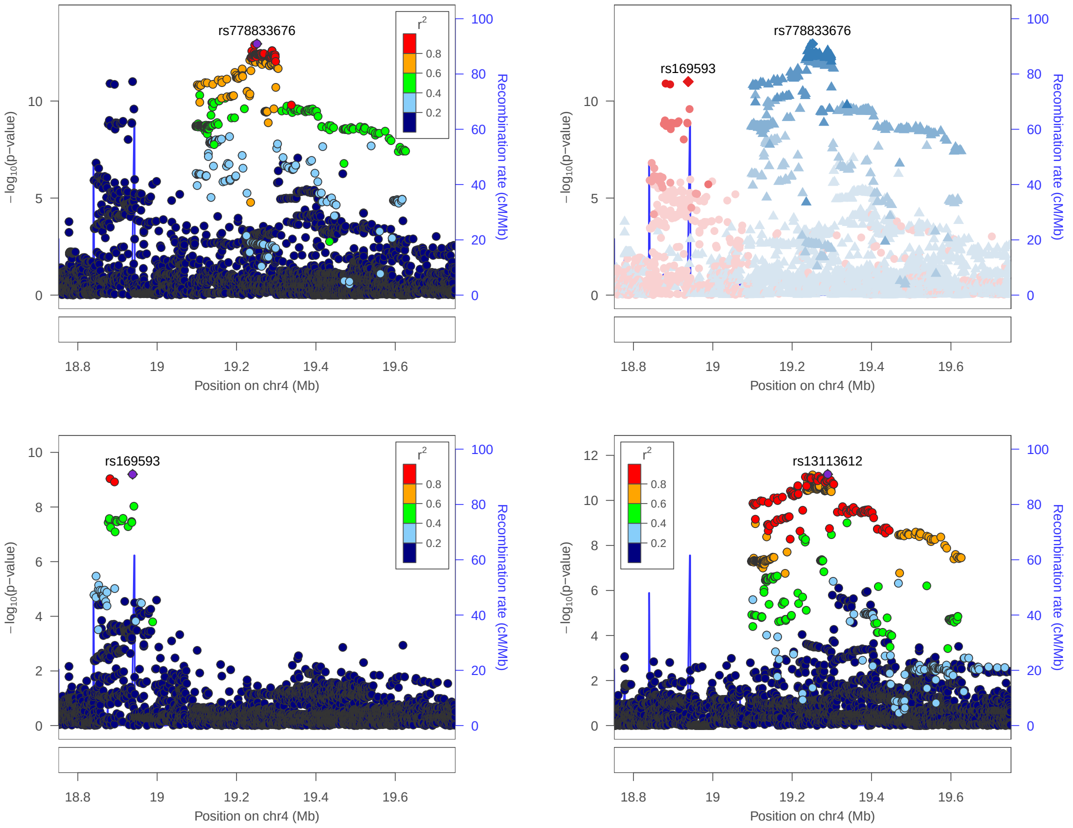


region chr4_85281720-86281720

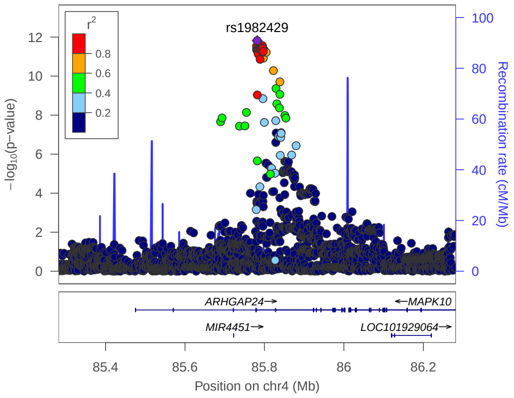

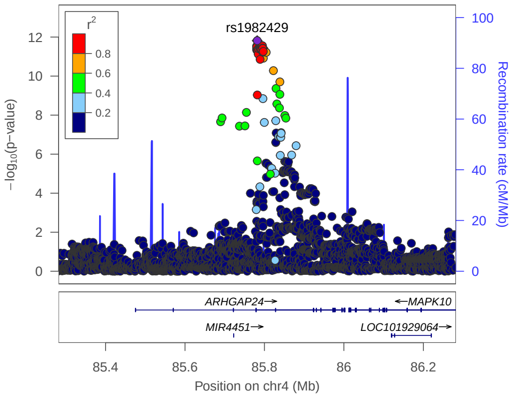


region chr4__93906507-94906507

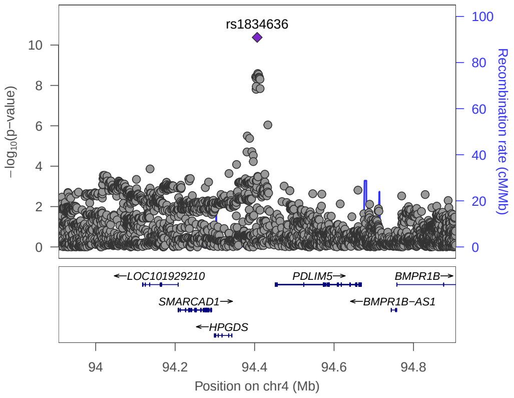


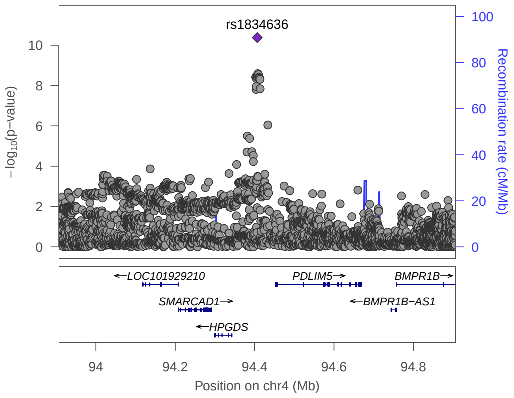


region chr4__144126033-145126033

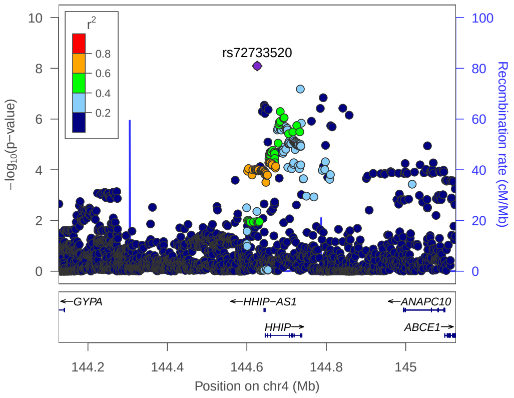


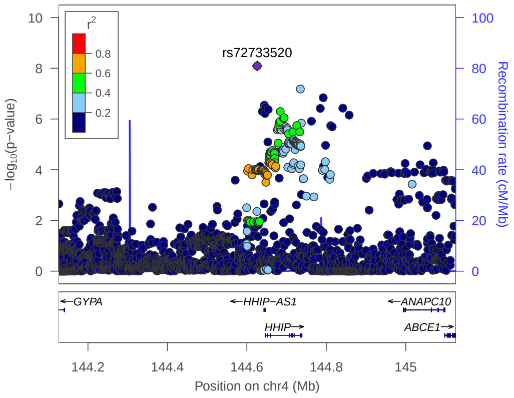


region chr4_176009493-177009493

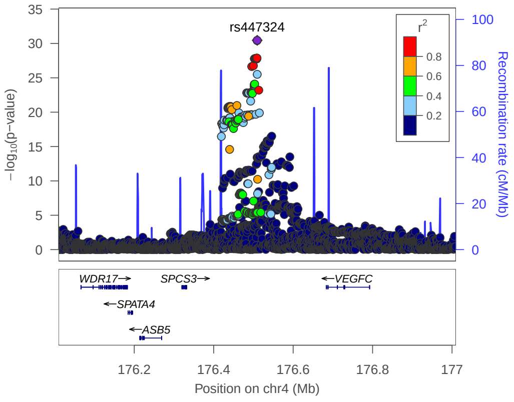

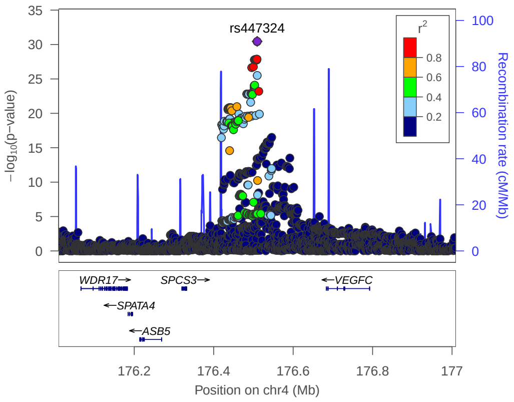


region chr5__37435651-38435651

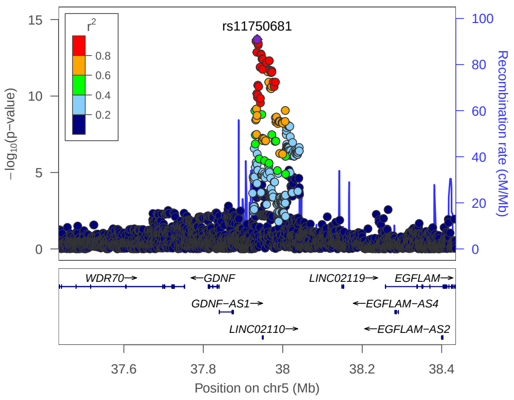


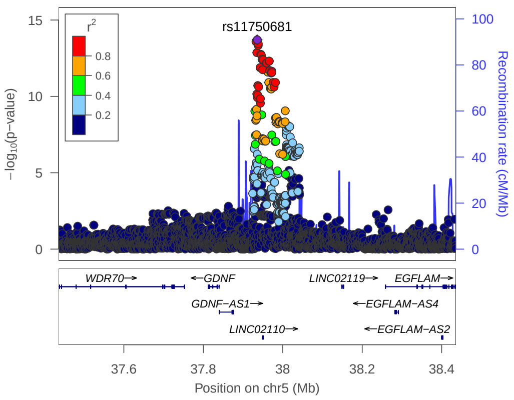


region chr5__39499080-40499080

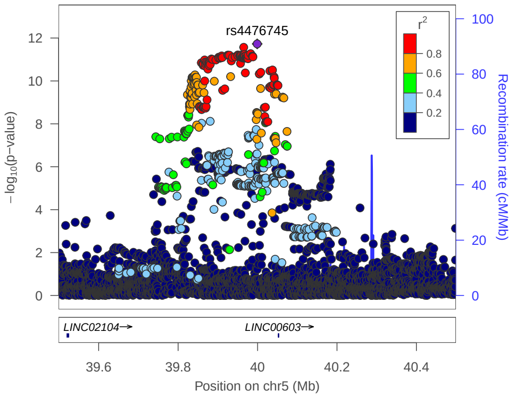


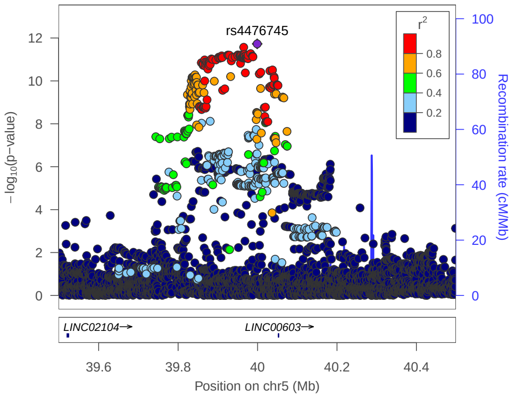


region chr5_43895913-45406888

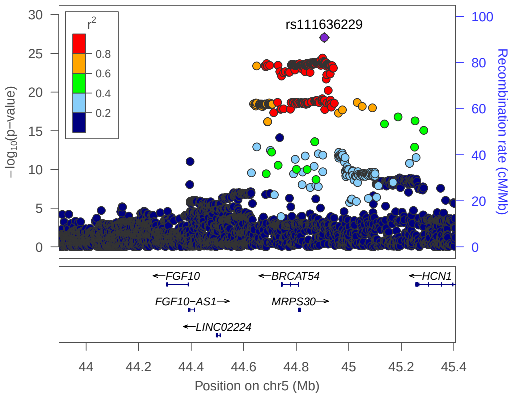

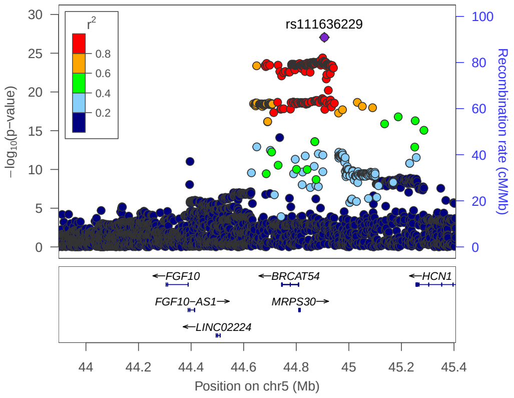


region chr5__50834166-51834166

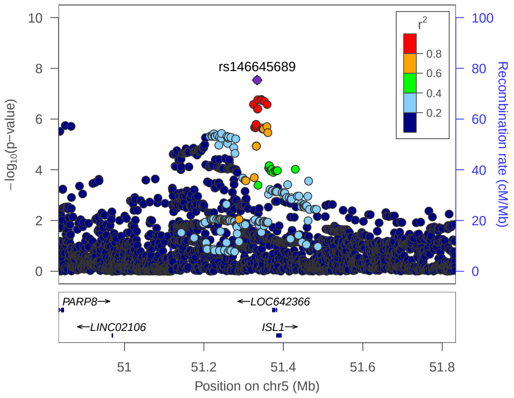


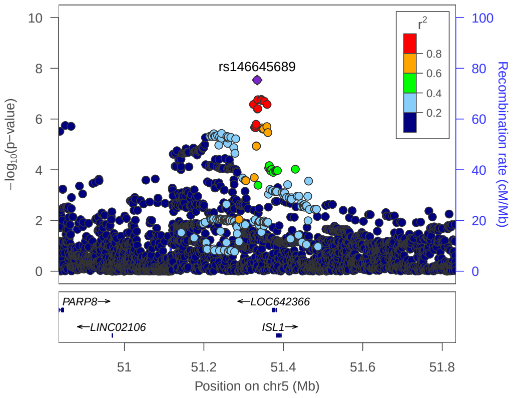


region chr5__52861272-54436536 has >1 independent SNPS

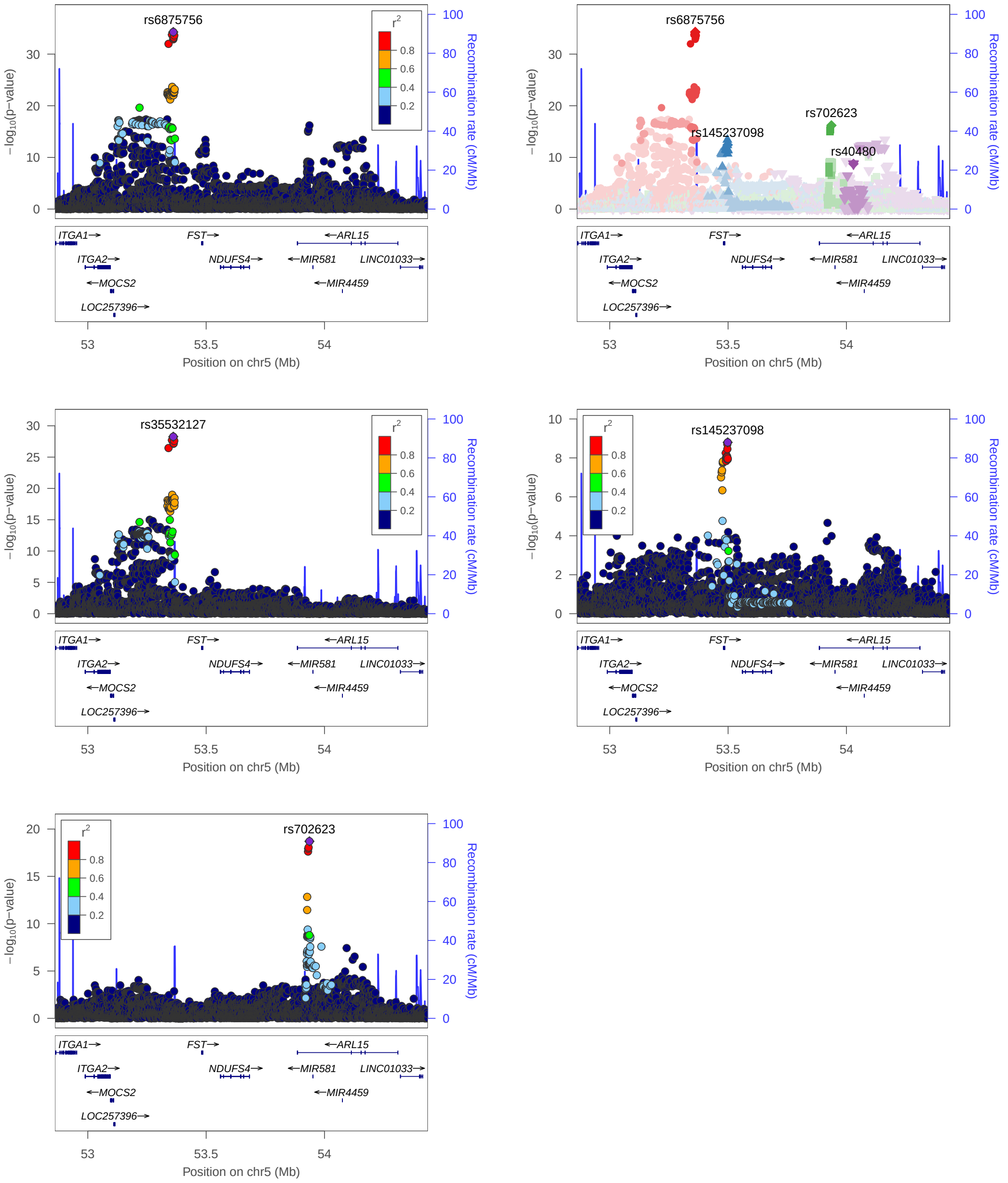
rs6875756
rs145237098
rs702623
rs40480
rs35532127
r2
0.8
0.6
0.4
0.2
−log10(p−value)
Recombination rate (cM/Mb)
ITGA1
FST
ARL15
ITGA2
NDUFS4
MIR581
LINC01033
MOCS2
MIR4459
LOC257396
53
53.5
54
Position on chr5 (Mb)

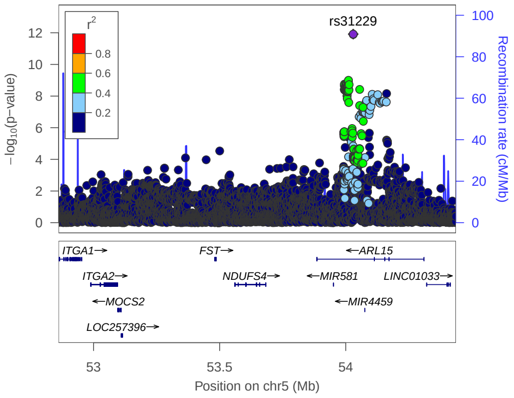


region chr5__56065080-57065080

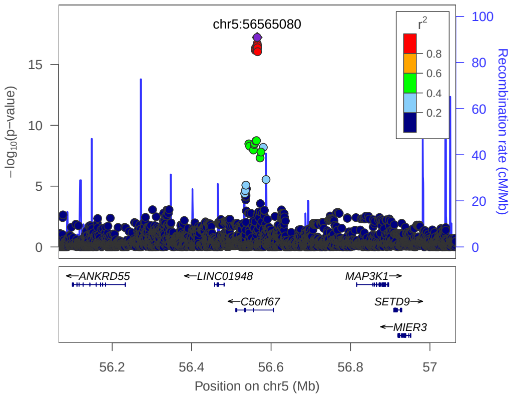


region chr5__64446116-65446116

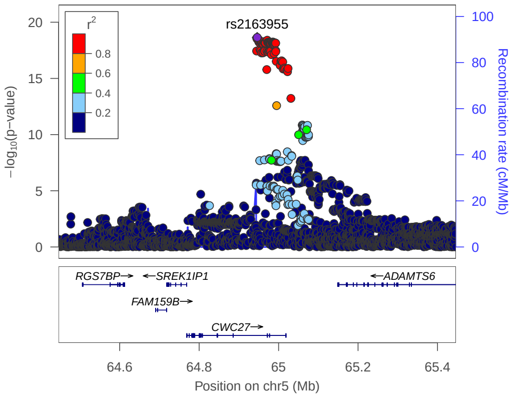


region chr5__72813005-73813005 has >1 independent SNPS

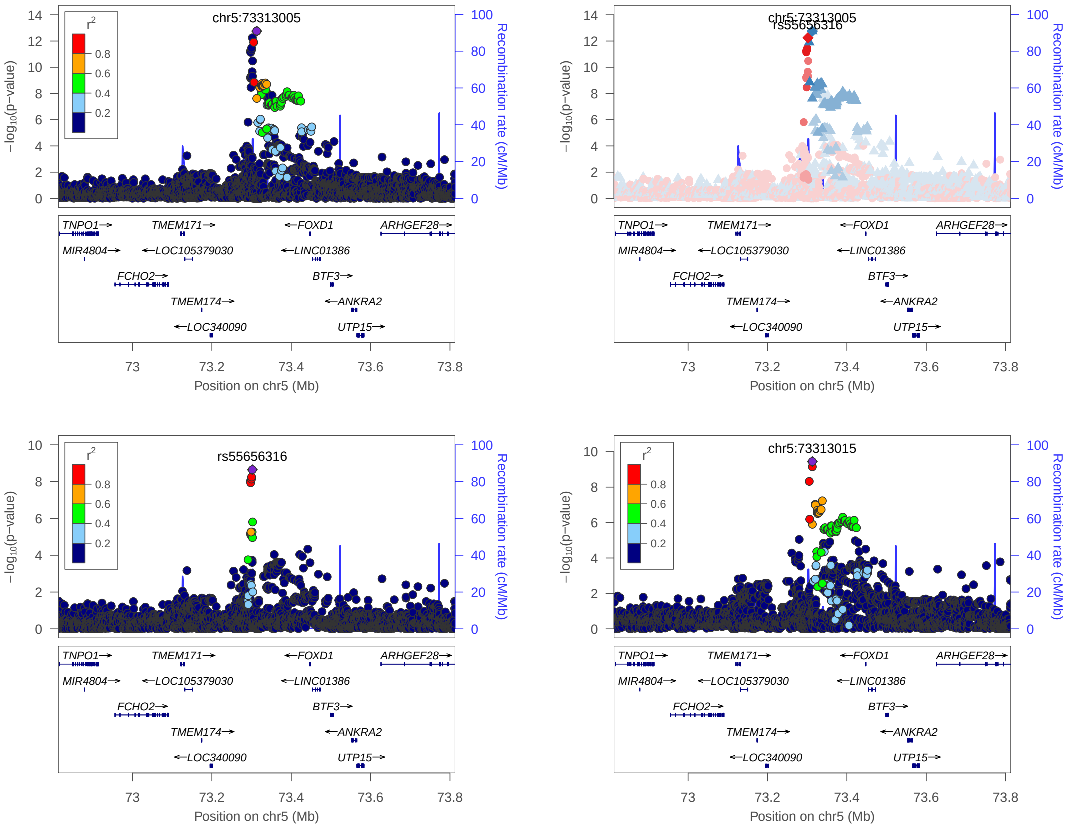


## region chr5__158263428-159263428

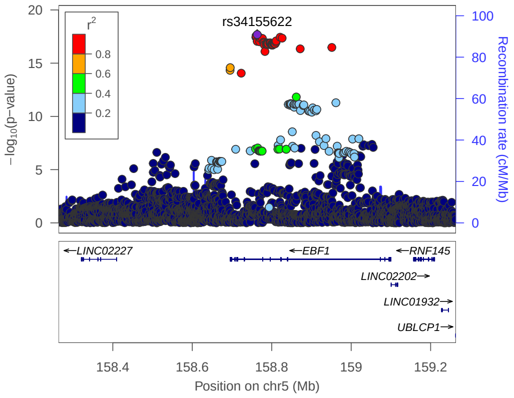

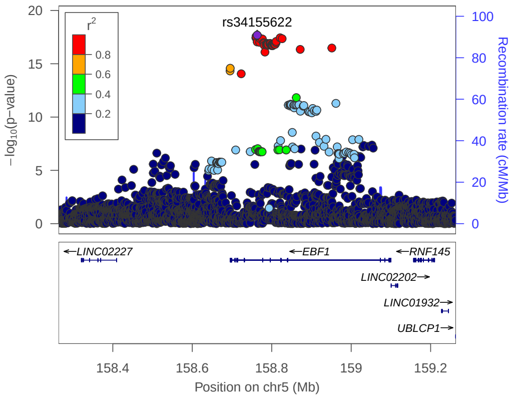


region chr5__168567909-169567909

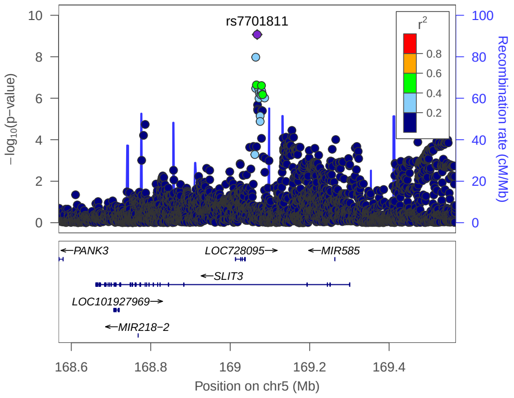


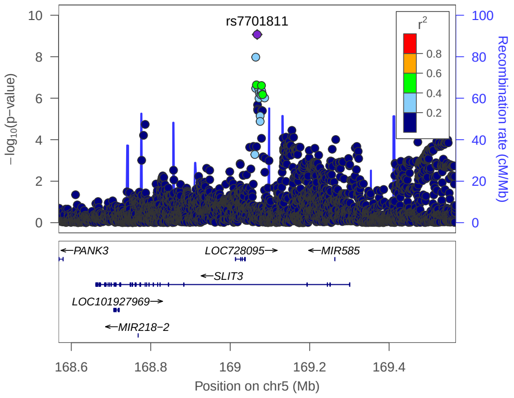


region chr6__6238519-7238519

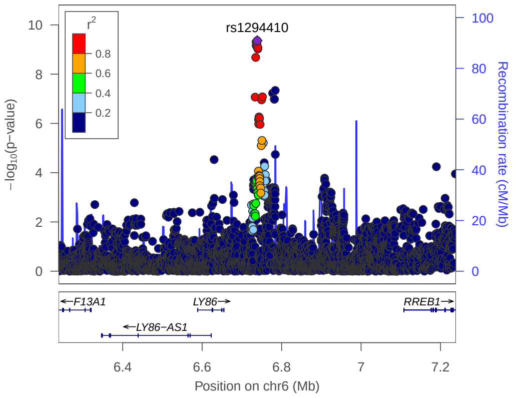


region chr6__21597146-22597146

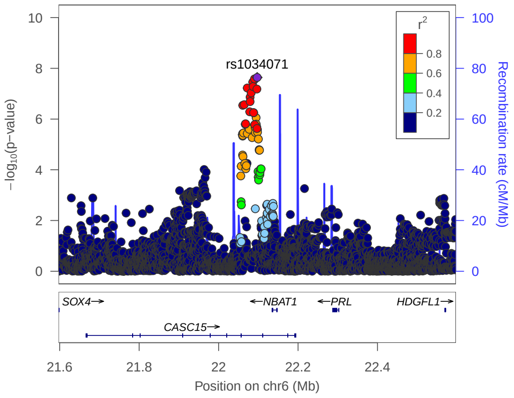


region chr6__83799805-84799805

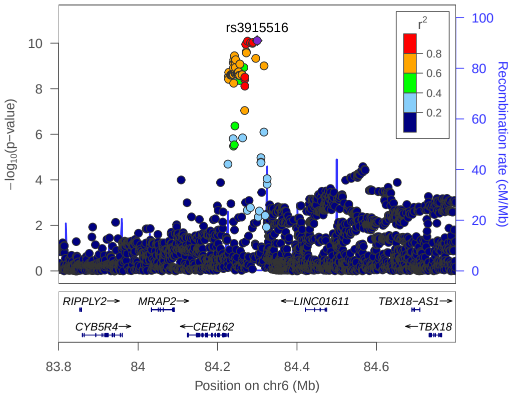


region chr6__133350341-134350341

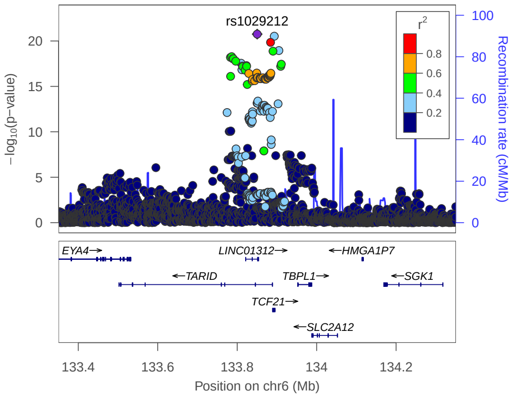


region chr7__844934-1844934

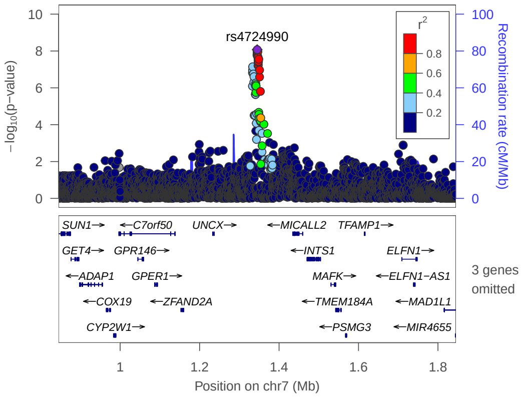


region chr7__27816085-28816085 has >1 independent SNPS

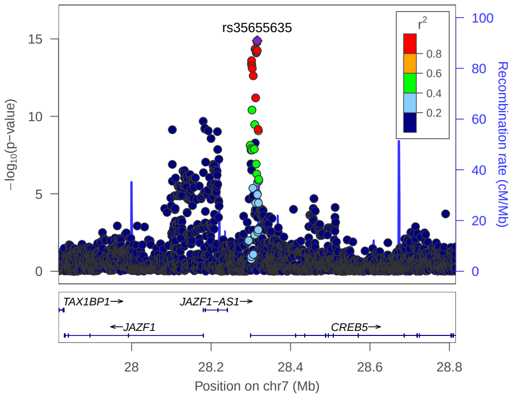


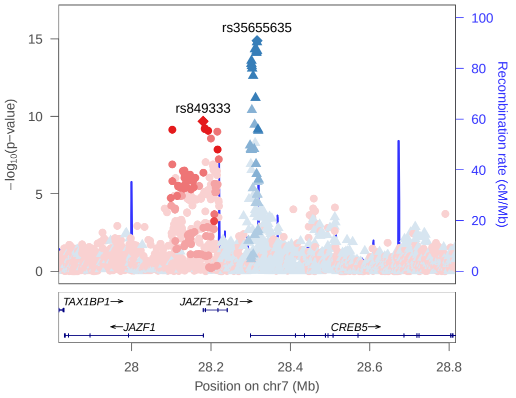

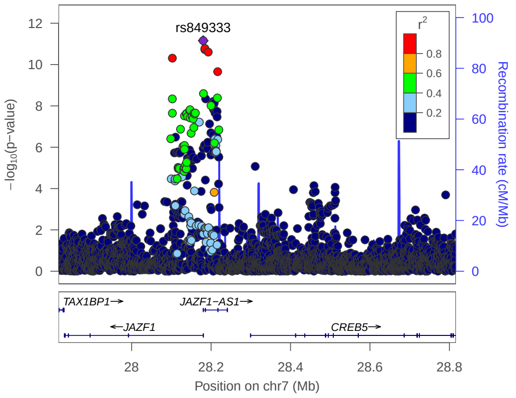


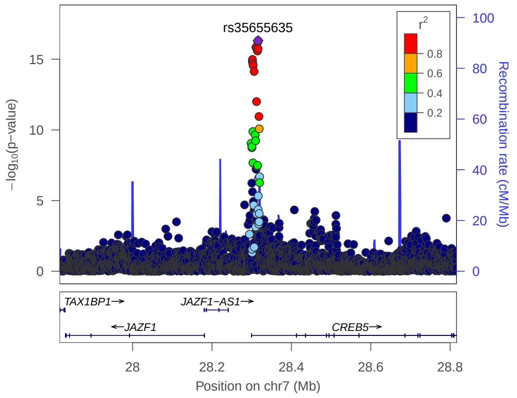


region chr7__30417533-31417533

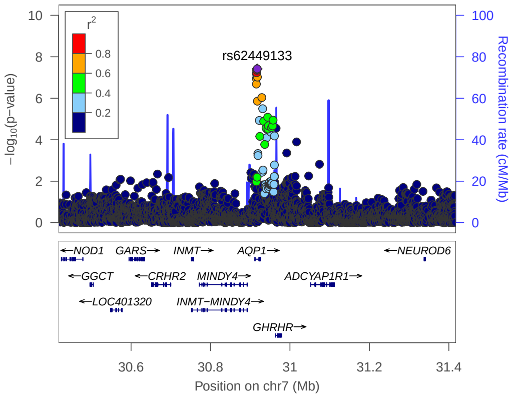


region chr7__39861113-40861113

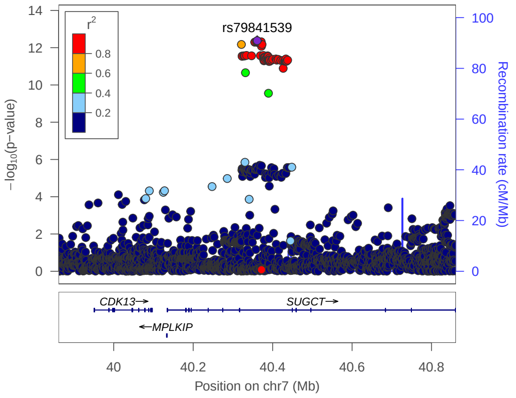


region chr7__84317169-85317169

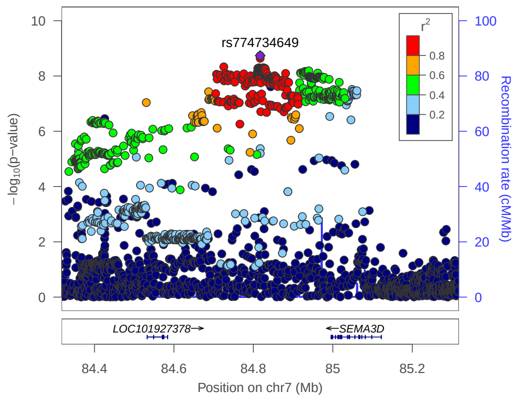


region chr8__13083174-14083174

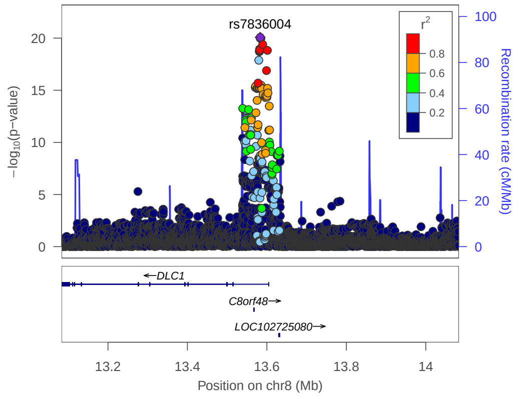


region chr8__23357479-24357479

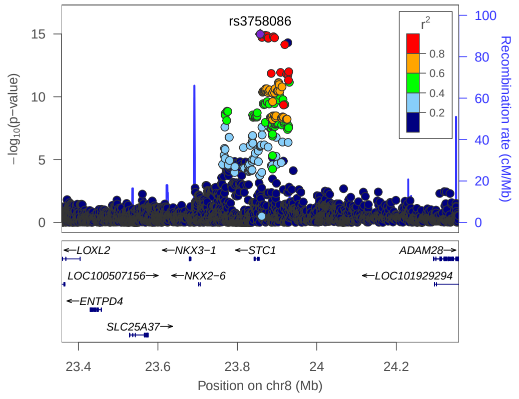


region chr8__25059140-26059140

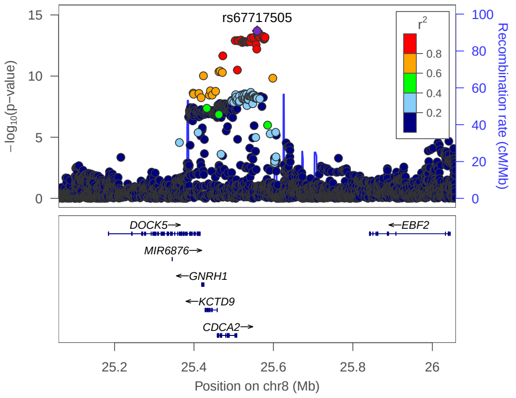


region chr8__104437870-105437870 has >1 independent SNPS

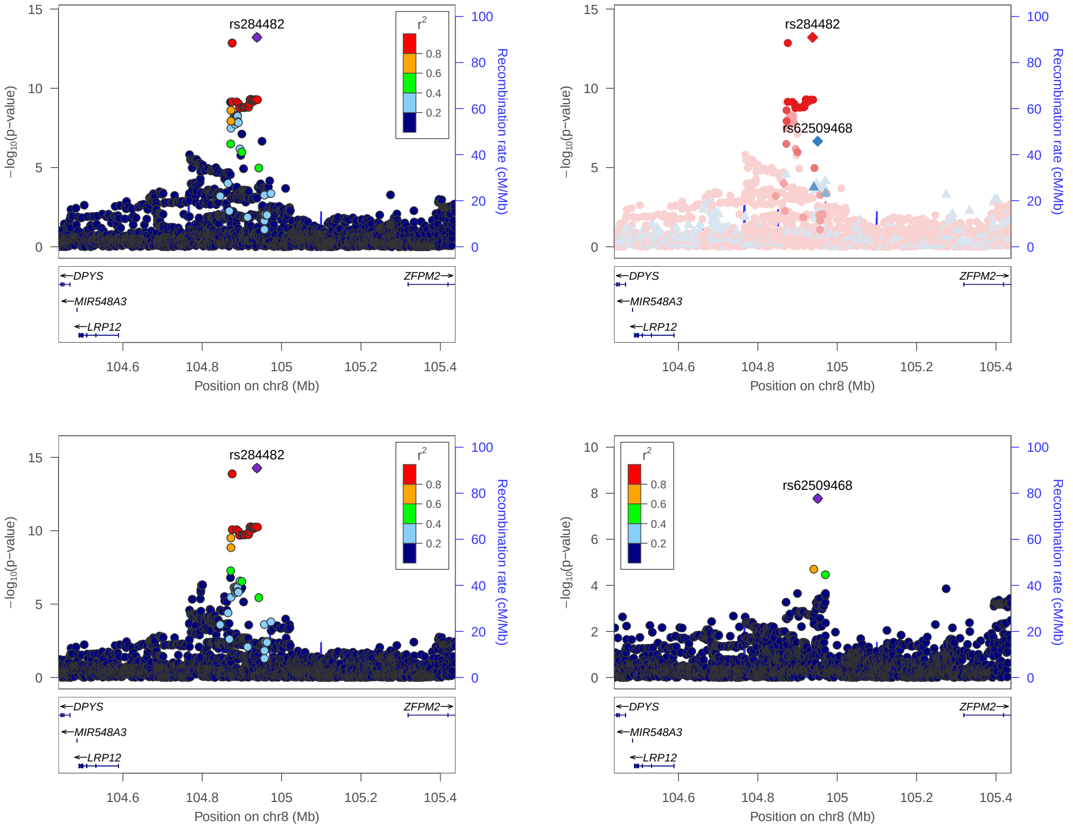


region chr9__13479873-14479873

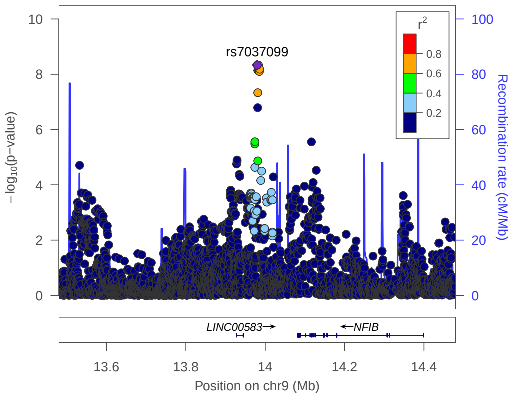


region chr9__20093367-21093367

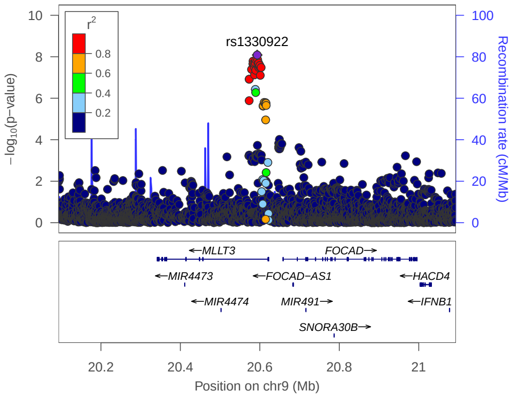


region chr9__107474633-108474633 has >1 independent SNPS

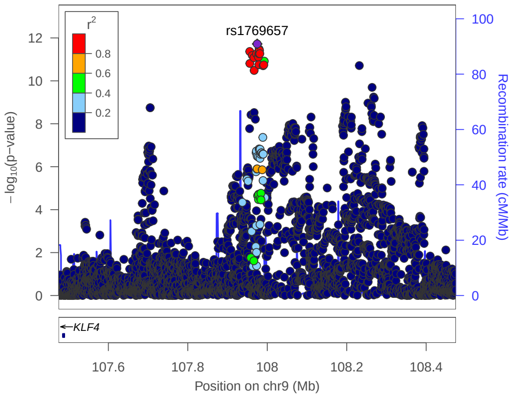


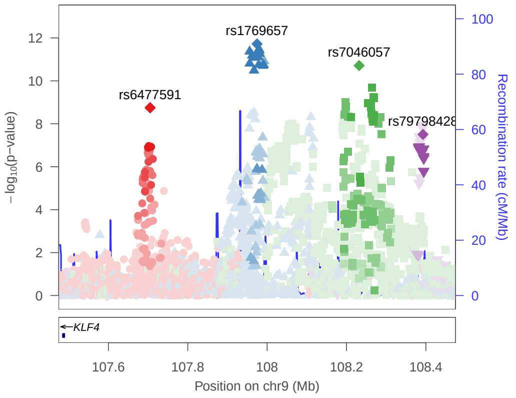

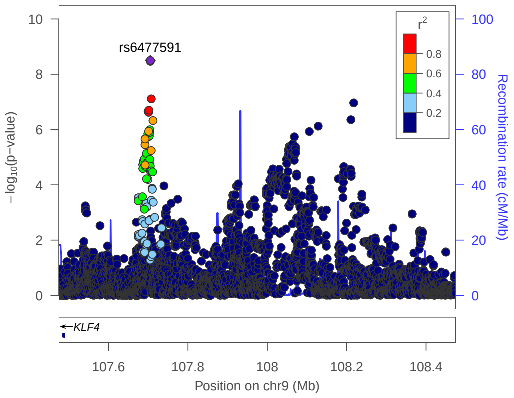


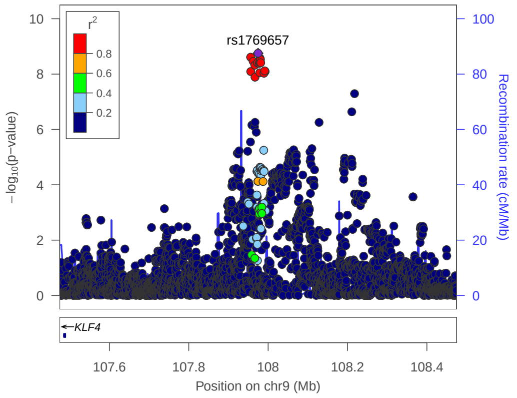


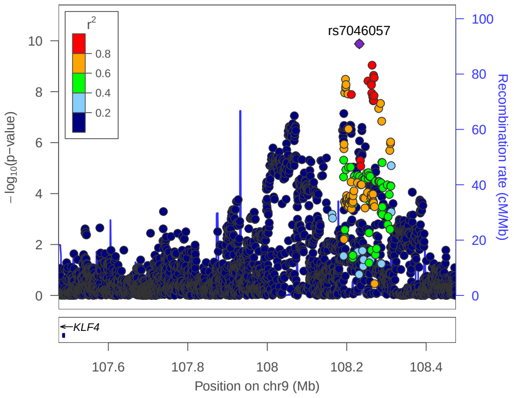


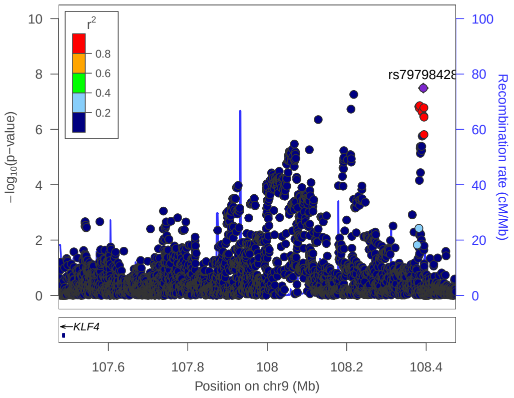


region chr9_110682766-111682766

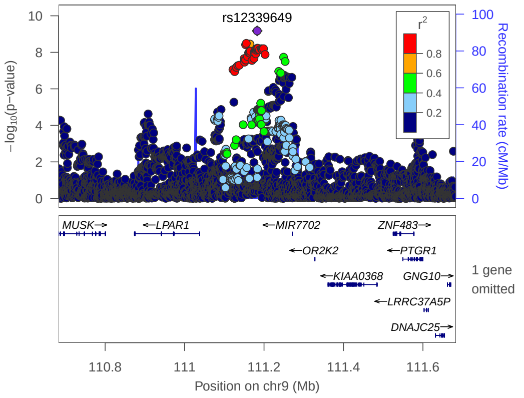


region chr10__102101694-103101694

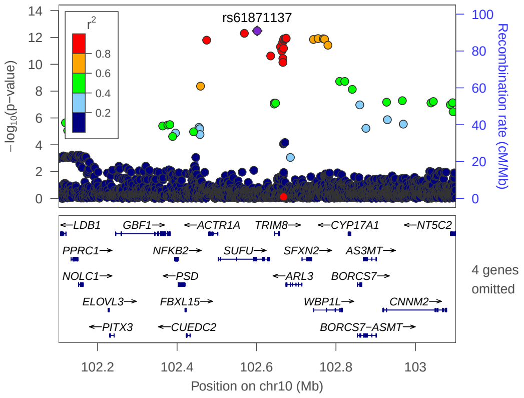


region chr10__128632557-130142985 has >1 independent SNPS

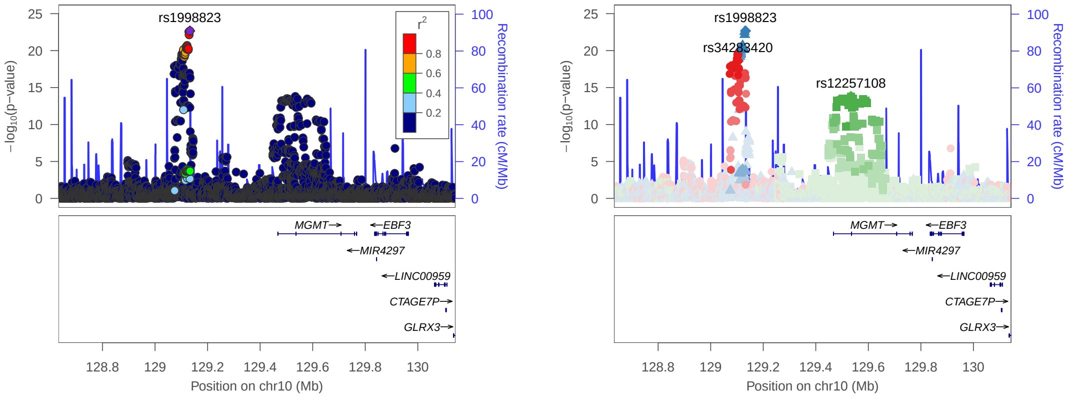

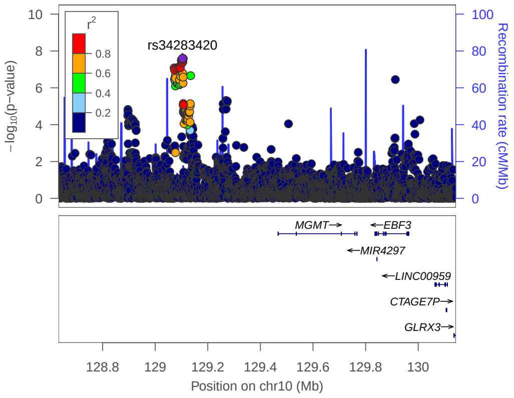


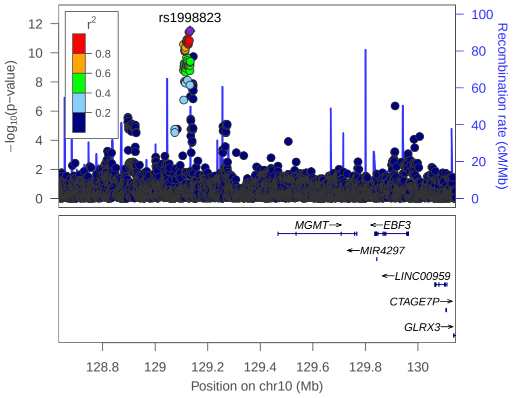


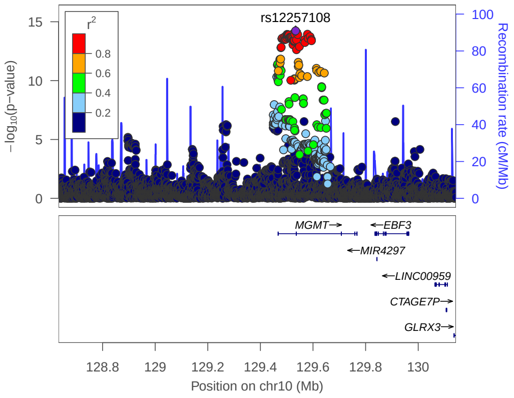


region chr11__121430263-122430263

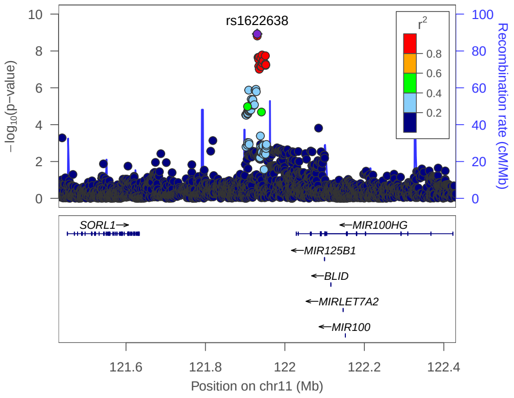


region chr12__19515744-20515744

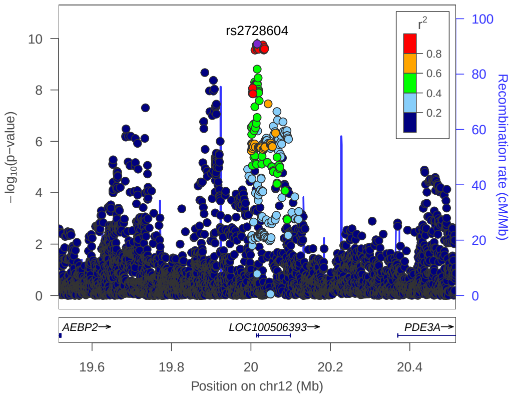


region chr12__76652640-77652640

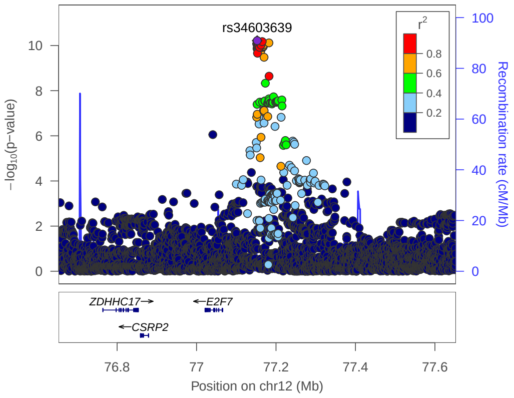


region chr12__114560978-115560978 has >1 independent SNPS

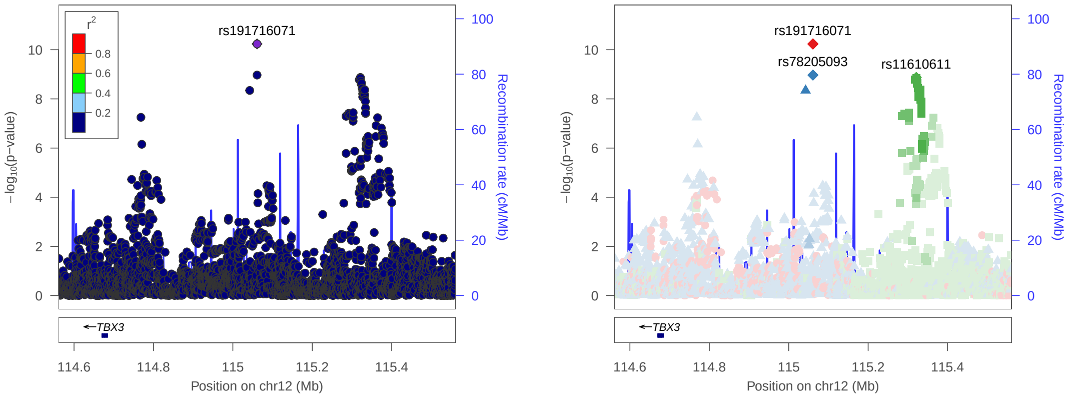

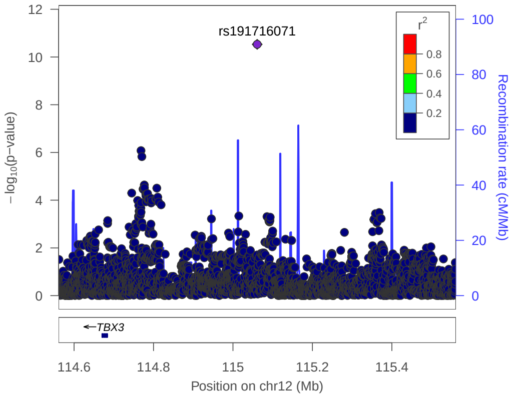


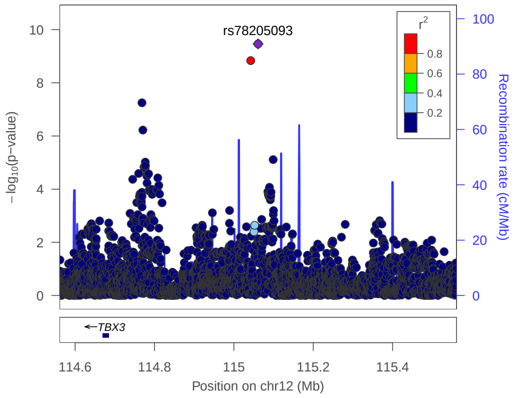


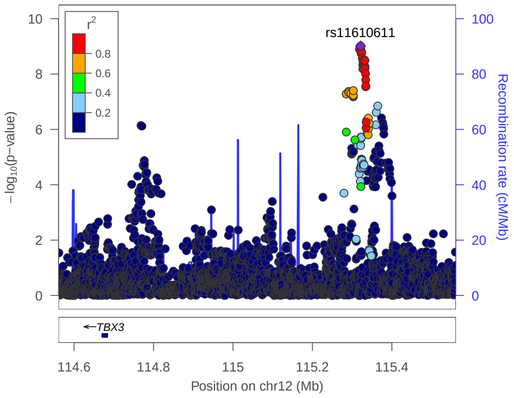


region chr13_21402149-22402149

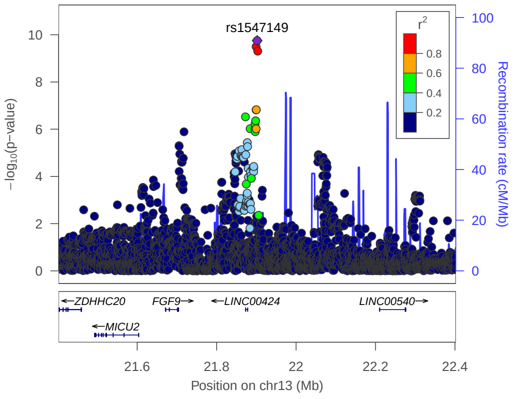


region chr13_49955992-50955992

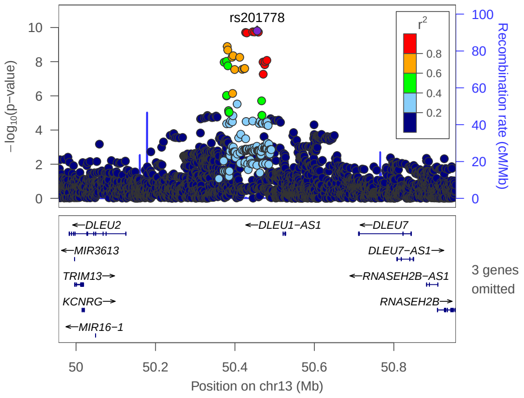


region chr13_92744682-93744682

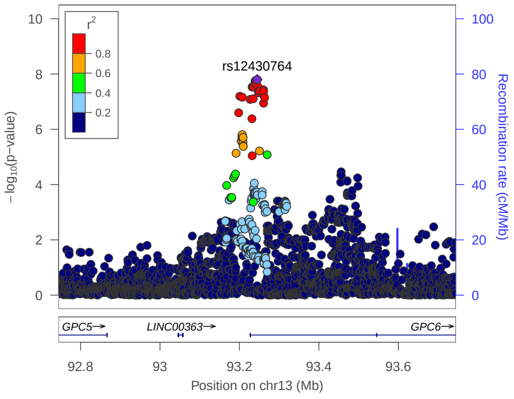


region chr15_32217277-33217277 has >1 independent SNPS

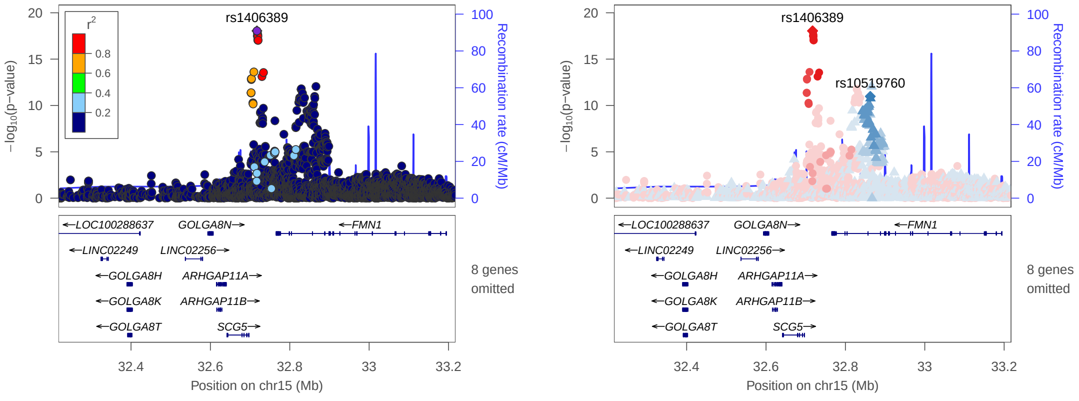

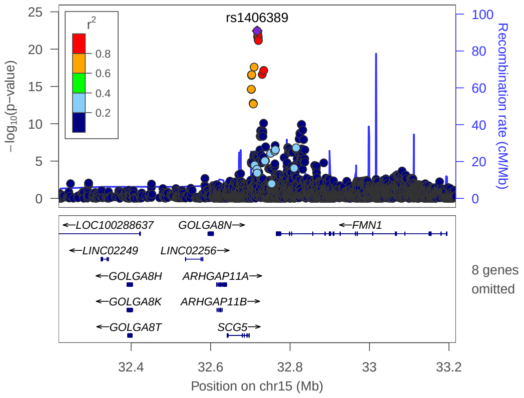


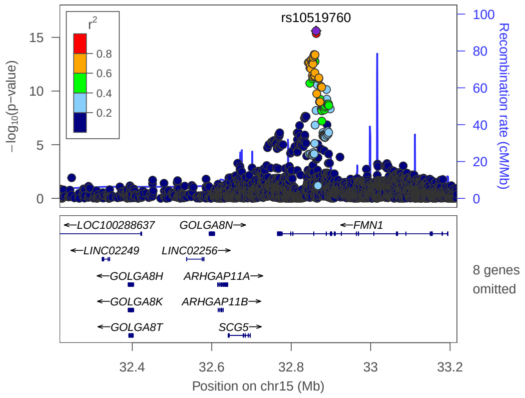


region chr15__66679847-68284145

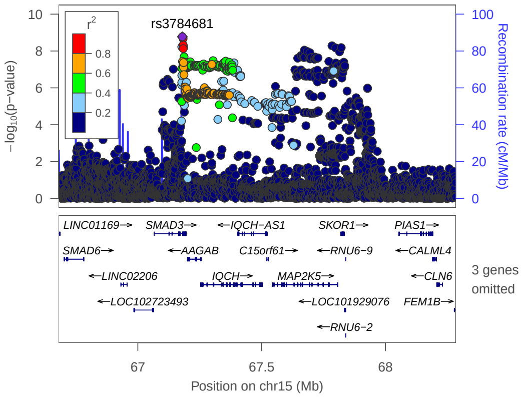


region chr15__98245330-99245330

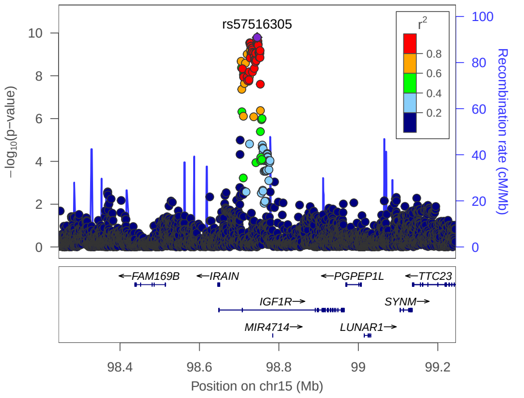


region chr16__3806205-4806205

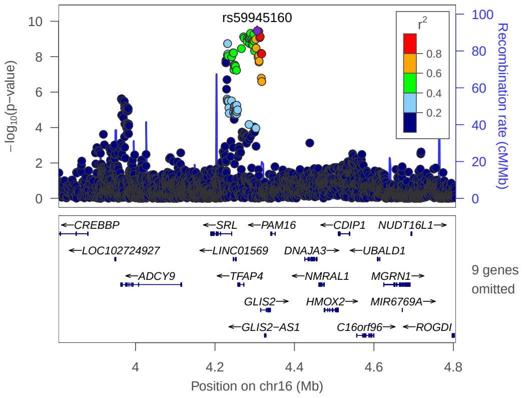


region chr16__78226115-79226115

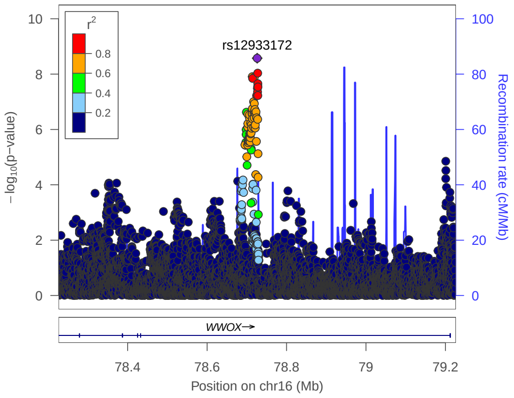


region chr18__50122643-51122643

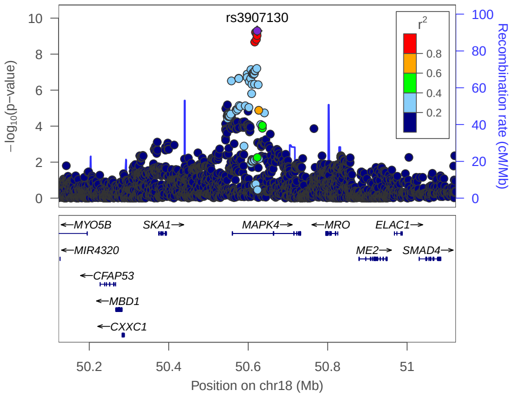


region chr18__59011105-60011105

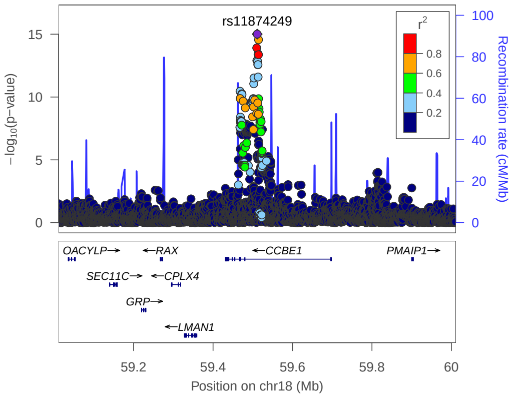


region chr19__13971727-14971727

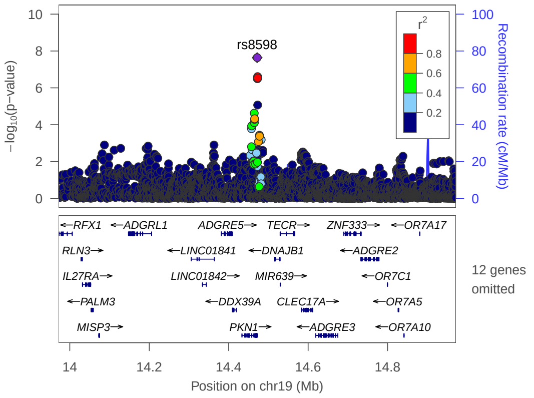


region chr19__32898094-33898094 has >1 independent SNPS

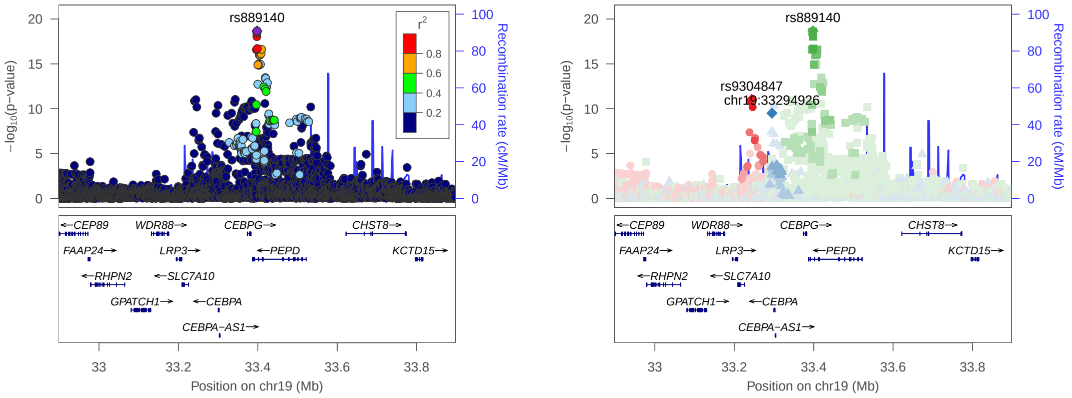

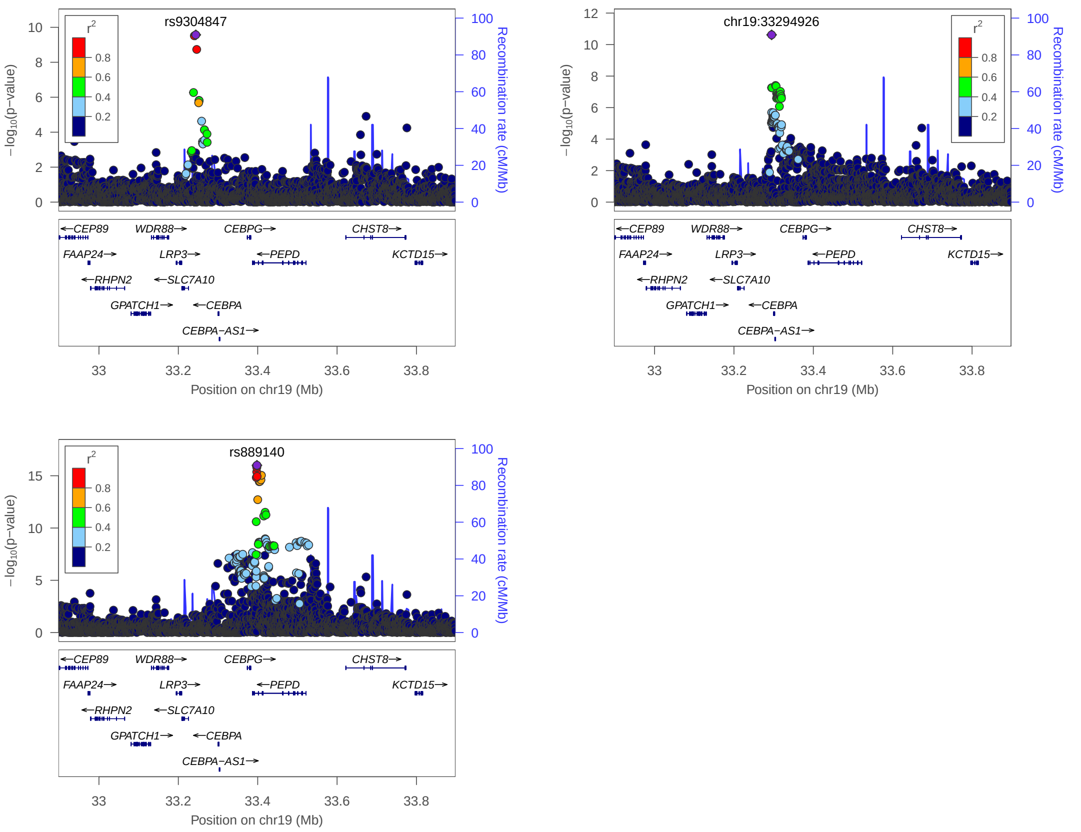


region chr20_3762610-4762610 has >1 independent SNPS

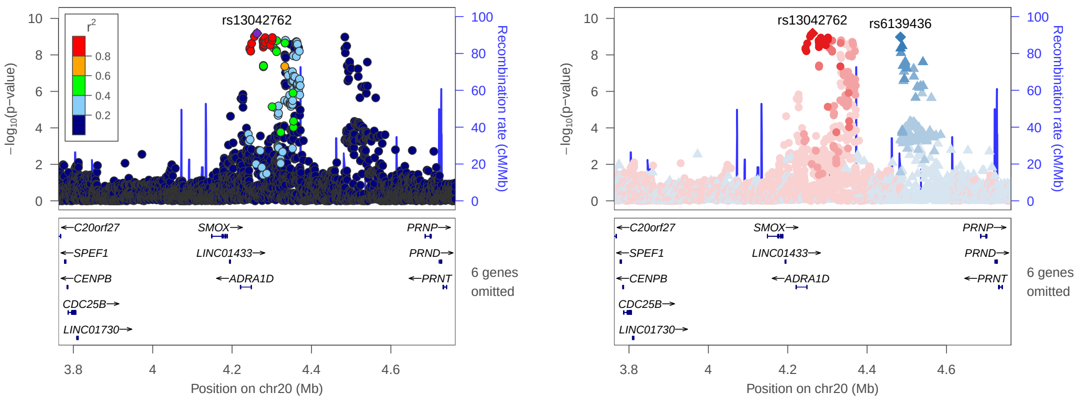

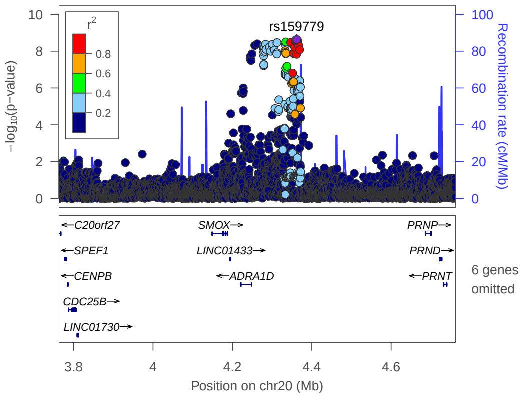


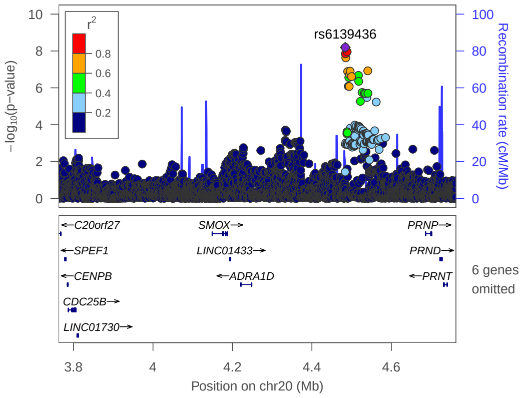


region chr20__10179415-11179415

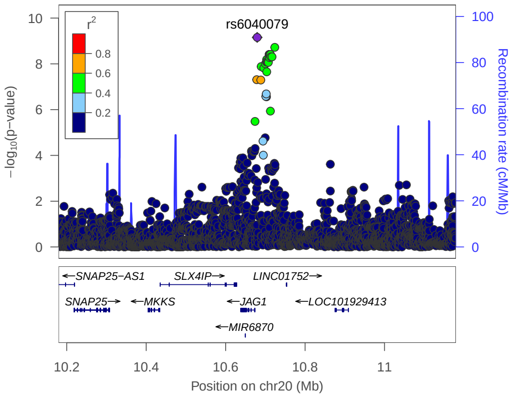


region chr20__40659297-41659297

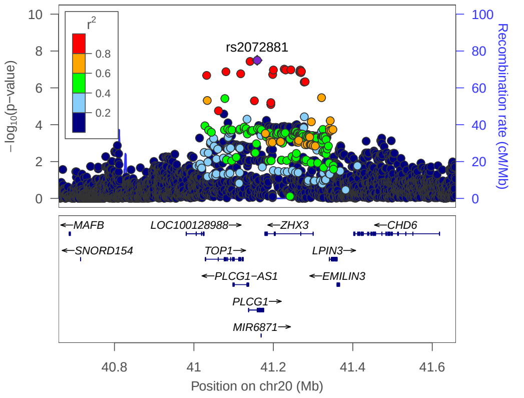


region chr20__61944330-62944330

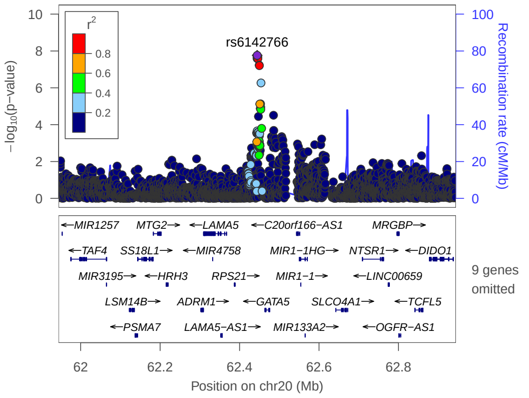


## region chr21__33721526-34721526

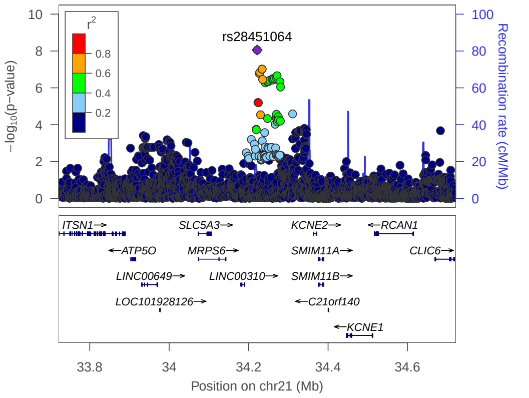


## region chr22__37284400-38284400

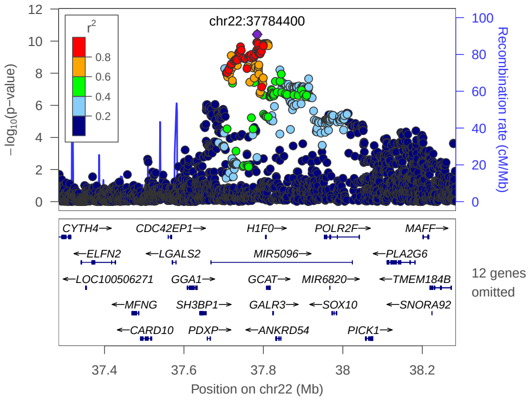

Supplement: 5 [file NIHMS2184648-supplement-5.pdf]
